# Formal [3 + 2] Cycloaddition of *α*-Imino Esters with Azo Compounds: Facile Construction of Pentasubstituted 1,2,4-Triazoline Skeletons

**DOI:** 10.3390/molecules28114339

**Published:** 2023-05-25

**Authors:** Yasushi Yoshida, Hidetoshi Ida, Takashi Mino, Masami Sakamoto

**Affiliations:** Molecular Chirality Research Center, Graduate School of Engineering, Chiba University, 1-33, Yayoi-cho, Inage-ku, Chiba-shi 263-8522, Japan

**Keywords:** cycloaddition, *α*-imino ester, azo compound, 1,2,4-triazoline, 1,2,4-triazole

## Abstract

1,2,4-Triazole and 1,2,4-triazoline are important components of bioactive molecules and catalysts employed in organic synthesis. Therefore, the efficient synthesis of these components has received significant research attention. However, studies on their structural diversity remain lacking. Previously, we developed chiral phase-transfer-catalyzed asymmetric reactions of *α*-imino carbonyl compounds with *α*,*β*-unsaturated carbonyl compounds and haloalkanes. In this study, we demonstrate the formal [3 + 2] cycloaddition reaction of *α*-imino esters with azo compounds under Brønsted base catalysis, resulting in the corresponding 1,2,4-triazolines in high yields. The results revealed that a wide range of substrates and reactants can be applied, irrespective of their steric and electronic characteristics. The present reaction made the general preparation of 3-aryl pentasubstituted 1,2,4-triazolines possible for the first time. Furthermore, a mechanistic study suggested that the reaction proceeds without isomerization into the aldimine form.

## 1. Introduction

**Figure 1:**
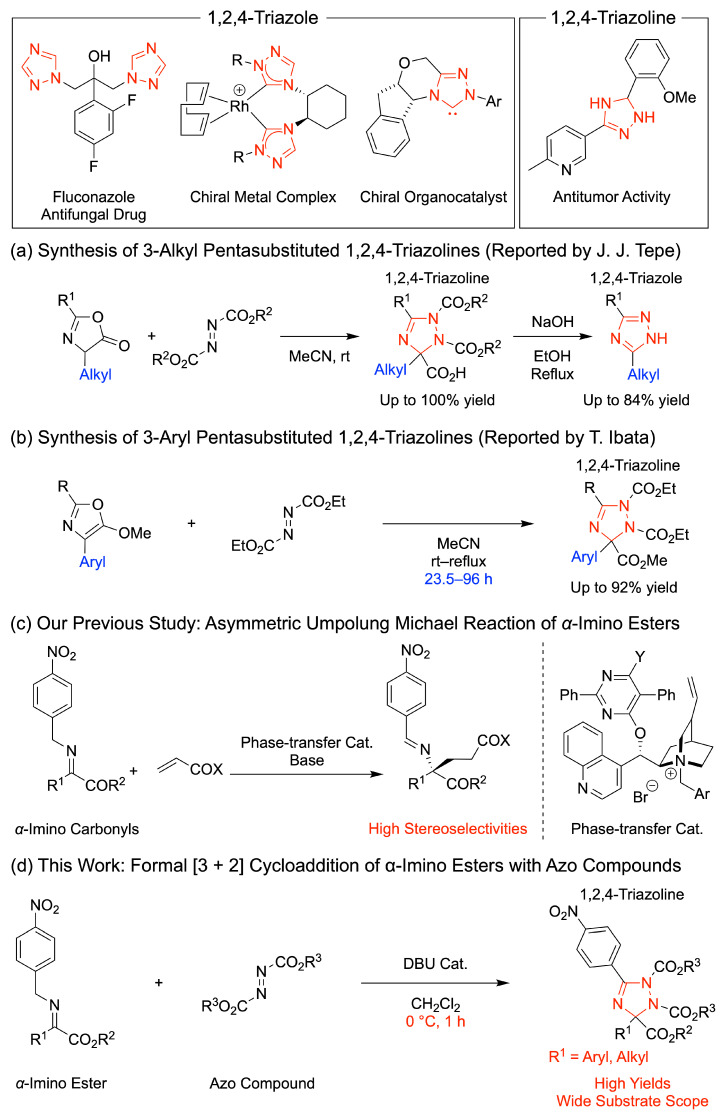
Useful molecules bearing 1,2,4-triazole and 1,2,4-triazoline skeleton and synthesis of 1,2,4-triazolines in (**a**) Tepe’s study [19], (**b**) Ibata’s study [20], (**c**) our previous study [36,37,38,39,40], and (**d**) present work.

1,2,4-Triazoles are fundamental core components in biologically active molecules, such as fluconazole and voriconazole (Figure 1) [1,2,3,4]. They are also employed in chiral ligands as well as metal and organocatalysts, such as chiral biscarbene ligands [5], 1,2,4-triazole anion catalysts [6], and Rovis catalysts [7]. Efficient methods for preparing 1,2,4-triazoles have been extensively investigated, and they are mainly synthesized via the Cu-catalyzed oxidative reaction of 2-aminopyridines with nitriles [8], C–H amidation/cyclization of azomethine imines [9], intramolecular oxidative N–N bond formation [10], electrochemical oxidation [11], and other methods [12,13,14,15,16,17].

1,2,4-Triazoline is also an important motif owing to its wide utility as a biologically active compound, including as an antitumor-active molecule [18]. Furthermore, it is a useful precursor for synthesizing 1,2,4-triazole [19]. Therefore, efficient synthesis methods for 1,2,4-triazolines have been investigated [19,20,21,22,23,24,25,26]. In 2017, Li, Tang, and co-workers reported the visible-light-induced cyclization of azirines with azodicarboxylate, which formed the corresponding 1,2,4-triazolines in high yields [26]. Although synthetic methods for 1,2,4-triazolines have been developed, candidates with pentasubstituted structures have rarely been synthesized under metal-free conditions. In 2010, Tepe and co-workers prepared the 3-alkyl pentasubstituted 1,2,4-triazolines by the conjugate addition of oxazolones with azodicarboxylate, resulting in corresponding products in 50–100% yield (Figure 1a) [19]. Although an efficient synthesis method for the exclusive preparation of 3-alkyl pentasubstituted 1,2,4-triazolines has been developed, their 3-aryl-substituted compounds are rarely synthesized. In 1992, Ibata and co-workers reported the abnormal Diels–Alder reaction of oxazoles with a diethyl azodicarboxylate, which formed pentasubstituted 1,2,4-triazolines in 25–92% yield with a longer reaction time of more than 23.5 h (Figure 1b) [20]. Therefore, the development of a general and facile method for the metal-free preparation of 3-aryl pentasubstituted 1,2,4-triazolines is highly desirable.

*α*-Imino esters are useful molecular scaffolds owing to their widespread application as electrophiles [27,28,29,30,31,32,33,34,35]. Previously, *α*-imino esters have been utilized as substrates for umpolung reactions with several nucleophiles [36,37,38,39,40]. We also developed an asymmetric umpolung reaction of *α*-imino esters with *α*,*β*-unsaturated carbonyl compounds and haloalkanes, which provided chiral amine derivatives in high yields (Figure 1c) [41,42,43,44,45]. In this work, a formal [3 + 2] cycloaddition reaction of *α*-imino esters with azodicarboxylates was developed, which formed useful 3-aryl and 3-alkyl pentasubstituted 1,2,4-triazolines in high yields without the addition of an external oxidant (Figure 1d). The present reaction made the metal-free general preparation of 3-aryl pentasubstituted 1,2,4-triazolines under the mild condition possible for the first time.

## 2. Results and Discussion

### 2.1. Reaction Condition Optimization

The reaction conditions for the synthesis of 1,2,4-triazoline **3aa** were optimized using *α*-imino ester **1a** and diisopropyl azodicarboxylate (DIAD, **2a**) as the substrate and reactant, respectively (Table 1). Solvent screening was conducted using 1.0 equivalent of **1a** and 2.0 equivalent of **2a** in the presence of 50 mol% 1,8-diazabicyclo [5.4.0]undec-7-ene (DBU) as a base at −40 °C for 18 h. The reaction in nonpolar solvents, such as toluene, provided **3aa** in only a 9% yield, and the use of ethereal solvents and methanol resulted in poor yields (entries 1–5). Finally, the reaction in dichloromethane afforded **3aa** in a 43% yield. Next, the amount of DBU was screened, and the use of 100 and 150 mol% DBU did not increase the yield of **3aa** (entries 6 and 7). Subsequently, the effect of the reaction temperature was examined at −20 °C, 0 °C, and room temperature, and the reaction at 0 °C produced **3aa** in the highest yield of 61% (entries 8–10). The reaction was completed after 1 h (entries 11 and 12). The reaction workup procedure was changed from short column on silica gel to extraction with dichloromethane, which increased the yield of **3aa** to 88%, and **3aa** was isolated in a 72% yield (entry 13). Finally, triethylamine was employed as an inexpensive organic base; however, **3aa** was obtained in a low yield (entry 14). 

### 2.2. Substrate Scope 

**Scheme 1:**
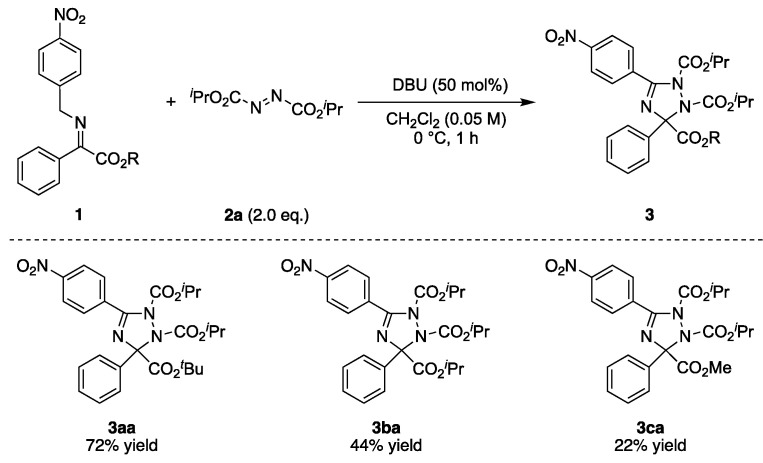
Scope of the ester moiety of **1**.

We then investigated the scope of the ester moiety in the substrate using DIAD as the reactant (Scheme 1). When the bulky *tert*-butyl ester was employed, the product **3aa** was isolated in a 72% yield, whereas the use of less bulky *iso*propyl and methyl esters resulted in 44% and 22% yields, respectively.

**Scheme 2:**
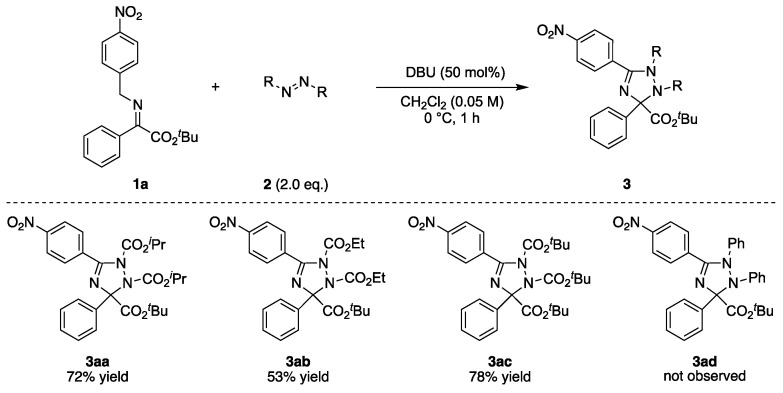
Scope of azo compounds.

Furthermore, the scope of the azo compounds was investigated using **1a** as a substrate under the optimal conditions (Scheme 2). The use of DIAD formed **3aa** in a 72% yield, and the utilization of diethyl azodicarboxylate (DEAD, **2b**) or di-*tert*-butyl azodicarboxylate (**2c**) resulted in corresponding products **3ab** and **3ac** in 53% or 78% yields, respectively. These observations indicated that increasing the bulkiness of both the substrate and reactant increases the yield of the product. The employment of azobenzene (**2d**) did not provide any cyclized product **3ad**.

**Scheme 3:**
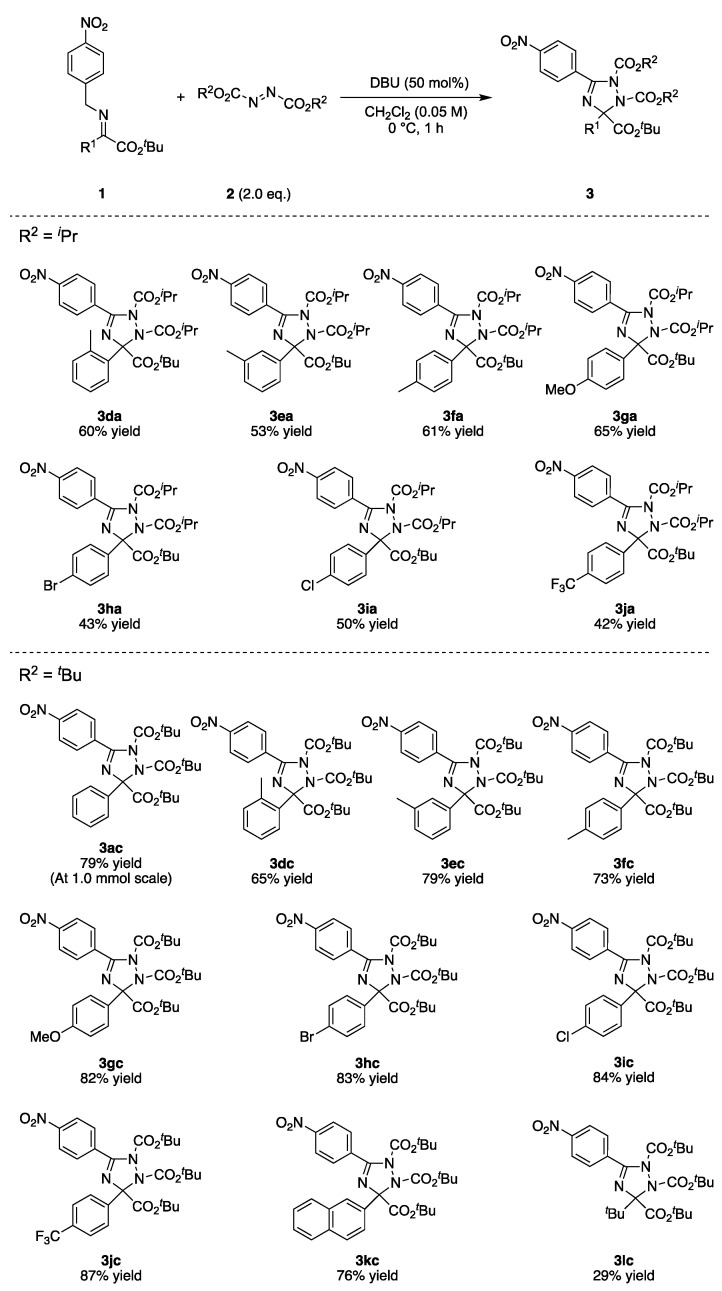
Substrate scope using **2a** and **2c** as reactants.

Subsequently, the substrate scope of the R^1^ group was determined. The substrate scope using the inexpensive **2a** and **2c** as the reactants is presented in Scheme 3. In the case of **2a** as a reactant, R^1^ groups with electron-donating substituents, such as *p*-tolyl and *p*-anisyl groups, were examined, and the products **3fa** and **3ga** were isolated in 61% and 65% yields, respectively. Substrates with *m*- and *o*-tolyl groups were well tolerated, and **3ea** and **3da** were obtained in 53% and 60% yields, respectively. Furthermore, the electron-withdrawing substituents **1h**, **1i**, and **1j** were used in the 1,2,4-triazoline synthesis, and the products were obtained in 43%, 50%, and 42% yields, respectively. The present reaction was successfully applied to several substituted substrates, and the products were obtained in moderate yields. Further substrate scope studies were conducted using the bulky azo compound **2c** as the reactant. First, the same substrates used for evaluating the substrate scope using DIAD (**2a**) were employed. The products **3dc**–**3jc** were obtained in 65–87% yields, which were higher than those obtained using DIAD as the reactant. Moreover, 2-naphthyl-substituted **1k** and *tert*-butyl-substituted **1l** were applied to the present reaction, which formed **3kc** and **3lc** in 76% and 29% yields, respectively. These results show that the present reaction is applicable to both aryl- and alkyl-substituted substrates.

**Scheme 4:**
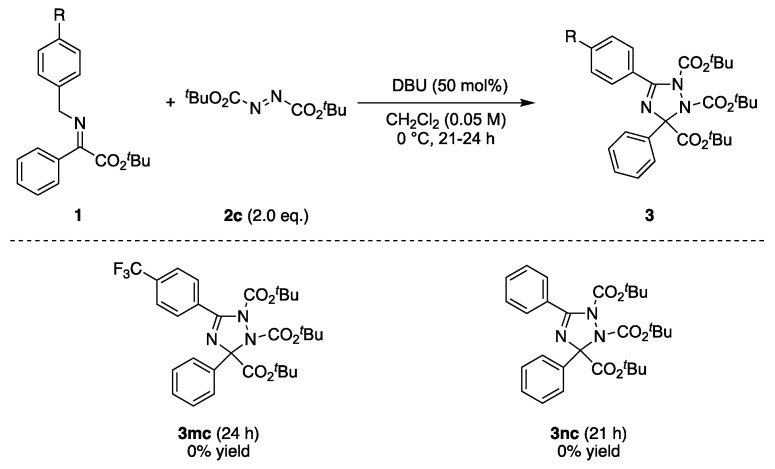
Scope of the aromatic substituent on the imine moiety.

Next, we examined the necessity for a 4-nitrobenzyl moiety on the substrate (Scheme 4). 4-Trifluoromethylbenzyl-substituted **1m** and benzyl-substituted **1n** were prepared and applied to the present reaction, which did not afford any 1,2,4-triazoline products. Only the substrate and its hydrolysis product were obtained together with the complex mixture, thereby indicating the importance of the 4-nitro group on the benzyl moiety in the production of 1,2,4-triazolines.

### 2.3. Asymmetric Synthesis

**Scheme 5:**
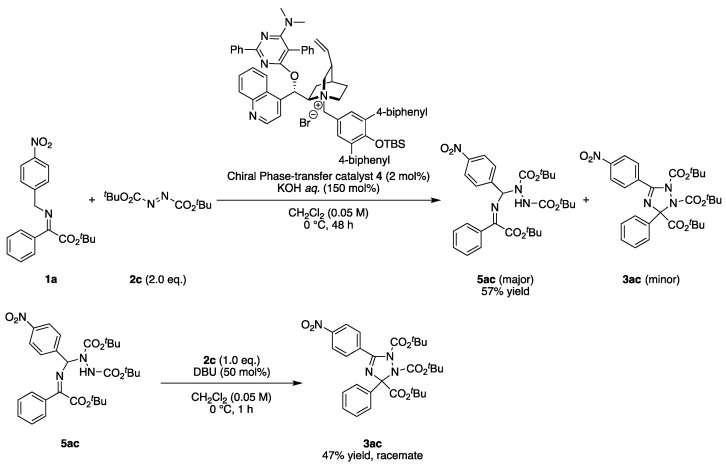
Attempt at the asymmetric synthesis of **3ac**.

The asymmetric synthesis of 1,2,4-triazolines was attempted to demonstrate the utility of this reaction (Scheme 5). Here, **1a** was reacted with **2c** in the presence of 2.0 mol% of chiral phase-transfer catalyst **4** and 150 mol% of potassium hydroxide in dichloromethane at 0 °C, which provided 1,4-addition product **5ac** in a 57% yield together with a small amount of the desired **3ac**. Notably, **5ac** was converted into 1,2,4-triazole **3ac** using a 1.0 equivalent of **2c** and 50 mol% of DBU in dichloromethane in a 47% yield. The enantiopurity of the synthesized **3ac** was evaluated via high-performance liquid chromatography using a chiral stationary phase column, and it was found to be a racemate. 

### 2.4. Reaction Mechanistic Study

**Scheme 6:**
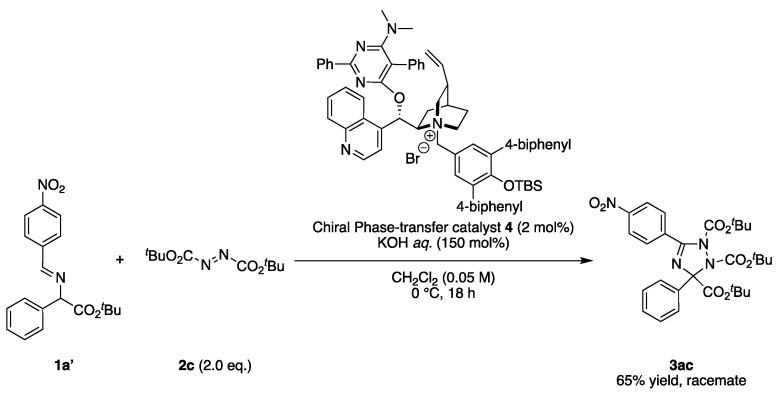
Reaction from aldimine **1a′**.

Finally, to clarify the reaction pathway, *α*-imino ester **1a** was isomerized into aldimine **1a′** because the *α*-imino ester isomerizes into aldimine under basic conditions [46]. Here, **1a′** was employed as the substrate under the same conditions as that of the asymmetric synthesis of **3ac**, which directly provided 1,2,4-triazoline **3ac** in a 65% yield and a shorter reaction time; however, **5ac** was not produced (Scheme 6). These results indicate that the reaction mechanisms for each substrate were different.

**Figure 2:**
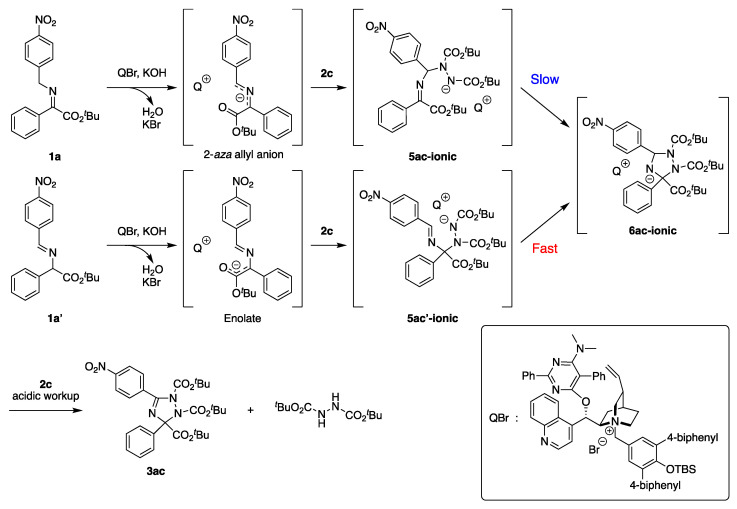
Plausible reaction mechanism.

Based on the above results, we propose a plausible reaction mechanism (Figure 2). First, the benzylic proton of substrate **1a** is deprotonated by potassium hydroxide and its counteranion is changed to the chiral ammonium salt to form a 2-aza allyl anion intermediate, which attacks the azo compounds in a 1,4-addition reaction to yield ketimine **5ac-ionic**. Finally, the cyclization of the hydrazine moiety with the imine moiety occurs, forming 1,2,4-triazolidine **6ac-ionic**, followed by the oxidation of the amine part by the additional azo compound to afford **3ac** [47]. In contrast, **1a′** reacts with potassium hydroxide to form an enolate intermediate, which is different from the reaction starting from **1a**. The as-formed intermediate then reacts with azo compound **2c** to form aldimine **5ac′-ionic**, which undergoes cyclization to form 1,2,4-triazolidine **6ac**. The reaction rate difference between **1a** and **1a′** can be explained by these plausible reaction mechanisms. In this reaction, **1a** did not isomerize into **1a′** under the reaction conditions, and the aldimine intermediate **5ac′-ionic** could be cyclized more rapidly than the ketimine intermediate **5ac-ionic** owing to its low steric hindrance around the electrophilic site. Therefore, the overall rate for the formation of **3ac** increased, and no intermediate **5ac′-ionic** was observed, even after stirring for 18 h.

## 3. Materials and Methods

^1^H- and ^13^C-NMR spectra were recorded with Bruker (Billerica, MA, USA) AVANCE III-400M (^1^H-NMR 400 MHz, ^13^C-NMR 100 MHz, and ^19^F-NMR 376 MHz). ^1^H-NMR spectra are reported as follows: chemical shift in ppm (δ) relative to the chemical shift of CHCl_3_ at 7.26 ppm or tetramethylsilane at 0 ppm, integration, multiplicities (s = singlet, d = doublet, t = triplet, q = quartet, m = multiplet), and coupling constants (Hz). ^13^C-NMR spectra are reported in ppm (δ) relative to the central line of triplet for CDCl_3_ at 77 ppm. CF_3_CO_2_H was used as an external standard for ^19^F. ESI-MS spectra were obtained with Thermo Fisher, Exactive (Waltham, MA, USA). FT-IR spectra were recorded on a JASCO FT-IR system (FT/IR-4X). HPLC analyses were performed on a JASCO HPLC system (JASCO PU 980 pump and UV-975 UV/Vis detector, Halifax, NS, Canada). Mp was measured with AS ONE ATM-02. Column chromatography on SiO_2_ and neutral SiO_2_ was performed with Kanto Silica Gel 60 (40–50 μm). All reactions were carried out under Ar atmosphere unless otherwise noted. Commercially available organic and inorganic compounds were purchased from TCI (Tokyo, Japan), Kanto Chemical Co. Inc. (Tokyo, Japan), Wako Pure Chemical Industries, Ltd. (Osaka, Japan), or Nacalai Tesque, Inc. (Kyoto, Japan), which had >95% purities, and were used without further purification. All dehydrated solvents were purchased from Wako Pure Chemical Industries, Ltd. or Nacalai Tesque, Inc., and were used without further purification.

### 3.1. Synthesis of Substrates and a Catalyst

Imine substrates **1** and **1a′** were synthesized according to the reported procedures [41,42,45]. Azo compounds were purchased from a commercial source. Chiral catalyst **4** was synthesized according to the reported procedure [42].

### 3.2. Synthesis of 1,2,4-Triazolines

#### 3.2.1. General Procedure for Table 1

A solution of **1a** (1.0 equiv) in an appropriate solvent (0.05 M) was stirred for 10 min at the reaction temperature, and **2a** (2.0 equiv) was added followed by DBU (appropriate amount). The reaction was stirred for an appropriate time at the same temperature before stopping the reaction. For the short-column procedure, the reaction mixture was directly passed through the short column (SiO_2_, ethyl acetate only) and evaporated to give the crude mixture. The NMR yield was determined by measuring its ^1^H-NMR after adding 1,3,5-trimethoxybenzene as an internal standard. For the extraction procedure, the reaction was quenched by the addition of excess amount of sat. NH_4_Cl aq. at the reaction temperature, which was extracted with CH_2_Cl_2_, dried over Na_2_SO_4_, and filtered. After the removal of solvent by evaporation, the crude product was obtained. The NMR yield was determined by measuring its ^1^H-NMR after adding 1,3,5-trimethoxybenzene as an internal standard. **3aa** was isolated through the purification by column chromatography (neutral silica gel, hexane/dichloromethane/diethylether = 7/2/1).

#### 3.2.2. General Procedure for Scheme 1, Scheme 2, Scheme 3 and Scheme 4 (Optimized Protocol)

A solution of **1** (1.0 equiv) in CH_2_Cl_2_ (0.05 M) was stirred for 10 min at 0 °C, to which **2** (2.0 equiv) was added, followed by DBU (50 mol%). The reaction was stirred for 1 h at 0 °C before quenching the reaction. The reaction was quenched by the addition of an excess amount of sat. NH_4_Cl aq. at the reaction temperature, which was extracted with CH_2_Cl_2_, dried over Na_2_SO_4_, and filtered. After the removal of solvent by evaporation, the crude product was obtained. The pure **3** was isolated through purification by column chromatography (neutral silica gel, hexane/dichloromethane/diethylether = 7/2/1).

*3-(tert-butyl) 1,2-diisopropyl 5-(4-nitrophenyl)-3-phenyl-1H-1,2,4-triazole-1,2,3(3H)-tricarboxylate* (**3aa**), White solid, 19.4 mg, 0.036 mmol, 72% yield (0.050 mmol scale reaction). m.p. 68–70 °C; ^1^H-NMR (400 MHz, CHLOROFORM-D) δ 8.26–8.30 (m, 2H), 8.00–8.04 (m, 2H), 7.68–7.72 (m, 2H), 7.34–7.43 (m, 3H), 5.10 (sep, *J* = 6.2 Hz, 1H), 4.81 (sep, *J* = 6.2 Hz, 1H), 1.40 (s, 9H), 1.39 (d, *J* = 6.2 Hz, 3H), 1.36 (d, *J* = 6.2 Hz, 3H), 1.12 (d, *J* = 6.2 Hz, 3H), 1.11 (d, *J* = 6.2 Hz, 3H); ^13^C-NMR (101 MHz, CHLOROFORM-D) δ 165.5, 156.5, 154.5, 152.2, 149.4, 137.0, 135.0, 130.8, 128.5, 127.8, 127.3, 122.9, 95.3, 83.7, 72.5, 71.4, 27.6, 22.2, 21.8, 21.53, 21.46; HRMS (ESI^+^ in MeCN) calcd. for C_27_H_33_O_8_N_4_^+^ (M + H) 541.2293 found 541.2297; IR (KBr) ν 2982, 1752, 1527, 1349, 1260, 1155, 1102, 849 cm^−1^.

*tri-isopropyl 5-(4-nitrophenyl)-3-phenyl-1H-1,2,4-triazole-1,2,3(3H)-tricarboxylate* (**3ba**), White solid, 12.8 mg, 0.024 mmol, 44% yield (0.055 mmol scale reaction). m.p. 60–62 °C; ^1^H-NMR (400 MHz, CHLOROFORM-D) δ 8.26–8.30 (m, 2H), 8.02–8.06 (m, 2H), 7.65–7.69 (m, 2H), 7.35–7.44 (m, 3H), 5.08 (sep, *J* = 6.2 Hz, 1H), 5.03 (sep, *J* = 6.2 Hz, 1H), 4.84 (sep, *J* = 6.2 Hz, 1H), 1.37 (d, *J* = 6.2 Hz, 3H), 1.33 (d, *J* = 6.2 Hz, 3H), 1.22 (d, *J* = 6.2 Hz, 3H), 1.18 (d, *J* = 6.2 Hz, 3H), 1.13 (d, *J* = 6.2 Hz, 3H), 1.12 (d, *J* = 6.2 Hz, 3H); ^13^C-NMR (101 MHz, CHLOROFORM-D) δ 166.4, 156.8, 154.2, 152.1, 149.5, 136.8, 134.7, 130.9, 128.7, 127.9, 127.2, 122.9, 94.6, 72.7, 71.4, 71.1, 22.1, 21.7, 21.52, 21.45; HRMS (ESI^+^ in MeCN) calcd. for C_26_H_31_O_8_N_4_^+^ (M + H) 527.2136 found 527.2241; IR (KBr) ν 2983, 1751, 1527, 1349, 1256, 1183, 1099, 849 cm^−1^.

*1,2-diisopropyl 3-methyl 5-(4-nitrophenyl)-3-phenyl-1H-1,2,4-triazole-1,2,3(3H)-tricarboxylate* (**3ca**), White solid, 7.6 mg, 0.016 mmol, 23% yield (0.069 mmol scale reaction). m.p. 60–62 °C; ^1^H-NMR (400 MHz, CHLOROFORM-D) δ 8.26–8.31 (m, 2H), 8.04–8.08 (m, 2H), 7.66–7.70 (m, 2H), 7.37–7.46 (m, 3H), 5.11 (sep, *J* = 6.4 Hz, 1H), 4.83 (sep, *J* = 6.4 Hz, 1H), 3.76 (s, 3H), 1.36 (d, *J* = 6.4 Hz, 3H), 1.32 (d, *J* = 6.4 Hz, 3H), 1.14 (d, *J* = 6.4 Hz, 3H), 1.13 (d, *J* = 6.4 Hz, 3H); ^13^C-NMR (101 MHz, CHLOROFORM-D) δ 167.5, 157.1, 154.2, 151.9, 149.5, 136.6, 134.4, 131.1, 128.9, 128.1, 127.1, 122.9, 94.3, 72.8, 71.6, 53.6, 22.0, 21.7, 21.54, 21.47; HRMS (ESI^+^ in MeCN) calcd. for C_24_H_27_O_8_N_4_^+^ (M + H) 499.1823 found 499.1828; IR (KBr) ν 2983, 1748, 1526, 1349, 1254, 1184, 1102, 849 cm^−1^.

*3-(tert-butyl) 1,2-diisopropyl 5-(4-nitrophenyl)-3-(o-tolyl)-1H-1,2,4-triazole-1,2,3(3H)-tricarboxylate* (**3da**), White solid, 16.6 mg, 0.299 mmol, 60% yield (0.050 mmol scale reaction). m.p. 81–83 °C; ^1^H-NMR (400 MHz, CHLOROFORM-D) δ 8.25–8.29 (m, 2H), 7.96–8.00 (m, 2H), 7.67 (d, *J* = 7.7 Hz, 1H), 7.25–7.30 (m, 2H), 7.17–7.22 (m, 1H), 5.09 (sep, *J* = 6.4 Hz, 1H), 4.86 (sep, *J* = 6.4 Hz, 1H), 2.62 (s, 3H), 1.42 (s, 9H), 1.38 (d, *J* = 6.4 Hz, 6H), 1.20 (d, *J* = 6.4 Hz, 3H), 1.19 (d, *J* = 6.4 Hz, 3H); ^13^C-NMR (101 MHz, CHLOROFORM-D) δ 165.0, 155.9, 154.5, 152.2, 149.3, 137.5, 135.0, 134.7, 131.6, 130.9, 128.7, 126.7, 125.5, 122.9, 96.9, 83.6, 72.5, 71.4, 27.5, 22.1, 21.99, 21.85, 21.64, 21.58; HRMS (ESI^+^ in MeCN) calcd. for C_28_H_35_O_8_N_4_^+^ (M + H) 555.2449 found 555.2449; IR (KBr) ν 2982, 1744, 1527, 1349, 1257, 1157, 1103, 849 cm^−1^.

*3-(tert-butyl) 1,2-diisopropyl 5-(4-nitrophenyl)-3-(m-tolyl)-1H-1,2,4-triazole-1,2,3(3H)-tricarboxylate* (**3ea**), White solid, 14.8 mg, 0.027 mmol, 53% yield (0.050 mmol scale reaction). m.p. 96–98 °C; ^1^H-NMR (400 MHz, CHLOROFORM-D) δ 8.26–8.30 (m, 2H), 8.04–8.00 (m, 2H), 7.50 (s, 1H), 7.48 (d, *J* = 6.8 Hz, 1H), 7.28–7.32 (m, 1H), 7.18 (d, *J* = 7.8 Hz, 1H), 5.11 (sep, *J* = 6.3 Hz, 1H), 4.81 (sep, *J* = 6.3 Hz, 1H), 2.40 (s, 3H), 1.40 (s, 9H), 1.39 (d, *J* = 6.3 Hz, 3H), 1.36 (d, *J* = 6.3 Hz, 3H), 1.14 (d, *J* = 6.3 Hz, 6H); ^13^C-NMR (101 MHz, CHLOROFORM-D) δ 165.5, 156.3, 154.5, 152.2, 149.4, 137.4, 136.9, 135.0, 130.8, 129.3, 127.93, 127.81, 124.5, 122.9, 95.4, 83.6, 72.6, 71.3, 27.6, 22.2, 21.8, 21.59, 21.56, 21.48; HRMS (ESI^+^ in MeCN) calcd. for C_28_H_35_O_8_N_4_^+^ (M + H) 555.2449 found 555.2454; IR (KBr) ν 2981, 1747, 1526, 1348, 1253, 1155, 1103, 845 cm^−1^.

*3-(tert-butyl) 1,2-diisopropyl 5-(4-nitrophenyl)-3-(p-tolyl)-1H-1,2,4-triazole-1,2,3(3H)-tricarboxylate* (**3fa**), White solid, 16.8 mg, 0.030 mmol, 61% yield (0.050 mmol scale reaction). m.p. 76–78 °C; ^1^H-NMR (400 MHz, CHLOROFORM-D) δ 8.26–8.30 (m, 2H), 7.99–8.03 (m, 2H), 7.55–7.59 (m, 2H), 7.21 (d, *J* = 8.0 Hz, 2H), 5.09 (sep, *J* = 6.3 Hz, 1H), 4.81 (sep, *J* = 6.3 Hz, 1H), 2.37 (s, 3H), 1.40 (s, 9H), 1.39 (d, *J* = 6.4 Hz, 3H), 1.35 (d, *J* = 6.4 Hz, 3H), 1.13 (d, *J* = 6.4 Hz, 3H), 1.20 (d, *J* = 6.4 Hz, 3H); ^13^C-NMR (101 MHz, CHLOROFORM-D) δ 165.6, 156.3, 154.5, 152.2, 149.3, 138.4, 135.1, 134.0, 130.7, 128.6, 127.2, 122.9, 95.3, 83.6, 72.6, 71.3, 27.6, 22.2, 21.8, 21.56, 21.45, 21.1; HRMS (ESI^+^ in MeCN) calcd. for C_28_H_35_O_8_N_4_^+^ (M + H) 555.2449 found 555.2455; IR (KBr) ν 2982, 1747, 1526, 1348, 1258, 1155, 1102, 849 cm^−1^.

*3-(tert-butyl) 1,2-diisopropyl 3-(4-methoxyphenyl)-5-(4-nitrophenyl)-1H-1,2,4-triazole-1,2,3(3H)-tricarboxylate* (**3ga**), White solid, 18.6 mg, 0.033 mmol, 65% yield (0.050 mmol scale reaction). m.p. 98–100 °C; ^1^H-NMR (400 MHz, CHLOROFORM-D) δ 8.26–8.30 (m, 2H), 8.00–8.04 (m, 2H), 7.59–7.63 (m, 2H), 6.91–6.95 (m, 2H), 5.09 (sep, *J* = 6.4 Hz, 1H), 4.81 (sep, *J* = 6.4 Hz, 1H), 3.83 (s, 3H), 1.40 (s, 9H), 1.39 (d, *J* = 6.4 Hz, 3H), 1.35 (d, *J* = 6.4 Hz, 3H), 1.13 (d, *J* = 6.4 Hz, 3H), 1.10 (d, *J* = 6.4 Hz, 3H); ^13^C-NMR (101 MHz, CHLOROFORM-D) δ 165.7, 159.7, 156.3, 154.5, 152.2, 149.3, 135.1, 130.7, 129.1, 128.6, 122.9, 113.2, 95.0, 83.6, 72.6, 71.3, 55.2, 27.6, 22.2, 21.8, 21.56, 21.44; HRMS (ESI^+^ in MeCN) calcd. for C_28_H_35_O_9_N_4_^+^ (M + H) 571.2399 found 571.2404; IR (KBr) ν 2980, 1757, 1526, 1348, 1253, 1155, 1102, 849 cm^−1^.

*3-(tert-butyl) 1,2-diisopropyl 3-(4-bromophenyl)-5-(4-nitrophenyl)-1H-1,2,4-triazole-1,2,3(3H)-tricarboxylate* (**3ha**), White solid, 13.3 mg, 0.215 mmol, 43% yield (0.050 mmol scale reaction). m.p. 156–158 °C; ^1^H-NMR (400 MHz, CHLOROFORM-D) δ 8.27–8.31 (m, 2H), 7.99–8.03 (m, 2H), 7.51–7.59 (m, 4H), 5.10 (sep, *J* = 6.3 Hz, 1H), 4.82 (sep, *J* = 6.3 Hz, 1H), 1.40 (s, 9H), 1.39 (d, *J* = 6.3 Hz, 3H), 1.36 (d, *J* = 6.3 Hz, 3H), 1.13 (d, *J* = 6.3 Hz, 3H), 1.11 (d, *J* = 6.3 Hz, 3H); ^13^C-NMR (101 MHz, CHLOROFORM-D) δ 165.0, 156.7, 154.5, 152.0, 149.5, 136.3, 134.8, 131.0, 130.8, 129.1, 122.99, 122.00, 94.8, 84.1, 72.8, 71.6, 27.6, 22.2, 21.8, 21.56, 21.42; HRMS (ESI^+^ in MeCN) calcd. for C_27_H_32_O_8_N_4_Br^+^ (M + H) 619.1398 found 619.1402; IR (KBr) ν 2981, 1751, 1526, 1348, 1257, 1155, 1102, 849 cm^−1^.

*3-(tert-butyl) 1,2-diisopropyl 3-(4-chlorophenyl)-5-(4-nitrophenyl)-1H-1,2,4-triazole-1,2,3(3H)-tricarboxylate* (**3ia**), White solid, 14.4 mg, 0.025 mmol, 50% yield (0.050 mmol scale reaction). m.p. 154–156 °C; ^1^H-NMR (400 MHz, CHLOROFORM-D) δ 8.27–8.31 (m, 2H), 7.99–8.03 (m, 2H), 7.61–7.65 (m, 2H), 7.35–7.40 (m, 2H), 5.10 (sep, *J* = 6.4 Hz, 1H), 4.82 (sep, *J* = 6.4 Hz, 1H), 1.40 (s, 9H), 1.39 (d, *J* = 6.4 Hz, 3H), 1.35 (d, *J* = 6.4 Hz, 3H), 1.13 (d, *J* = 6.4 Hz, 3H), 1.11 (d, *J* = 6.4 Hz, 3H); ^13^C-NMR (101 MHz, CHLOROFORM-D) δ 165.2, 156.7, 154.5, 152.0, 149.4, 135.7, 134.8, 134.5, 130.8, 128.8, 128.0, 123.0, 94.8, 84.1, 72.8, 71.6, 27.6, 22.2, 21.8, 21.54, 21.42; HRMS (ESI^+^ in MeCN) calcd. for C_27_H_32_O_8_N_4_Cl^+^ (M + H) 575.1903 found 575.1910; IR (KBr) ν 2981, 1751, 1527, 1351, 1259, 1155, 1102, 849 cm^−1^.

*3-(tert-butyl) 1,2-diisopropyl 5-(4-nitrophenyl)-3-(4-(trifluoromethyl)phenyl)-1H-1,2,4-triazole-1,2,3(3H)-tricarboxylate* (**3ja**), White solid, 12.7 mg, 0.021mmol, 42% yield (0.050 mmol scale reaction). m.p. 77–79 °C; ^1^H-NMR (400 MHz, CHLOROFORM-D) δ 8.27–8.32 (m, 2H), 8.00–8.04 (m, 2H), 7.83 (d, *J* = 8.0 Hz, 2H), 7.67 (d, *J* = 8.3 Hz, 2H), 5.12 (sep, *J* = 6.3 Hz, 1H), 4.82 (sep, *J* = 6.3 Hz, 1H), 1.40 (s, 9H), 1.39 (d, *J* = 6.3 Hz, 3H), 1.37 (d, *J* = 6.3 Hz, 3H), 1.13 (d, *J* = 6.3 Hz, 3H), 1.10 (d, *J* = 6.3 Hz, 3H); ^13^C-NMR (101 MHz, CHLOROFORM-D) δ 165.0, 157.0, 154.5, 151.9, 149.5, 141.1, 134.6, 130.86, 130.71 (q, *J* = 32.3 Hz), 127.8, 124.8 (q, *J* = 3.9 Hz), 123.9 (q, *J* = 272.8 Hz), 123.0, 94.8, 84.3, 72.9, 71.7, 27.6, 22.2, 21.8, 21.54, 21.40; ^19^F-NMR (376 MHz, CHLOROFORM-D) δ -62.5; HRMS (ESI^+^ in MeCN) calcd. for C_28_H_32_O_8_N_4_F_3_^+^ (M + H) 609.2167 found 609.2172; IR (KBr) ν 2983, 1752,1528,1326, 1257, 1165, 1102, 850 cm^−1^.

*3-(tert-butyl) 1,2-diethyl 5-(4-nitrophenyl)-3-phenyl-1H-1,2,4-triazole-1,2,3(3H)-tricarboxylate* (**3cb**), White solid, 13.6 mg, 0.027 mmol, 53% yield (0.050 mmol scale reaction). m.p. 67–69 °C; ^1^H-NMR (400 MHz, CHLOROFORM-D) δ 8.26–8.30 (m, 2H), 8.01–8.05 (m, 2H), 7.68–7.72 (m, 2H), 7.35–7.45 (m, 3H), 4.38–4.46 (m, 1H), 4.20–4.30 (m, 1H), 4.05–4.18 (m, 2H), 1.40 (s, 9H), 1.36 (t, *J* = 7.1 Hz, 3H), 1.10 (t, *J* = 7.1 Hz, 3H); ^13^C-NMR (101 MHz, CHLOROFORM-D) δ 165.4, 156.2, 154.9, 152.4, 149.4, 136.8, 134.8, 130.8, 128.6, 127.9, 127.3, 123.0, 95.5, 83.9, 64.1, 63.1, 27.6, 14.4, 13.8; HRMS (ESI^+^ in MeCN) calcd. for C_25_H_29_O_8_N_4_^+^ (M + H) 513.1980 found 513.1984; IR (KBr) ν 2980, 1752, 1526, 1351, 1258, 1153, 1022, 845 cm^−1^.

*tri-tert-butyl 5-(4-nitrophenyl)-3-phenyl-1H-1,2,4-triazole-1,2,3(3H)-tricarboxylate* (**3ac**), White solid, 22.4 mg, 0.039 mmol, 78% yield (0.050 mmol scale reaction). Large-scale synthesis was conducted using 1.0 mmol (340.4 mg) of **1a**, and 0.79 mmol (447.5 mg, 79% yield) of **3ac** was isolated. m.p. 87–89 °C; ^1^H-NMR (400 MHz, CHLOROFORM-D) δ 8.26–8.30 (m, 2H), 7.99–8.03 (m, 2H), 7.68–7.71 (m, 2H), 7.33–7.44 (m, 3H), 1.58 (s, 9H), 1.41 (s, 9H), 1.29 (s, 9H); ^13^C-NMR (101 MHz, CHLOROFORM-D) δ 165.9, 156.6, 153.5, 151.0, 149.3, 137.3, 135.4, 130.7, 128.4, 127.8, 127.3, 122.9, 94.8, 84.8, 83.6, 83.0, 28.2, 27.6 (1 peak is overlapped with the other peak); HRMS (ESI^+^ in MeCN) calcd. for C_29_H_37_O_8_N_4_^+^ (M + H) 569.2606 found 569.2615; IR (KBr) ν 2979, 1744, 1527, 1369, 1349, 1253, 1149, 849 cm^−1^; HPLC (CHIRALPAK AD-H column, hexane/2-propanol = 95/5, flow rate 1.0 mL/min, 25 °C, 254 nm) first peak: t_R_ = 5.8 min and second peak: t_R_ = 6.7 min.

*tri-tert-butyl 5-(4-nitrophenyl)-3-(o-tolyl)-1H-1,2,4-triazole-1,2,3(3H)-tricarboxylate* (**3dc**), White solid, 19.0 mg, 0.033 mmol, 65% yield (0.050 mmol scale reaction). m.p. 99–101 °C; ^1^H-NMR (400 MHz, CHLOROFORM-D) δ 8.25–8.29 (m, 2H), 7.94–7.98 (m, 2H), 7.70 (d, *J* = 7.5 Hz, 1H), 7.19–7.29 (m, 3H), 2.62 (s, 3H), 1.58 (s, 9H), 1.42 (s, 9H), 1.36 (s, 9H); ^13^C-NMR (101 MHz, CHLOROFORM-D) δ 165.5, 156.0, 153.7, 151.0, 149.2, 137.5, 135.4, 135.0, 131.6, 130.8, 128.6, 126.6, 125.5, 122.9, 96.4, 84.8, 83.6, 83.0, 28.2, 27.7, 27.5, 22.0; HRMS (ESI^+^ in MeCN) calcd. for C_30_H_39_O_8_N_4_^+^ (M + H) 583.2762 found 583.2767; IR (KBr) ν 2979, 1742, 1527, 1369, 1348, 1254, 1150, 849 cm^−1^.

*tri-tert-butyl 5-(4-nitrophenyl)-3-(m-tolyl)-1H-1,2,4-triazole-1,2,3(3H)-tricarboxylate* (**3ec**), White solid, 23.0 mg, 0.039 mmol, 79% yield (0.050 mmol scale reaction). m.p. 76–78 °C; ^1^H-NMR (400 MHz, CHLOROFORM-D) δ 8.26–8.30 (m, 2H), 7.99–8.03 (m, 2H), 7.50 (s, 1H), 7.49 (d, *J* = 7.7 Hz, 1H), 7.28–7.33 (m, 1H), 7.17 (d, *J* = 7.8 Hz, 1H), 2.41 (s, 3H), 1.58 (s, 9H), 1.42 (s, 9H), 1.31 (s, 9H); ^13^C-NMR (101 MHz, CHLOROFORM-D) δ 165.9, 156.5, 153.5, 151.0, 149.2, 137.3, 137.1, 135.4, 130.7, 129.2, 127.9, 127.7, 124.5, 122.9, 94.9, 84.7, 83.5, 82.9, 28.2, 27.6, 21.6 (1 peak is overlapped with the other peak); HRMS (ESI^+^ in MeCN) calcd. for C_30_H_39_O_8_N_4_^+^ (M + H) 583.2762 found 583.2770; IR (KBr) ν 2979, 1743, 1526, 1369, 1348, 1257, 1149, 851 cm^−1^.

*tri-tert-butyl 5-(4-nitrophenyl)-3-(p-tolyl)-1H-1,2,4-triazole-1,2,3(3H)-tricarboxylate* (**3fc**), White solid, 21.4 mg, 0.0367 mmol, 73% yield (0.050 mmol scale reaction). m.p. 96–98 °C;^1^H-NMR (400 MHz, CHLOROFORM-D) δ 8.25–8.30 (m, 2H), 7.98–8.02 (m, 2H), 7.56–7.59 (m, 2H), 7.22 (d, *J* = 8.0 Hz, 2H), 2.38 (s, 3H), 1.57 (s, 9H), 1.41 (s, 9H), 1.29 (s, 9H); ^13^C-NMR (101 MHz, CHLOROFORM-D) δ 166.0, 156.5, 153.4, 151.0, 149.2, 138.2, 135.5, 134.3, 130.7, 128.5, 127.2, 122.9, 94.8, 84.7, 83.5, 82.9, 28.2, 27.6, 21.1 (1 peak is overlapped with the other peak); HRMS (ESI^+^ in MeCN) calcd. for C_30_H_39_O_8_N_4_^+^ (M + H) 583.2762 found 583.2767; IR (KBr) ν 2979, 1744, 1527, 1369, 1348, 1254, 1150, 850 cm^−1^.

*tri-tert-butyl 3-(4-methoxyphenyl)-5-(4-nitrophenyl)-1H-1,2,4-triazole-1,2,3(3H)-tricarboxylate* (**3gc**), White solid, 24.4 mg, 0.041 mmol, 82% yield (0.050 mmol scale reaction). m.p. 87–89 °C; ^1^H-NMR (400 MHz, CHLOROFORM-D) δ 8.26–8.30 (m, 2H), 7.98–8.02 (m, 2H), 7.59–7.64 (m, 2H), 6.91–6.96 (m, 2H), 3.83 (s, 3H), 1.58 (s, 9H), 1.41 (s, 9H), 1.29 (s, 9H); ^13^C-NMR (101 MHz, CHLOROFORM-D) δ 166.1, 159.6, 156.5, 153.4, 151.0, 149.2, 135.5, 130.6, 129.4, 128.6, 122.9, 113.2, 94.5, 84.7, 83.5, 82.9, 55.2, 28.2, 27.6 (1 peak is overlapped with the other peak); HRMS (ESI^+^ in MeCN) calcd. for C_30_H_39_O_9_N_4_^+^ (M + H) 599.2712 found 599.2715; IR (KBr) ν 2979, 1744, 1527, 1369, 1348, 1253, 1150, 849 cm^−1^.

*tri-tert-butyl 3-(4-bromophenyl)-5-(4-nitrophenyl)-1H-1,2,4-triazole-1,2,3(3H)-tricarboxylate* (**3hc**), White solid, 27.0 mg, 0.042 mmol, 83% yield (0.050 mmol scale reaction). m.p. 94–96 °C; ^1^H-NMR (400 MHz, CHLOROFORM-D) δ 8.26–8.30 (m, 2H), 7.97–8.00 (m, 2H), 7.52–7.60 (m, 4H), 1.58 (s, 9H), 1.41 (s, 9H), 1.29 (s, 9H); ^13^C-NMR (101 MHz, CHLOROFORM-D) δ 165.6, 157.0, 153.4, 150.8, 149.3, 136.6, 135.2, 130.9, 130.7, 129.1, 123.0, 122.7, 94.2, 85.1, 84.2, 83.2, 28.2, 27.6 (1 peak is overlapped with the other peak); HRMS (ESI^+^ in MeCN) calcd. for C_29_H_36_O_8_N_4_Br^+^ (M + H) 647.1711 found 647.1721; IR (KBr) ν 2979, 1751, 1527, 1369, 1348, 1253, 1149, 849 cm^−1^.

*tri-tert-butyl 3-(4-chlorophenyl)-5-(4-nitrophenyl)-1H-1,2,4-triazole-1,2,3(3H)-tricarboxylate* (**3ic**), White solid, 25.4 mg, 0.042 mmol, 84% yield (0.050 mmol scale reaction). m.p. 86–88 °C; ^1^H-NMR (400 MHz, CHLOROFORM-D) δ 8.26–8.31 (m, 2H), 7.97–8.02 (m, 2H), 7.62–7.66 (m, 2H), 7.36–7.40 (m, 2H), 1.58 (s, 9H), 1.41 (s, 9H), 1.28 (s, 9H); ^13^C-NMR (101 MHz, CHLOROFORM-D) δ 165.6, 156.9, 153.4, 150.8, 149.3, 136.0, 135.2, 134.4, 130.7, 128.8, 128.0, 123.0, 94.3, 85.0, 84.0, 83.2, 28.2, 27.6 (1 peak is overlapped with the other peak); HRMS (ESI^+^ in MeCN) calcd. for C_29_H_36_O_8_N_4_Cl^+^ (M + H) 603.2216 found 603.2227; IR (KBr) ν 2979, 1752, 1527,1369, 1348, 1255, 1149, 848 cm^−1^.

*tri-tert-butyl 5-(4-nitrophenyl)-3-(4-(trifluoromethyl)phenyl)-1H-1,2,4-triazole-1,2,3(3H)-tricarboxylate* (**3jc**), White solid, 27.7 mg, 0.044 mmol, 87% yield (0.050 mmol scale reaction). m.p. 106–108 °C; ^1^H-NMR (400 MHz, CHLOROFORM-D) δ 8.27–8.31 (m, 2H), 7.98–8.02 (m, 2H), 7.84 (d, *J* = 8.2 Hz, 2H), 7.67 (d, *J* = 8.2 Hz, 2H), 1.59 (s, 9H), 1.41 (s, 9H), 1.28 (s, 9H); ^13^C-NMR (101 MHz, CHLOROFORM-D) δ 165.5, 157.1, 153.5, 150.7, 149.4, 141.4, 135.1, 130.8, 130.5 (q, *J* = 33.1 Hz), 127.8, 124.8 (q, *J* = 3.7 Hz), 124.0 (q, *J* = 273.1 Hz), 123.0, 94.3, 85.1, 84.2, 83.4, 28.2, 27.6 (1 peak is overlapped with the other peak); ^19^F-NMR (376 MHz, CHLOROFORM-D) δ -62.5; HRMS (ESI^+^ in MeCN) calcd. for C_30_H_36_O_8_N_4_F_3_^+^ (M + H) 637.2480 found 637.2484; IR (KBr) ν 2980, 1751, 1528, 1370, 1326, 1253, 1149, 850 cm^−1^.

*tri-tert-butyl 3-(naphthalen-2-yl)-5-(4-nitrophenyl)-1H-1,2,4-triazole-1,2,3(3H)-tricarboxylate* (**3kc**), White solid, 23.6 mg, 0.038 mmol, 76% yield (0.050 mmol scale reaction). m.p. 108–110 °C; ^1^H-NMR (400 MHz, CHLOROFORM-D) δ 8.26–8.30 (m, 2H), 8.13 (s, 1H), 8.00–8.04 (m, 2H), 7.83–7.91 (m, 4H), 7.46–7.53 (m, 2H), 1.61 (s, 9H), 1.43 (s, 9H), 1.31 (s, 9H); ^13^C-NMR (101 MHz, CHLOROFORM-D) δ 166.0, 156.8, 153.6, 151.0, 149.3, 135.3, 134.83 133.3, 132.7, 130.8, 128.4, 127.6, 127.3, 126.4, 126.02, 125.96, 125.7, 123.0, 95.0, 84.8, 83.9, 83.1, 28.2, 27.7 (1 peak is overlapped with the other peak); HRMS (ESI^+^ in MeCN) calcd. for C_33_H_39_O_8_N_4_^+^ (M + H) 619.2762 found 619.2767; IR (KBr) ν 2979, 1746, 1526, 1369, 1348, 1252, 1149, 851 cm^−1^.

*tri-tert-butyl 3-(tert-butyl)-5-(4-nitrophenyl)-1H-1,2,4-triazole-1,2,3(3H)-tricarboxylate* (**3lc**), White solid, 7.92 mg, 0.014 mmol, 29% yield (0.050 mmol scale reaction). m.p. 68–70 °C; ^1^H-NMR (400 MHz, CHLOROFORM-D) δ 8.27–8.31 (m, 2H), 7.91–7.95 (m, 2H), 1.56 (s, 9H), 1.40 (s, 9H), 1.30 (s, 9H), 1.19 (s, 9H); ^13^C-NMR (101 MHz, CHLOROFORM-D) δ 164.8, 155.9, 154.6, 150.7, 149.0, 136.4, 130.0, 123.0, 98.7, 84.3, 82.8, 82.6, 39.1, 28.1, 27.76, 27.62, 25.2; HRMS (ESI^+^ in MeCN) calcd. For C_27_H_41_O_8_N_4_^+^ (M + H) 549.2919 found 549.2920; IR (KBr) ν 2979, 1758, 1528, 1370, 1348, 1255, 1149, 850 cm^−1^.

#### 3.2.3. General Procedure for Scheme 5 (for the Synthesis of **5ac**)

A solution of **1a** (1.0 equiv) and **4** (2 mol%) in CH_2_Cl_2_ (0.05 M) was stirred for 10 min at 0 °C, to which **2c** (2.0 equiv) was added, followed by potassium hydroxyde (50% *aq.*, 150 mol%). The reaction was stirred for 48 h at 0 °C before quenching the reaction. The reaction was quenched by the addition of an excess amount of *sat.* NH_4_Cl *aq.* at the reaction temperature, which was extracted with CH_2_Cl_2_, dried over Na_2_SO_4_, and filtered. After the removal of solvent by evaporation, the crude product was obtained. The pure **5ac** was isolated through purification by column chromatography (neutral silica gel, hexane/dichloromethane/diethylether = 7/2/1) in 53% yield.

*di-tert-butyl (Z)-1-(((2-(tert-butoxy)-2-oxo-1-phenylethylidene)amino)(4-nitrophenyl)methyl)hydrazine-1,2-dicarboxylate* (**5ac**), White solid, 30.3 mg, 0.053 mmol, 53% yield (0.10 mmol scale reaction). m.p. 85–87 °C: ^1^H-NMR (400 MHz, CHLOROFORM-D) δ 8.15 (d, *J* = 8.8 Hz, 2H), 7.84–7.86 (m, 2H), 7.64 (d, *J* = 8.5 Hz, 2H), 7.49–7.53 (m, 1H), 7.42–7.46 (m, 2H), 6.88 (br, 1H), 6.50 (br, 1H), 1.48 (s, 9H), 1.46 (s, 9H), 1.31 (s, 9H); ^13^C-NMR (101 MHz, CHLOROFORM-D) δ 163.4, 162.2, 154.4, 147.8, 145.4, 133.9, 131.8, 128.9, 128.6, 127.9, 123.0, 84.9, 82.4, 80.9, 28.2, 28.08, 28.01 (2 peaks are overlapped with the other peaks); HRMS (ESI^+^ in MeCN) calcd. for C_29_H_39_O_8_N_4_^+^ (M + H) 571.2762 found 571.2761; IR (KBr) ν 2979, 1727, 1525, 1368, 1346, 1259, 1153, 854 cm^−1^.

#### 3.2.4. General Procedure for Scheme 5 (for the Synthesis of **3ac**)

A solution of **5ac** (1.0 equiv) in CH_2_Cl_2_ (0.05 M) was stirred for 10 min at 0 °C, which **2c** (1.0 equiv) was added, followed by DBU (50 mol%). The reaction was stirred for 1 h at 0 °C before quenching the reaction. The reaction was quenched by the addition of an excess amount of *sat.* NH_4_Cl *aq.* at the reaction temperature, which was extracted with CH_2_Cl_2_, dried over Na_2_SO_4_, and filtered. After the removal of solvent by evaporation, the crude product was obtained. The pure **3ac** was isolated through the purification by column chromatography (neutral silica gel, hexane/dichloromethane/diethylether = 7/2/1) in a 47% yield as a racemate.

Enantiomeric excess was determined by HPLC (CHIRALPAK AD-H, hexane/2-propanol = 95/5, flow rate 1.0 mL/min, 25 °C, 254 nm): first peak: t_R_ = 5.8 min and second peak: t_R_ = 6.8 min.

#### 3.2.5. General Procedure for Scheme 6

A solution of **1a′** (1.0 equiv) and **4** (2 mol%) in CH_2_Cl_2_ (0.05 M) was stirred for 10 min at 0 °C, to which **2c** (2.0 equiv) was added, followed by potassium hydroxyde (50% *aq.*, 150 mol%). The reaction was stirred for 18 h at 0 °C before quenching the reaction. The reaction was quenched by the addition of an excess amount of *sat.* NH_4_Cl *aq.* at the reaction temperature, which was extracted with CH_2_Cl_2_, dried over Na_2_SO_4_, and filtered. After the removal of solvent by evaporation, the crude product was obtained. The pure **3ac** was isolated through the purification by column chromatography (neutral silica gel, hexane/dichloromethane/diethylether = 7/2/1) in a 65% yield as a racemate.

Enantiomeric excess was determined by HPLC (CHIRALPAK AD-H, hexane/2-propanol = 95/5, flow rate 1.0 mL/min, 25 °C, 254 nm): first peak: t_R_ = 5.8 min and second peak: t_R_ = 6.8 min.

## 4. Conclusions

This study developed a direct synthesis method of 1,2,4-triazolines from easily accessible *α*-imino esters using commercial azo compounds under DBU catalysis, which provided excellent product yields. The study on the substrate scope revealed that the present reaction could be applied to a wide range of substrates and reactants, irrespective of their steric and electronic characteristics. The present reaction is the first general method for the metal-free preparation of 3-aryl pentasubstituted 1,2,4-triazolines under the mild condition. The reaction mechanism suggests that the *α*-imino ester reacts through the 2-aza allyl anion intermediate. However, its isomerized aldimine reacts with the enolate intermediate. We are further investigating the application of these products in the preparation of useful molecules.

## Figures and Tables

**Table 1:** Reaction condition optimization.

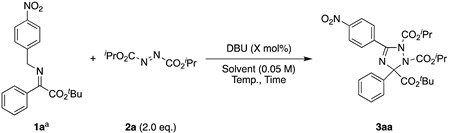					
Entry	Solvent	X	Temp. (°C)	Time (h)	Yield (%) ^b^
1	toluene	50	−40	18	9
2	Et_2_O	50	−40	18	4
3	CH_2_Cl_2_	50	−40	18	43
4	tetrahydrofuran	50	−40	18	28
5	MeOH	50	−40	18	0
6	CH_2_Cl_2_	100	−40	18	43
7	CH_2_Cl_2_	150	−40	18	42
8	CH_2_Cl_2_	50	−20	18	53
9	CH_2_Cl_2_	50	0	18	61
10	CH_2_Cl_2_	50	r.t	18	51
11	CH_2_Cl_2_	50	0	1	64
12	CH_2_Cl_2_	50	0	53	64
13 ^c^	CH_2_Cl_2_	50	0	1	88 (72 ^d^)
14 ^c,e^	CH_2_Cl_2_	50	0	1	10

^a^ 0.05 mmol scale reaction. ^b^ All yields were obtained via ^1^H-NMR analysis using 1,3,5-trimethoxybenzene as an internal standard. ^c^ Workup method was changed from short column to extraction. ^d^ Isolated yield. ^e^ Triethylamine was employed instead of DBU as a base.

## Data Availability

Data are included in the manuscript or Supplementary Data File. All the known compounds were prepared according to previously reported procedures.

## Supplementary Materials

The following supporting information can be downloaded at: https://www.mdpi.com/article/10.3390/molecules28114339/s1, It contains ^1^H, ^13^C, and ^19^F NMR charts and HPLC spectra of the products and intermediates.

## Sample Availability

Samples of the compounds are not available from the authors.
