# Supplementary material for: Formal [3 + 2] Cycloaddition of α-Imino Esters with Azo Compounds: Facile Construction of Pentasubstituted 1,2,4-Triazoline Skeletons
Source: Molecules. 2023 May 25;28(11):4339. doi: 10.3390/molecules28114339 (PMC10254835; doi:10.3390/molecules28114339)
Supplement: Supplementary file 1 [file molecules-28-04339-s001.zip › molecules-2424544-supplementary.pdf]

^{1}H, ^{13}C, and ^{19}F-NMR spectra and HPLC charts.

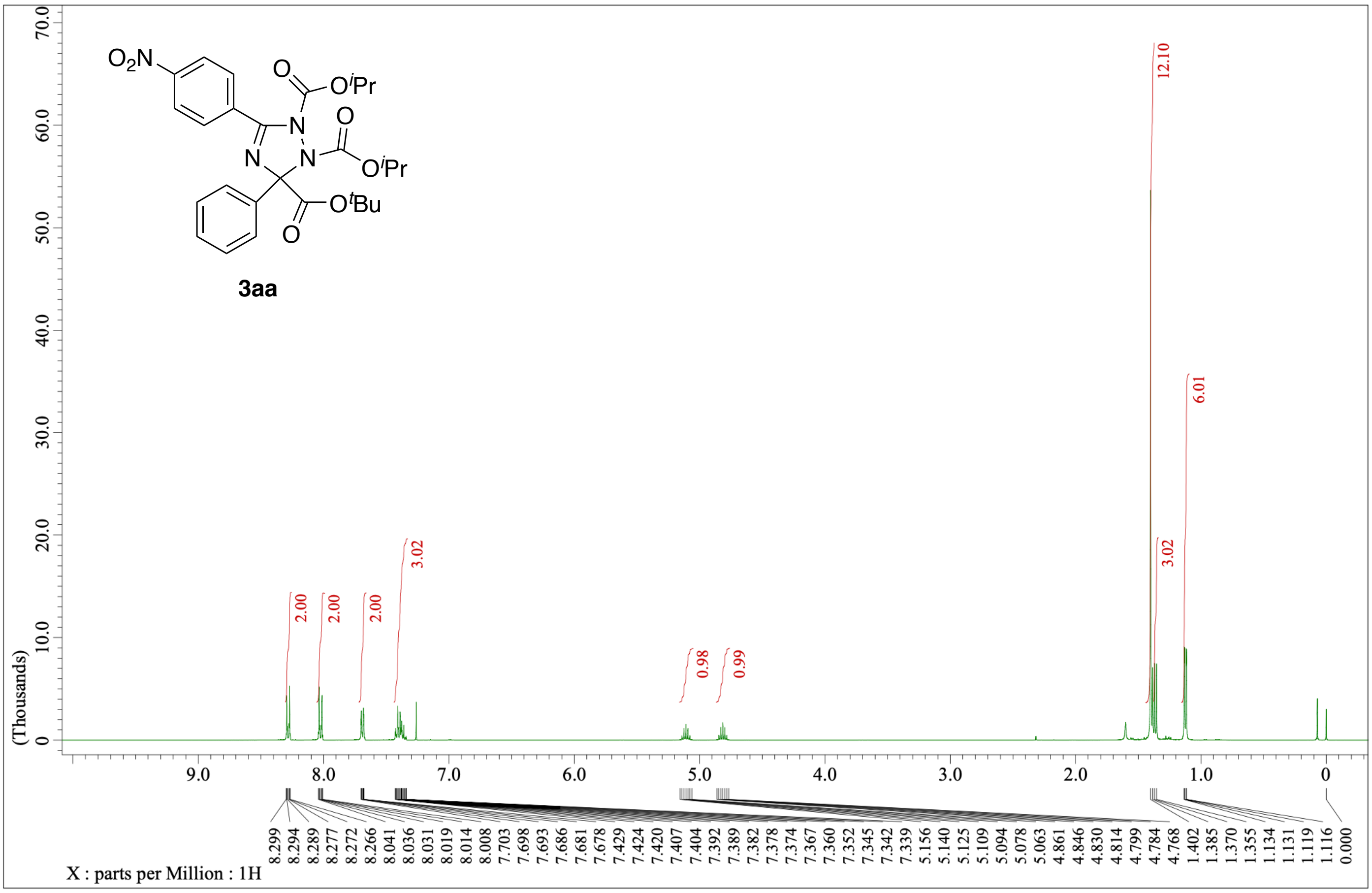

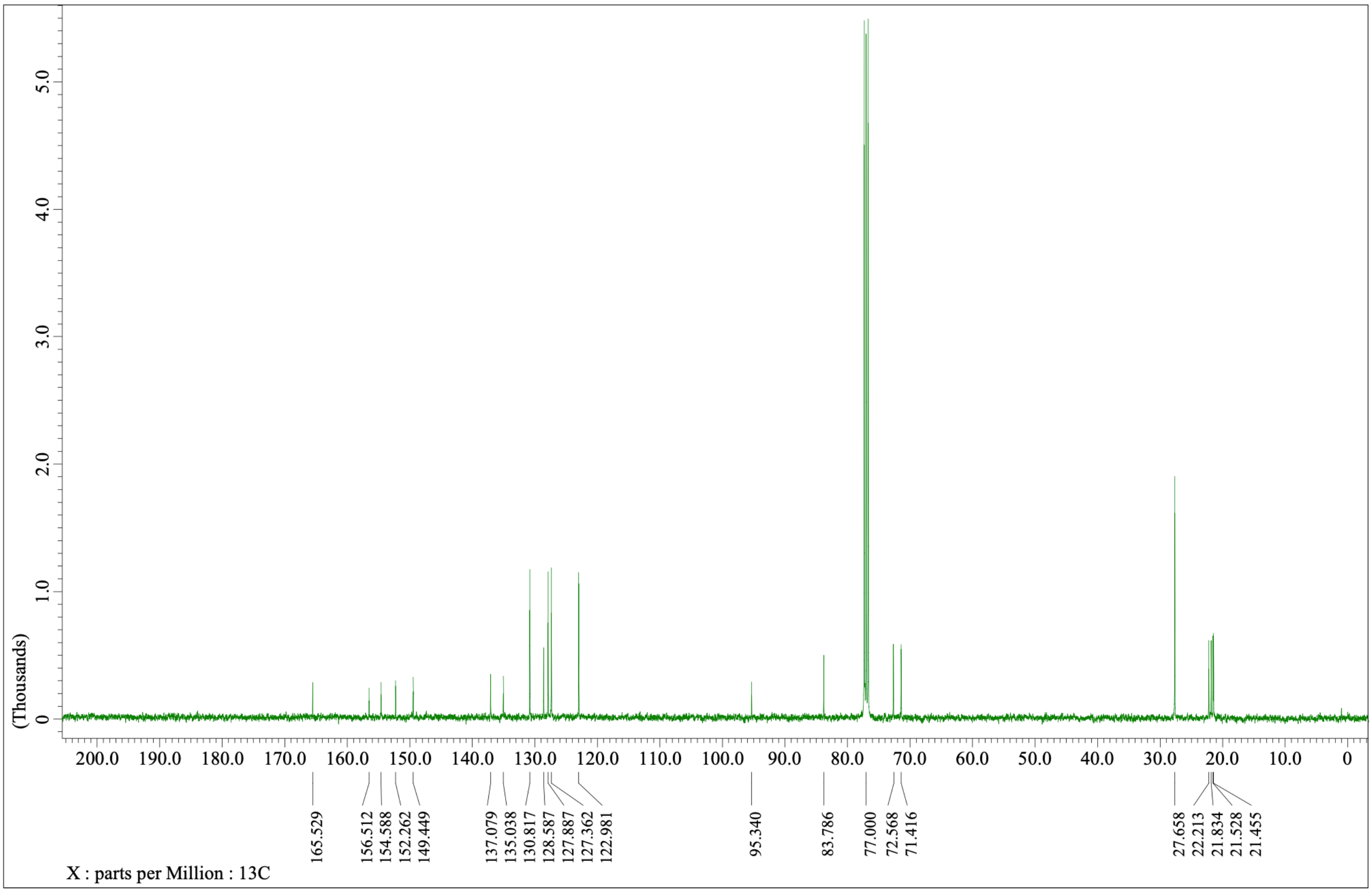

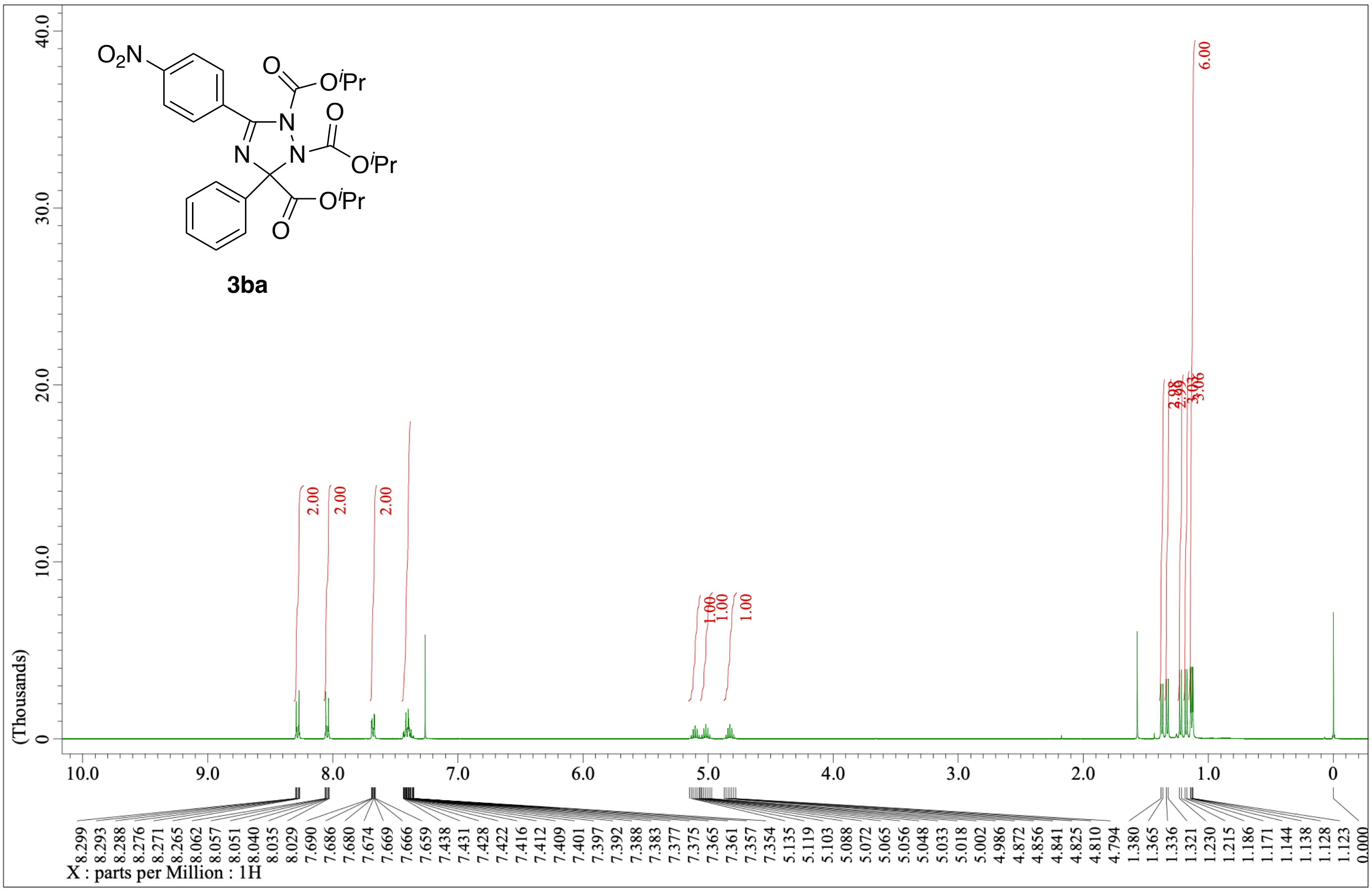

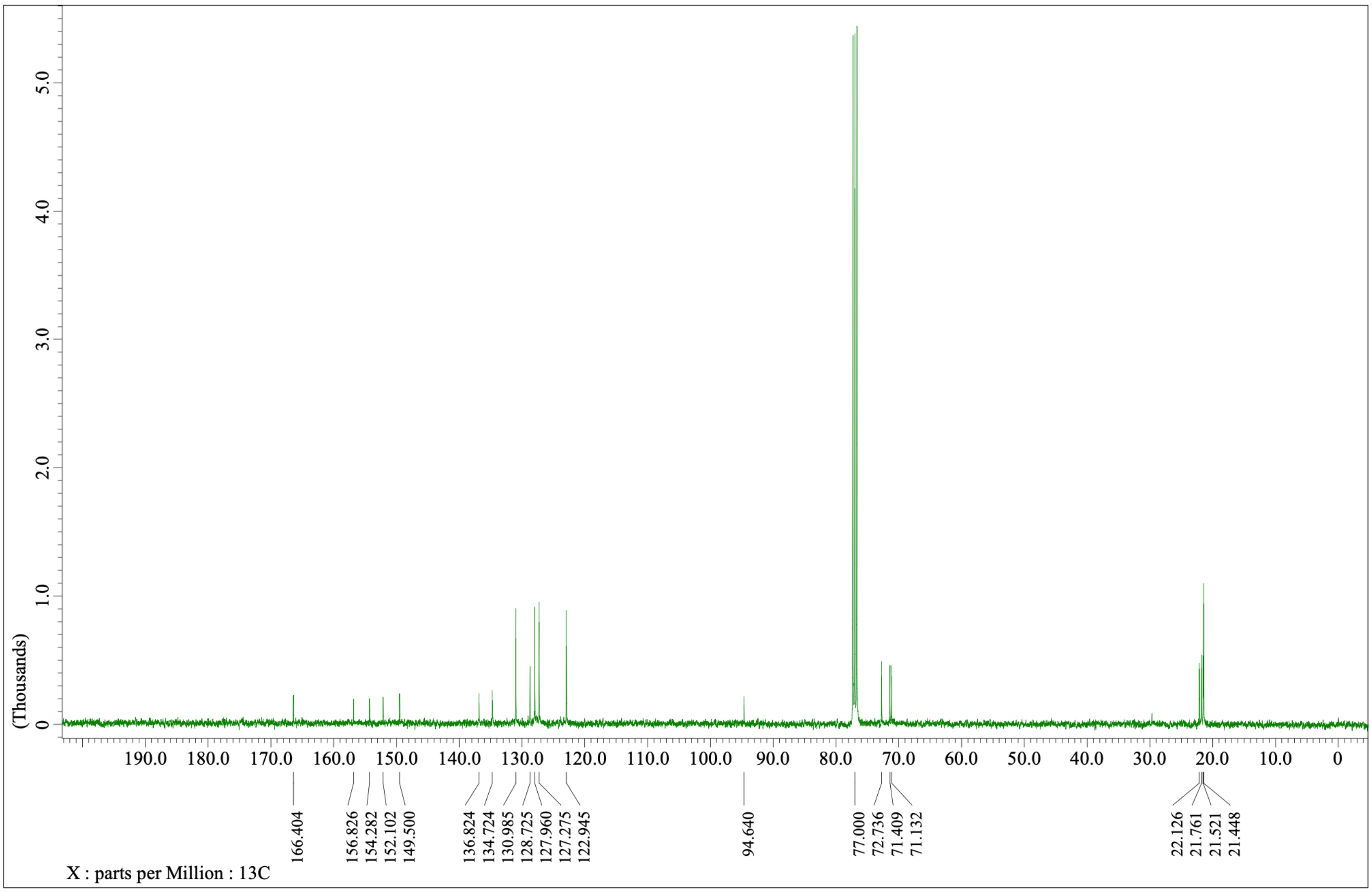

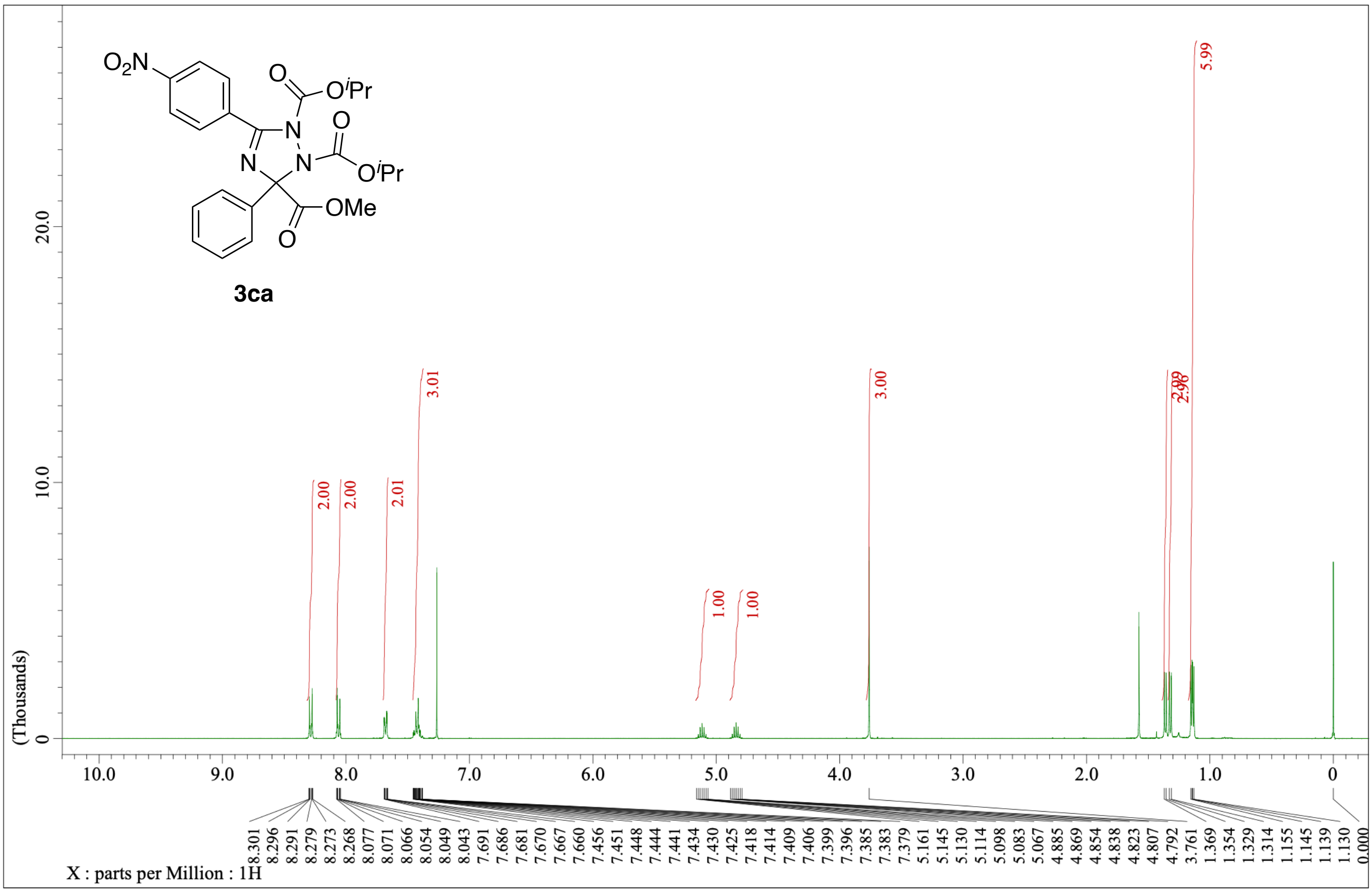
O_2N
O
O^iPr
N
N
N
O
O^iPr
OMe
O
3ca
2.00
2.00
2.01
3.01
1.00
1.00
3.00
2.99
2.96
5.99
(Thousands)
0
10.0
20.0
10.0 9.0 8.0 7.0 6.0 5.0 4.0 3.0 2.0 1.0 0
8.301 8.296 8.291 8.279 8.273 8.268 8.077 8.071 8.066 8.054 8.049 8.043 7.691 7.686 7.681 7.670 7.667 7.660 7.456 7.451 7.448 7.444 7.441 7.434 7.430 7.425 7.418 7.414 7.409 7.406 7.399 7.396 7.385 7.383 7.379 5.161 5.145 5.130 5.114 5.098 5.083 5.067 4.885 4.869 4.854 4.838 4.823 4.807 4.792 3.761 1.369 1.354 1.329 1.314 1.155 1.145 1.139 1.130 0.000
X : parts per Million : 1H

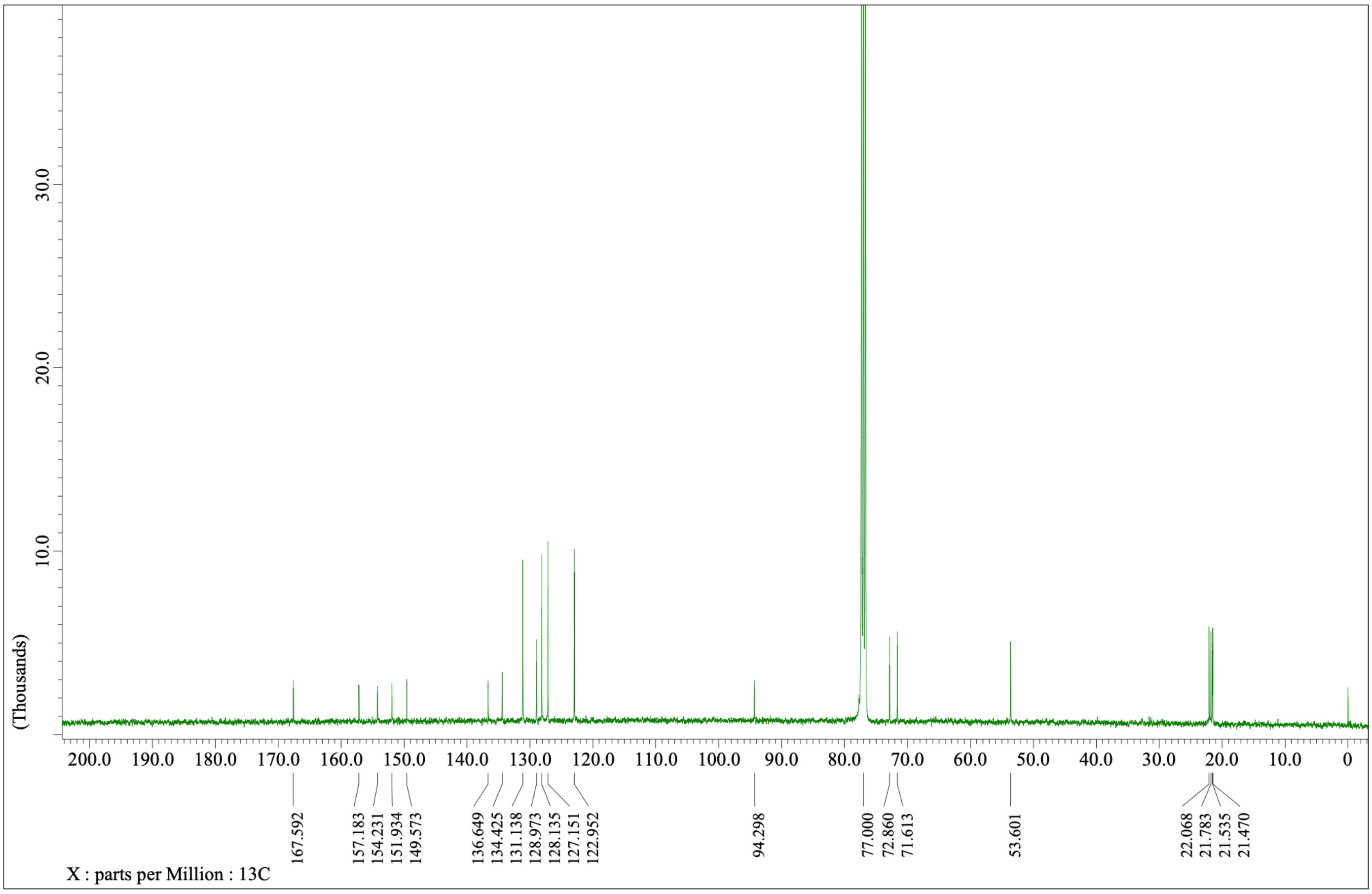
(Thousands)
10.0
20.0
30.0
200.0 190.0 180.0 170.0 160.0 150.0 140.0 130.0 120.0 110.0 100.0 90.0 80.0 70.0 60.0 50.0 40.0 30.0 20.0 10.0 0
167.592 157.183 154.231 151.934 149.573 136.649 134.425 131.138 128.973 128.135 127.151 122.952 94.298 77.000 72.860 71.613 53.601 22.068 21.783 21.535 21.470
X : parts per Million : 13C

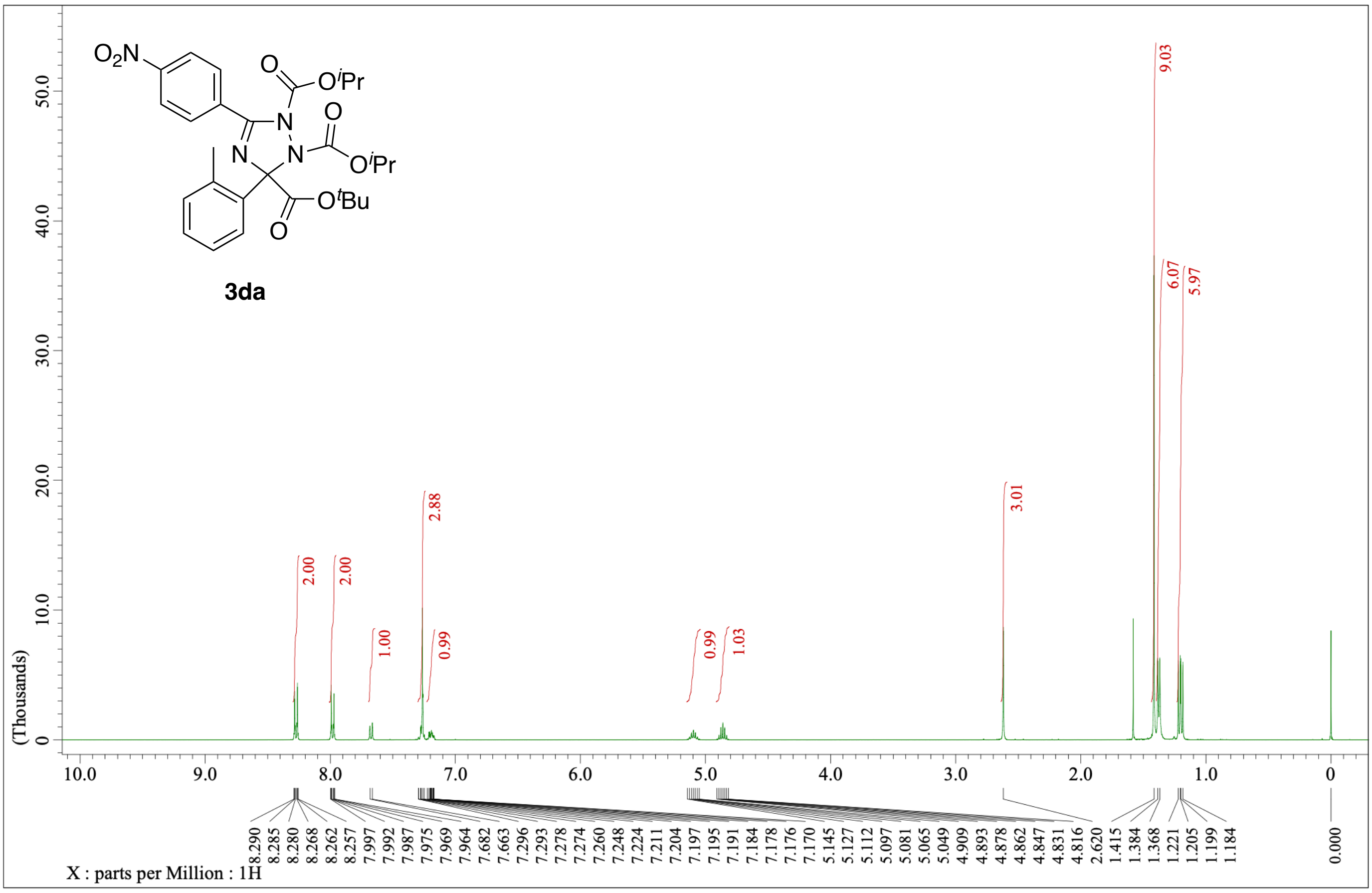

3da
(Thousands)
X : parts per Million : 1H

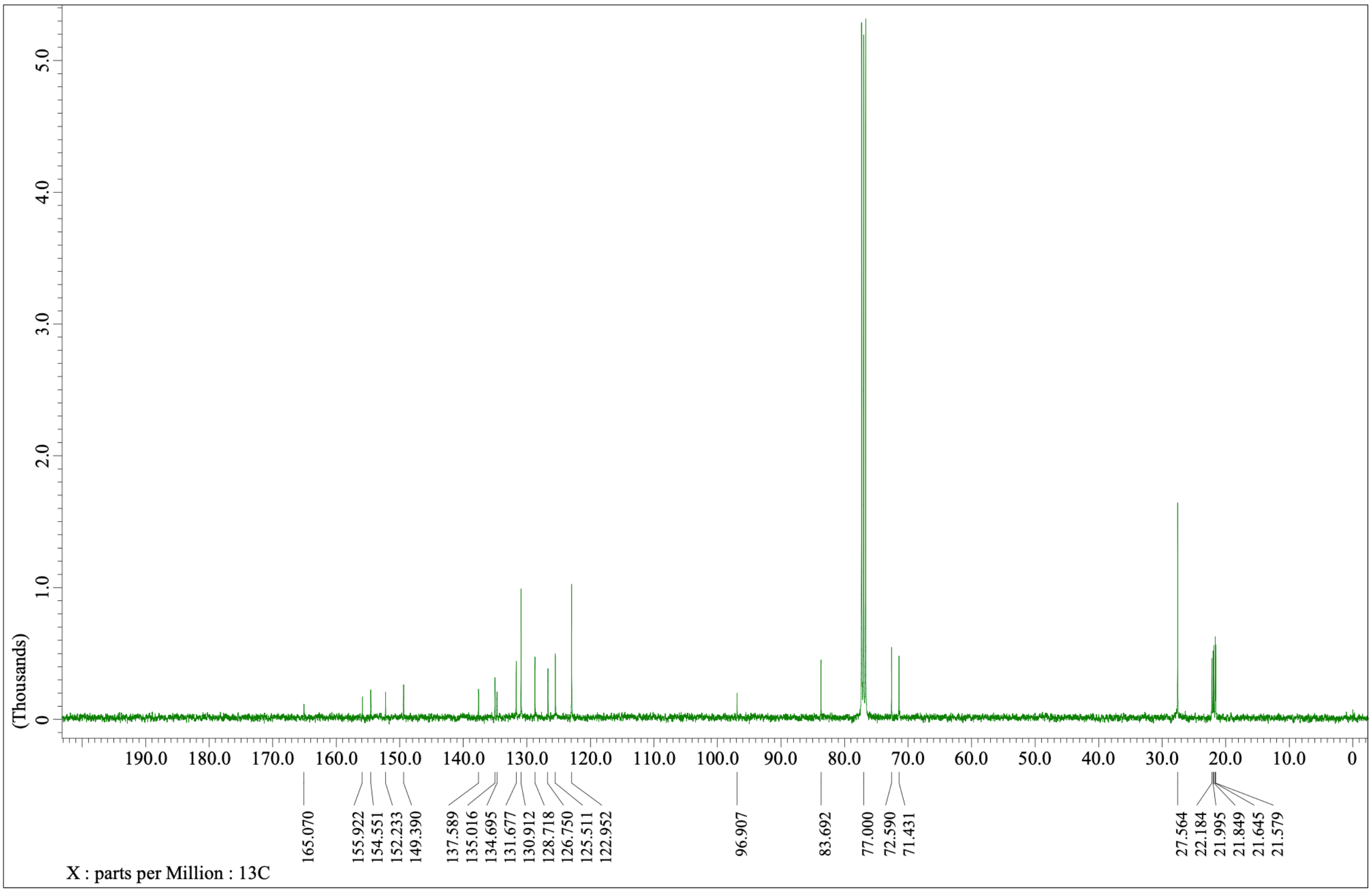

(Thousands)
X : parts per Million : 13C

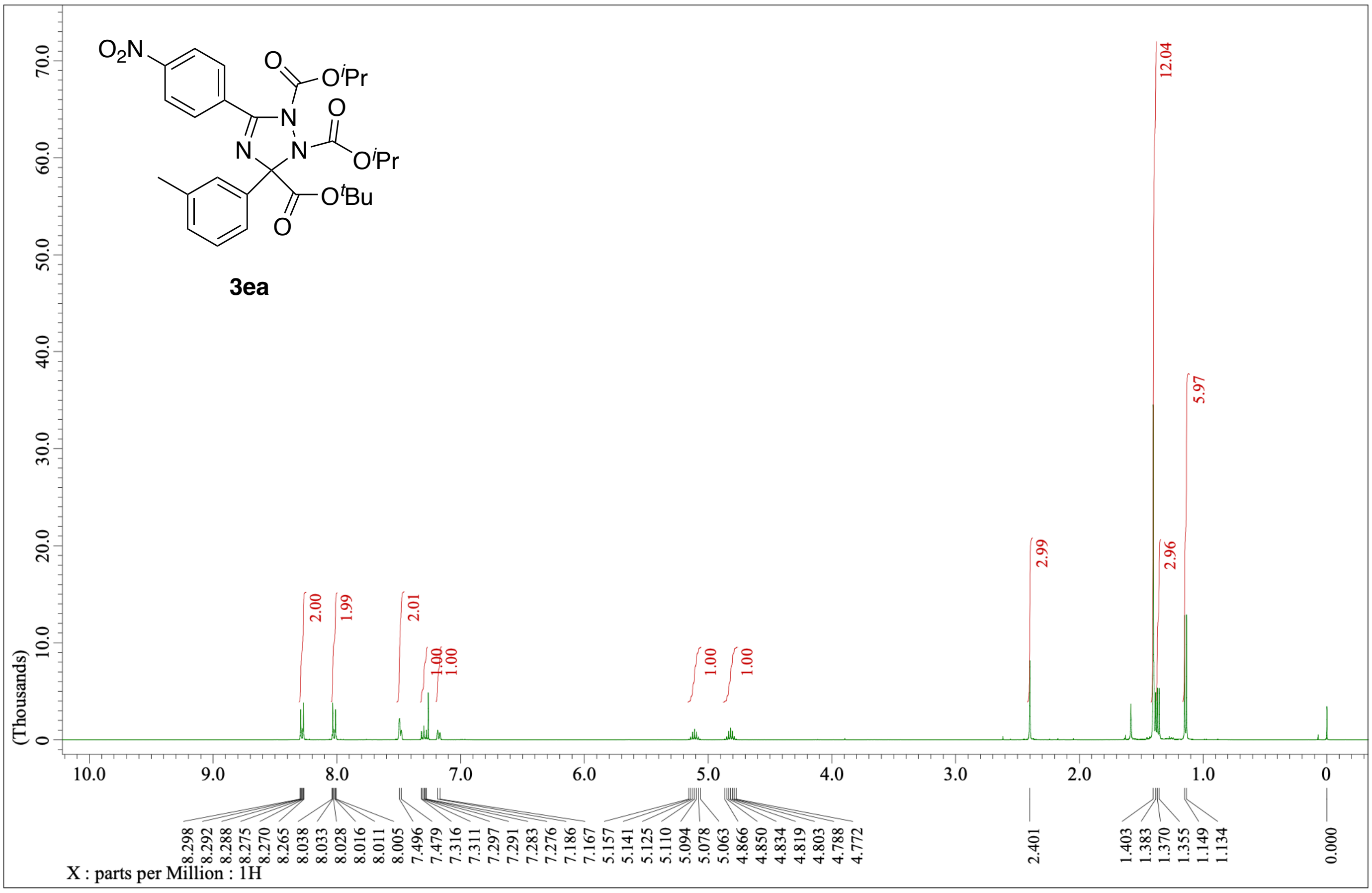

O2N
OiPr
OiPr
OtBu
3ea
(Thousands)
X : parts per Million : 1H
2.00
1.99
2.01
1.00
1.00
1.00
1.00
2.99
12.04
2.96
5.97
8.298 8.292 8.288 8.275 8.270 8.265 8.038 8.033 8.028 8.016 8.011 8.005 7.496 7.479 7.316 7.311 7.297 7.291 7.283 7.276 7.186 7.167 5.157 5.141 5.125 5.110 5.094 5.078 5.063 4.866 4.850 4.834 4.819 4.803 4.788 4.772
2.401
1.403 1.383 1.370 1.355 1.149 1.134
0.000

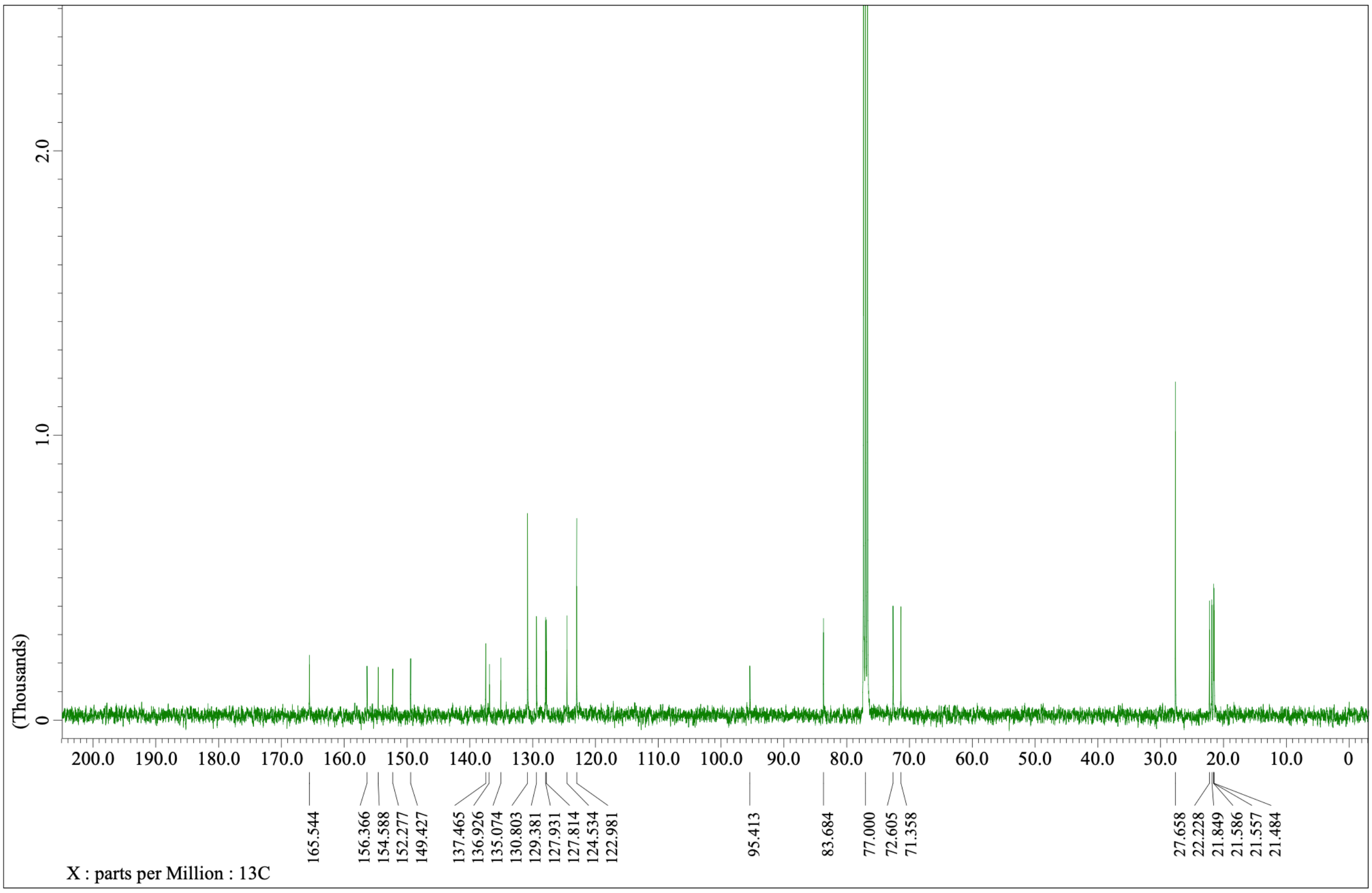

(Thousands)
X : parts per Million : 13C
165.544
156.366 154.588 152.277 149.427
137.465 136.926 135.074 130.803 129.381 127.931 127.814 124.534 122.981
95.413
83.684
77.000 72.605 71.358
27.658 22.228 21.849 21.586 21.557 21.484

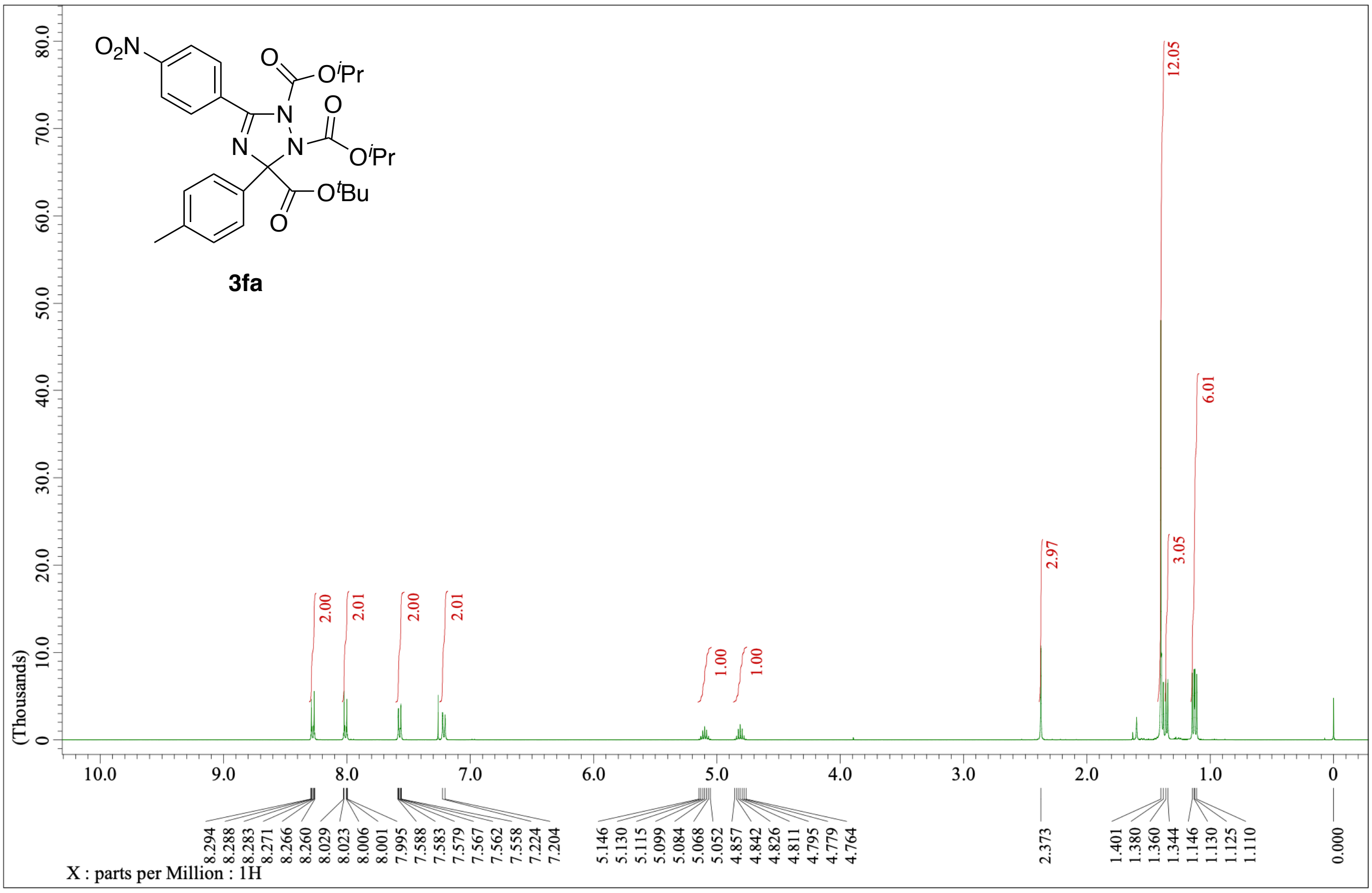

O2N
O
OiPr
N
N
O
N
OiPr
OtBu
O
3fa
(Thousands)
2.00 2.01 2.00 2.01 1.00 1.00 2.97 12.05 3.05 6.01
8.294 8.288 8.283 8.271 8.266 8.260 8.029 8.023 8.006 8.001 7.995 7.588 7.583 7.579 7.567 7.562 7.558 7.224 7.204 5.146 5.130 5.115 5.099 5.084 5.068 5.052 4.857 4.842 4.826 4.811 4.795 4.779 4.764 2.373 1.401 1.380 1.360 1.344 1.146 1.130 1.125 1.110 0.000
X : parts per Million : 1H

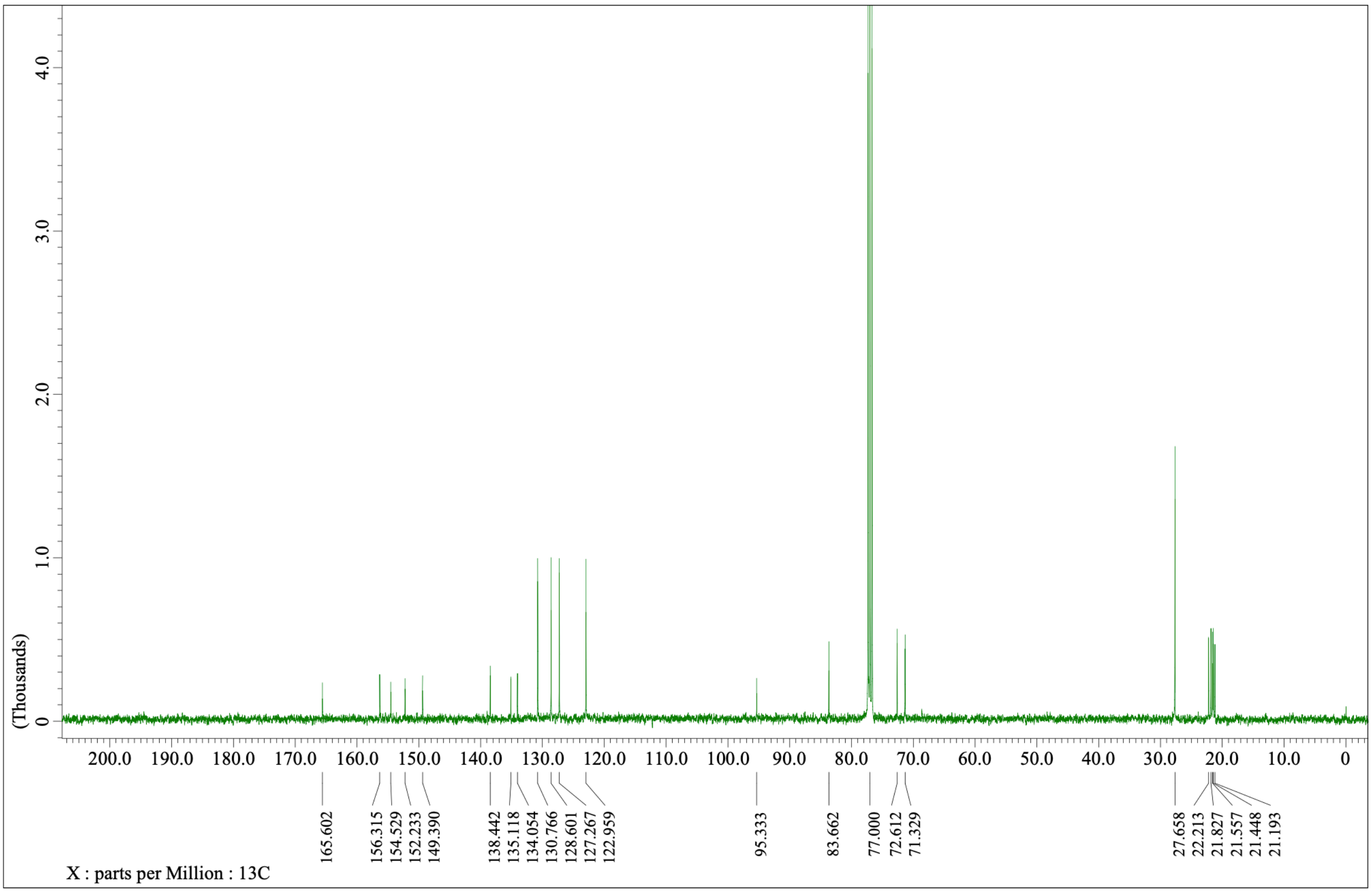

(Thousands)
165.602 156.315 154.529 152.233 149.390 138.442 135.118 134.054 130.766 128.601 127.267 122.959 95.333 83.662 77.000 72.612 71.329 27.658 22.213 21.827 21.557 21.448 21.193
X : parts per Million : 13C

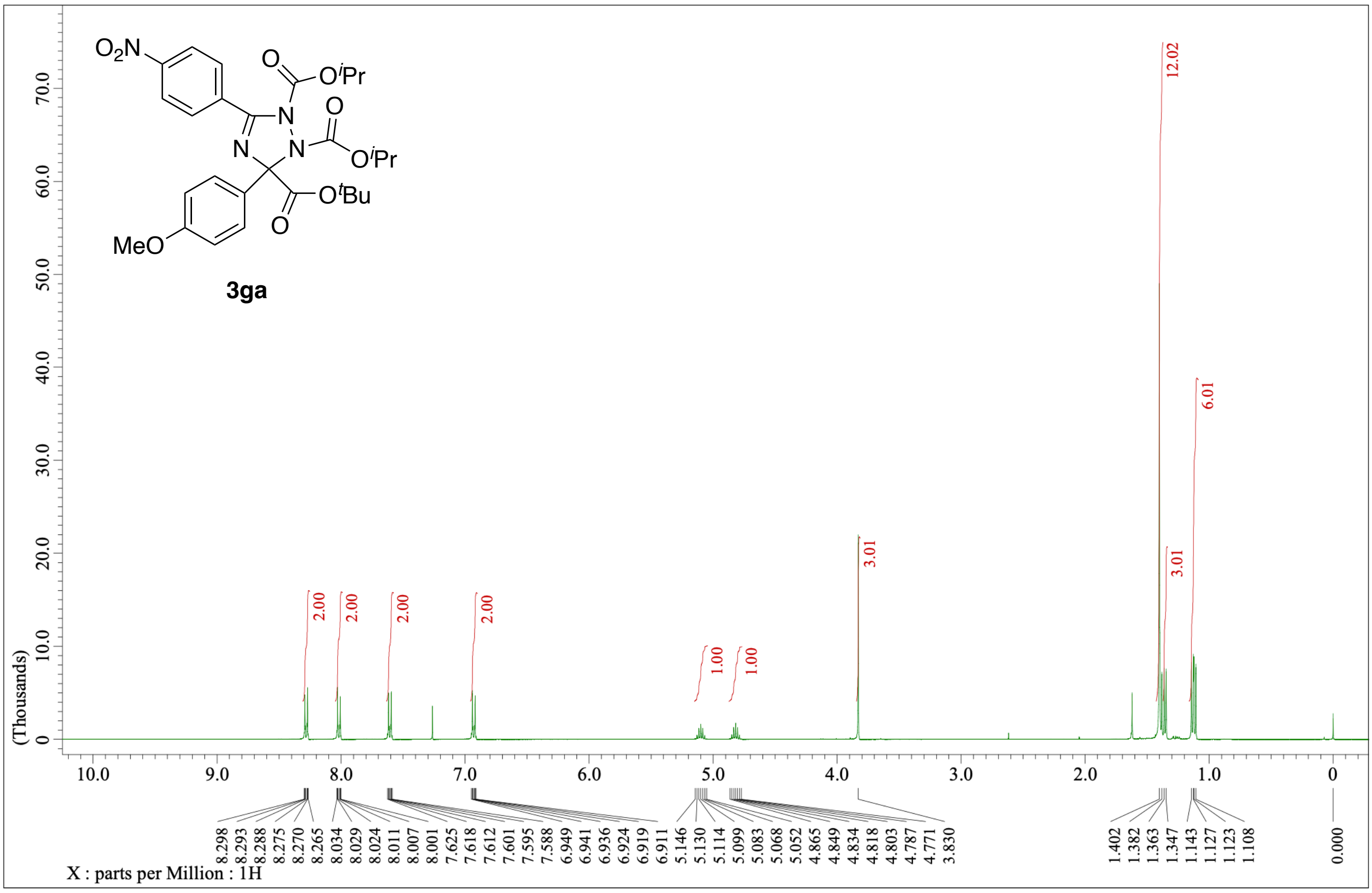

O_2N
O^{i}Pr
O^{t}Bu
MeO
3ga
(Thousands)
X : parts per Million : 1H
2.00
2.00
2.00
2.00
1.00
1.00
3.01
3.01
12.02
6.01
8.298 8.293 8.288 8.275 8.270 8.265 8.034 8.029 8.024 8.011 8.007 8.001 7.625 7.618 7.612 7.601 7.595 7.588 6.949 6.941 6.936 6.924 6.919 6.911 5.146 5.130 5.114 5.099 5.083 5.068 5.052 4.865 4.849 4.834 4.818 4.803 4.787 4.771 3.830
1.402 1.382 1.363 1.347 1.143 1.127 1.123 1.108
0.000

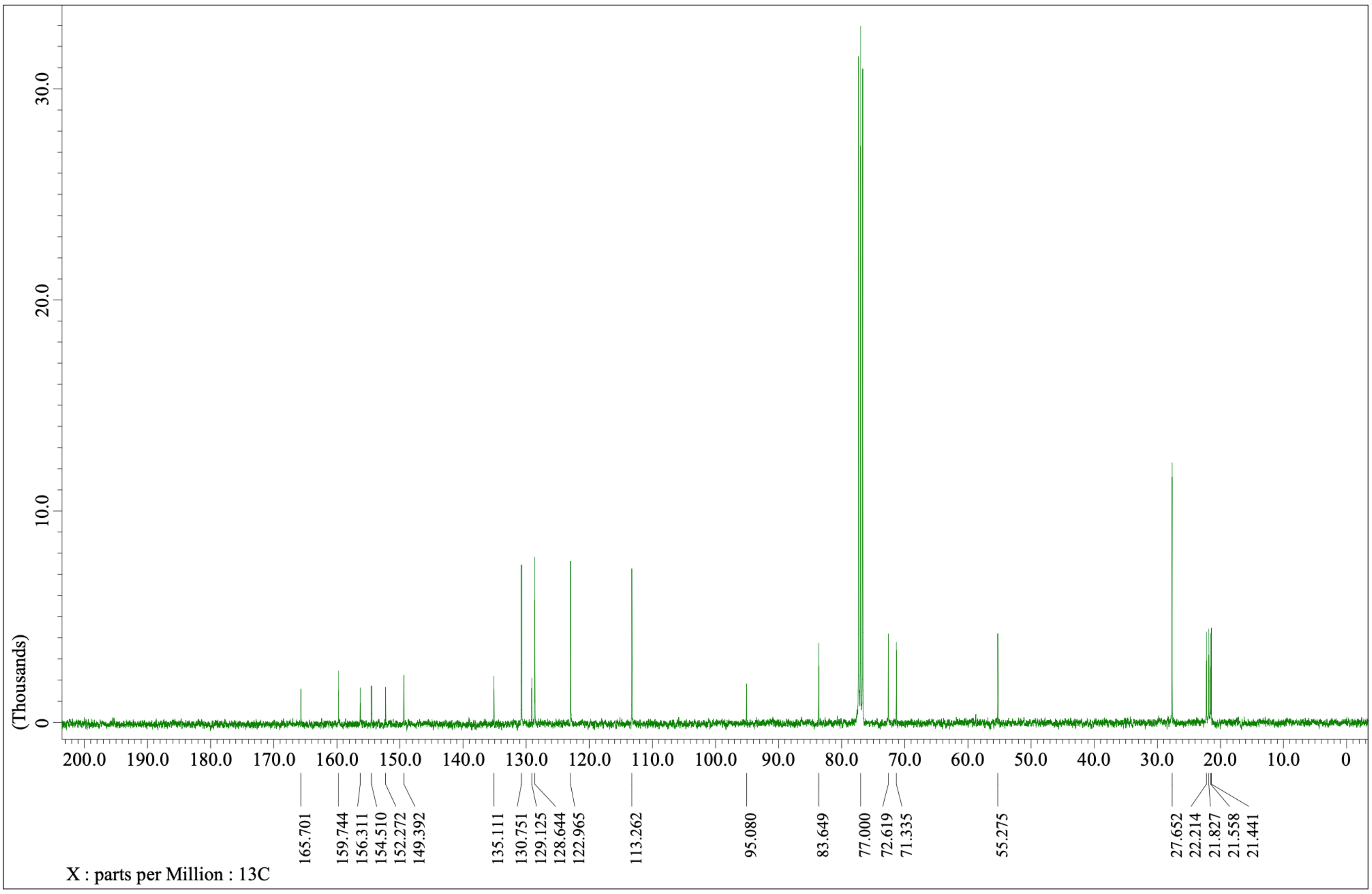

(Thousands)
X : parts per Million : 13C
165.701 159.744 156.311 154.510 152.272 149.392 135.111 130.751 129.125 128.644 122.965 113.262 95.080 83.649 77.000 72.619 71.335 55.275 27.652 22.214 21.827 21.558 21.441

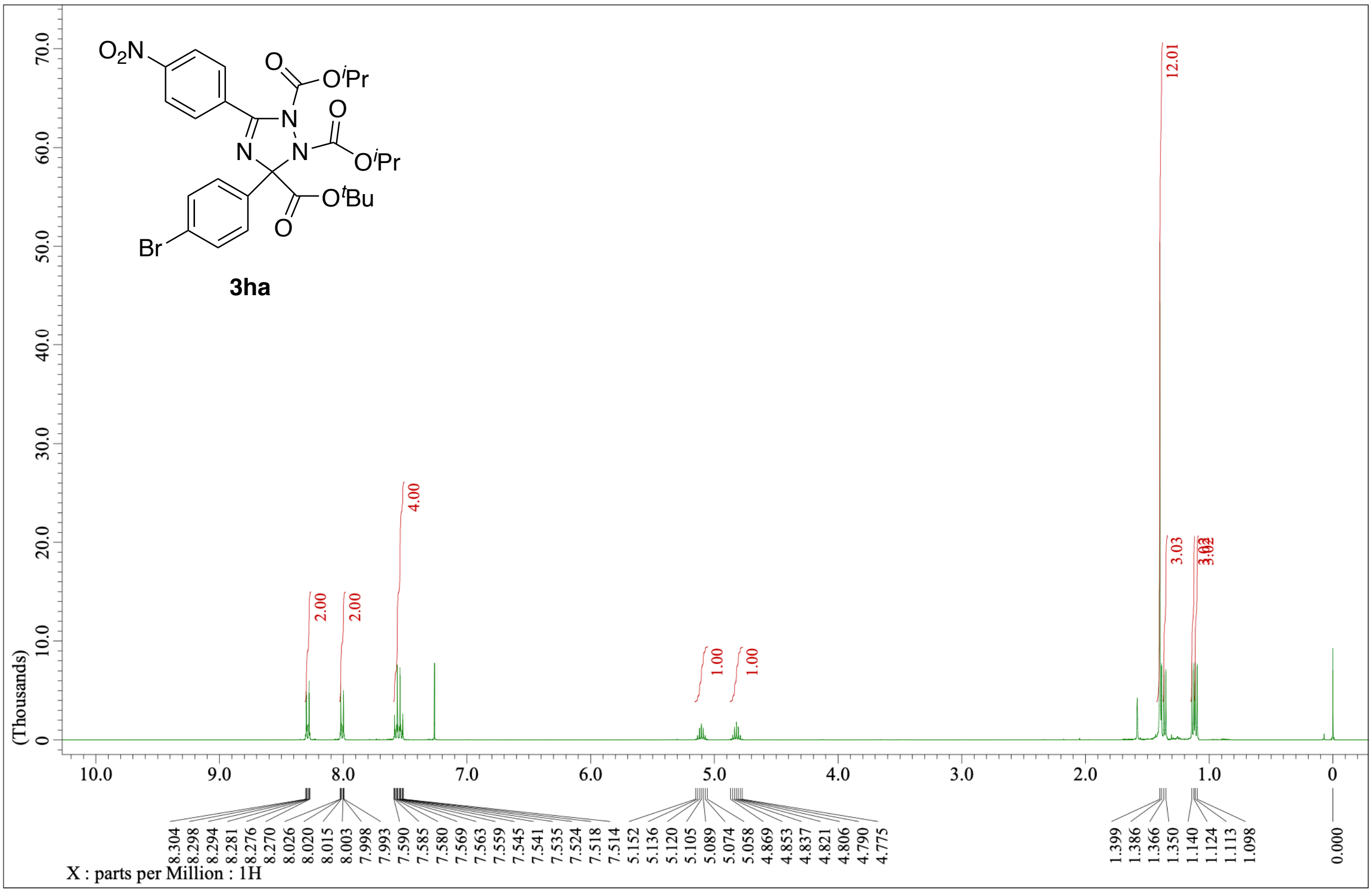
O2N
OiPr
O
N
N
N
OiPr
OtBu
Br
3ha
2.00
2.00
4.00
1.00
1.00
12.01
3.03
(Thousands)
X : parts per Million : 1H

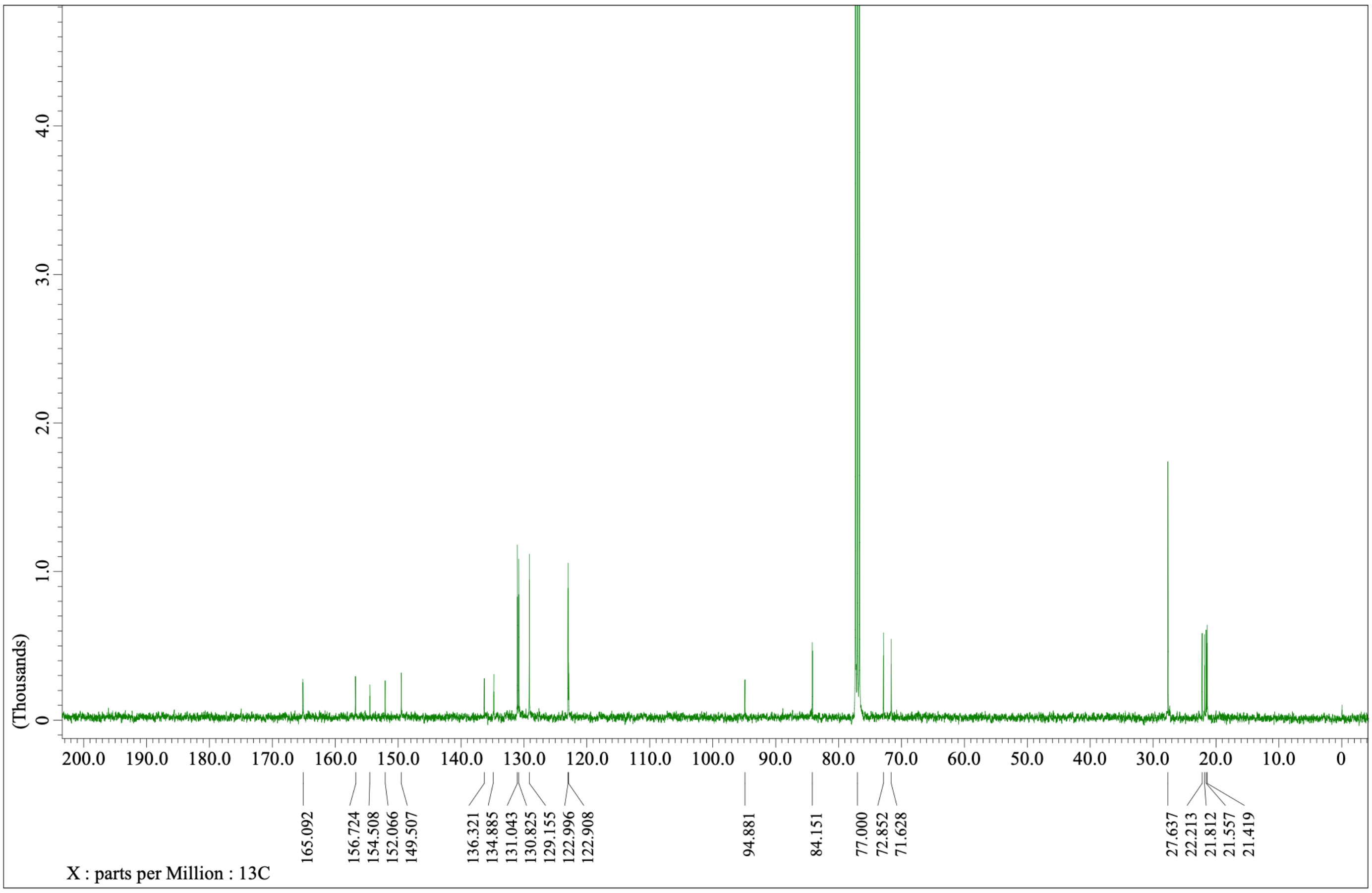
(Thousands)
X : parts per Million : 13C
165.092
156.724
154.508
152.066
149.507
136.321
134.885
131.043
130.825
129.155
122.996
122.908
94.881
84.151
77.000
72.852
71.628
27.637
22.213
21.812
21.557
21.419

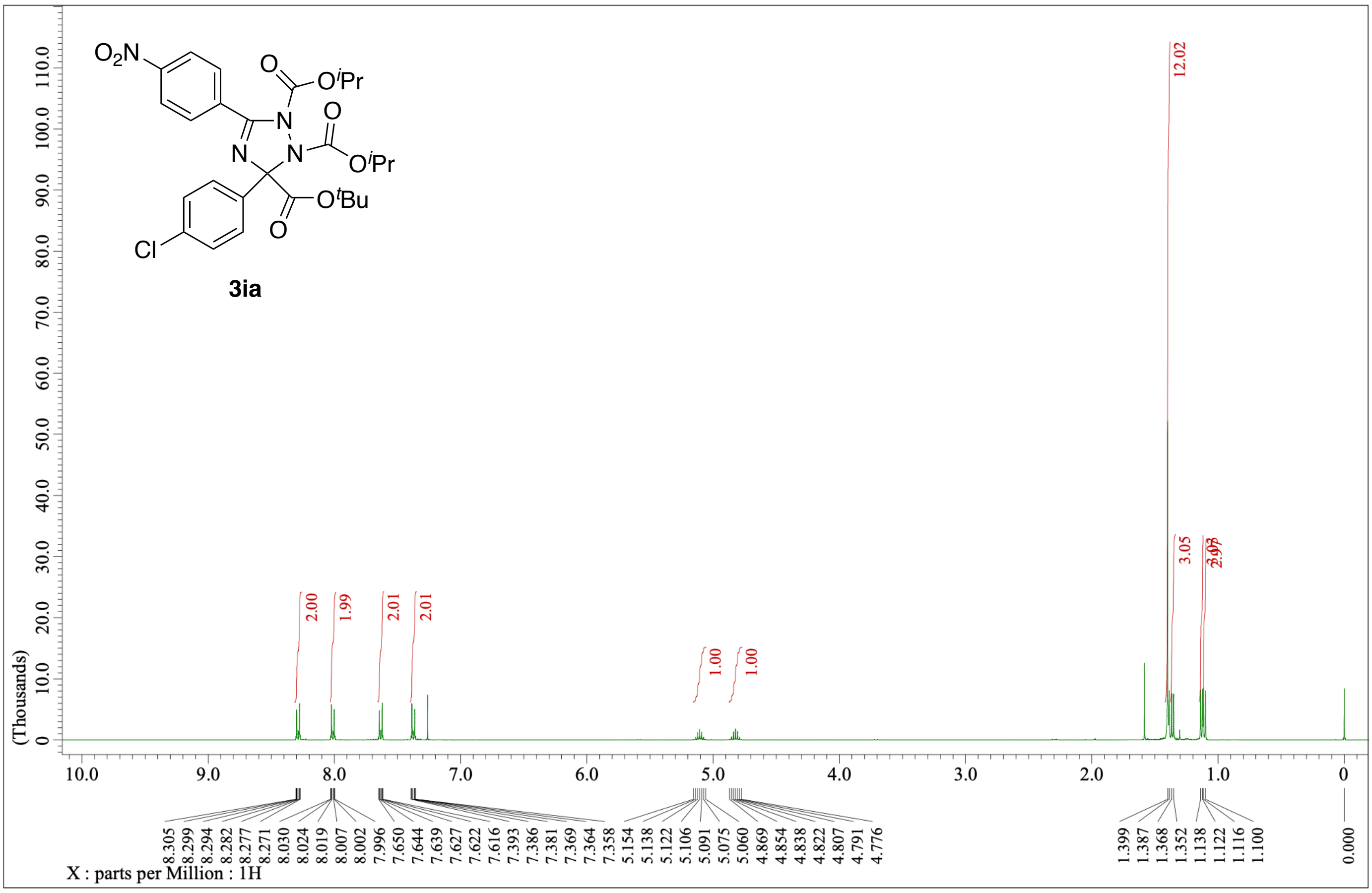

O2N
OiPr
OiPr
OtBu
Cl
3ia
X : parts per Million : 1H

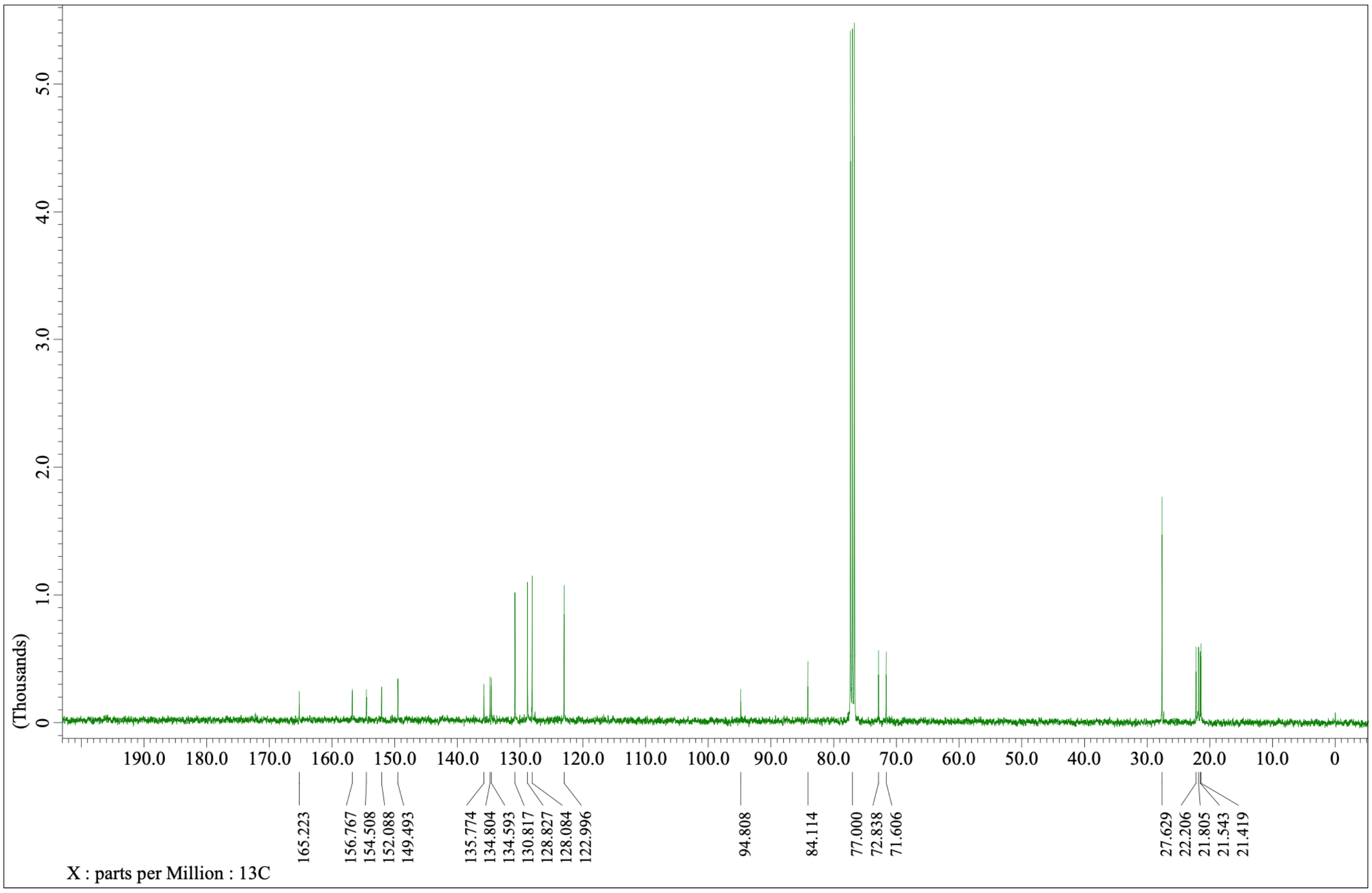

X : parts per Million : 13C

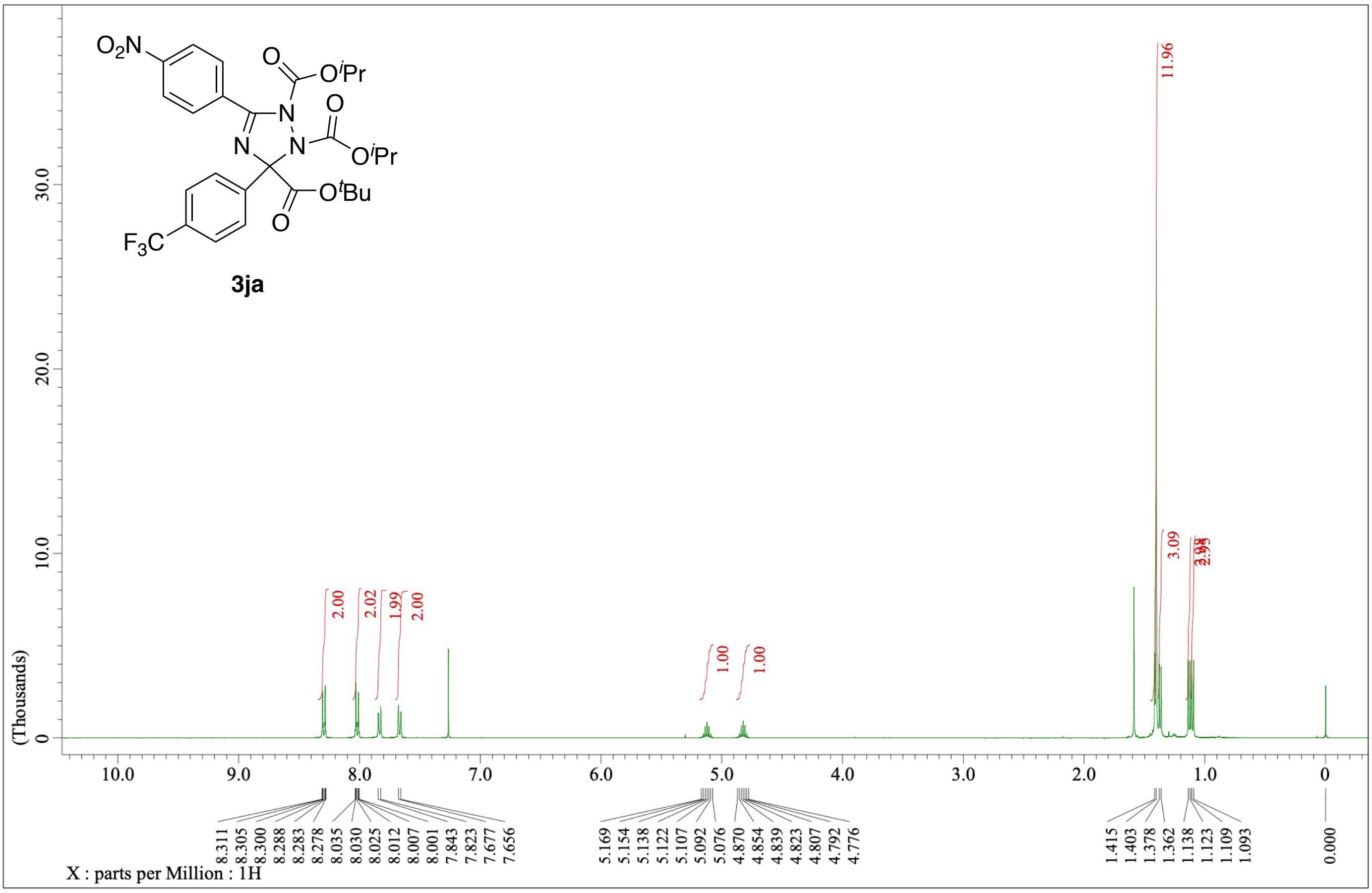

O_2N
O
O^iPr
N
O
N
N
O^iPr
O^tBu
O
F_3C
3ja
(Thousands)
30.0
20.0
10.0
0
2.00
2.02
1.99
2.00
1.00
1.00
11.96
3.09
10.0
9.0
8.0
7.0
6.0
5.0
4.0
3.0
2.0
1.0
0
8.311
8.305
8.300
8.288
8.283
8.278
8.035
8.030
8.025
8.012
8.007
8.001
7.843
7.823
7.677
7.656
5.169
5.154
5.138
5.122
5.107
5.092
5.076
4.870
4.854
4.839
4.823
4.807
4.792
4.776
1.415
1.403
1.378
1.362
1.138
1.123
1.109
1.093
0.000
X : parts per Million : 1H

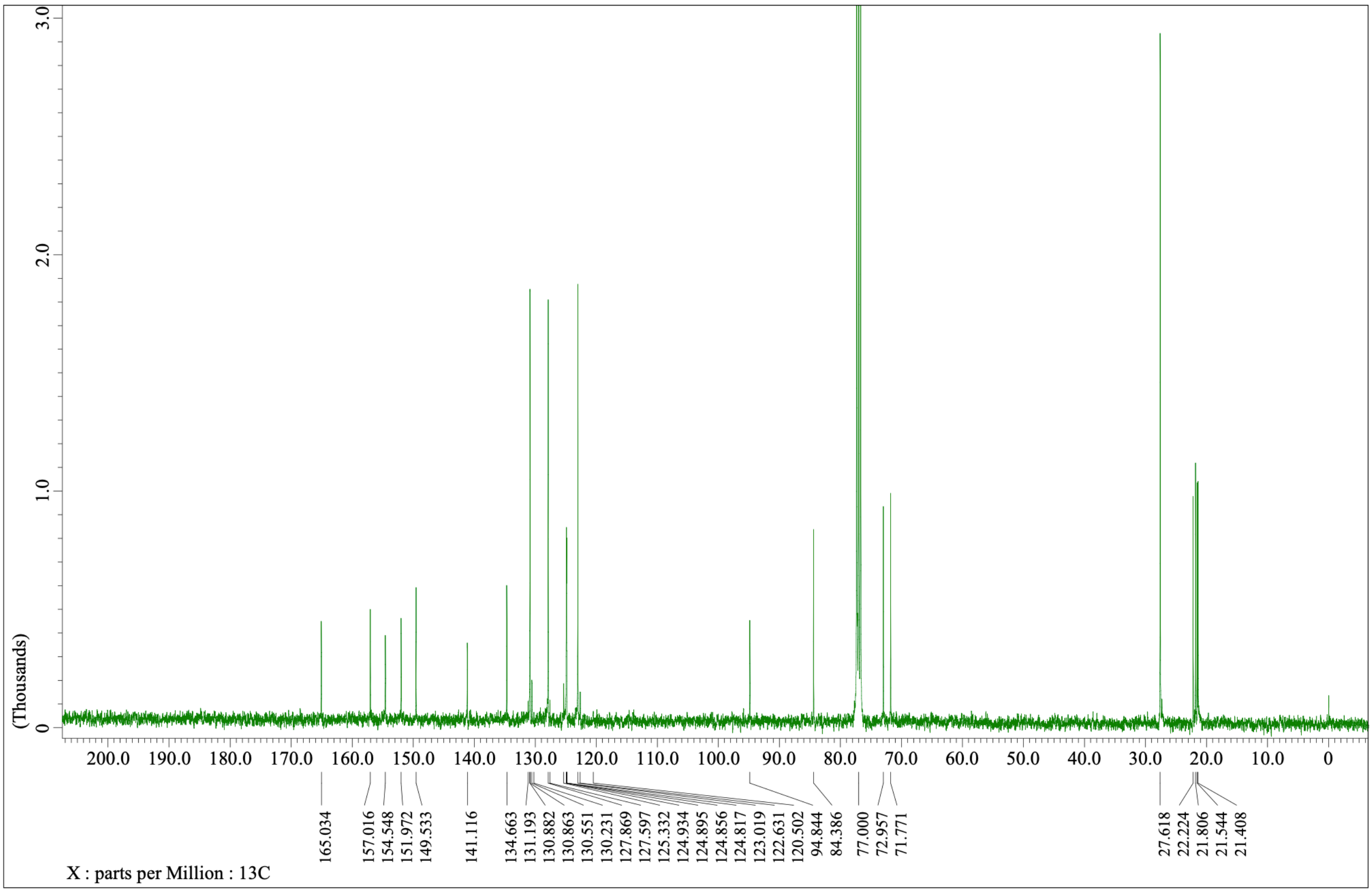

(Thousands)
3.0
2.0
1.0
0
200.0
190.0
180.0
170.0
160.0
150.0
140.0
130.0
120.0
110.0
100.0
90.0
80.0
70.0
60.0
50.0
40.0
30.0
20.0
10.0
0
165.034
157.016
154.548
151.972
149.533
141.116
134.663
131.193
130.882
130.863
130.551
130.231
127.869
127.597
125.332
124.934
124.895
124.856
124.817
123.019
122.631
120.502
94.844
84.386
77.000
72.957
71.771
27.618
22.224
21.806
21.544
21.408
X : parts per Million : 13C

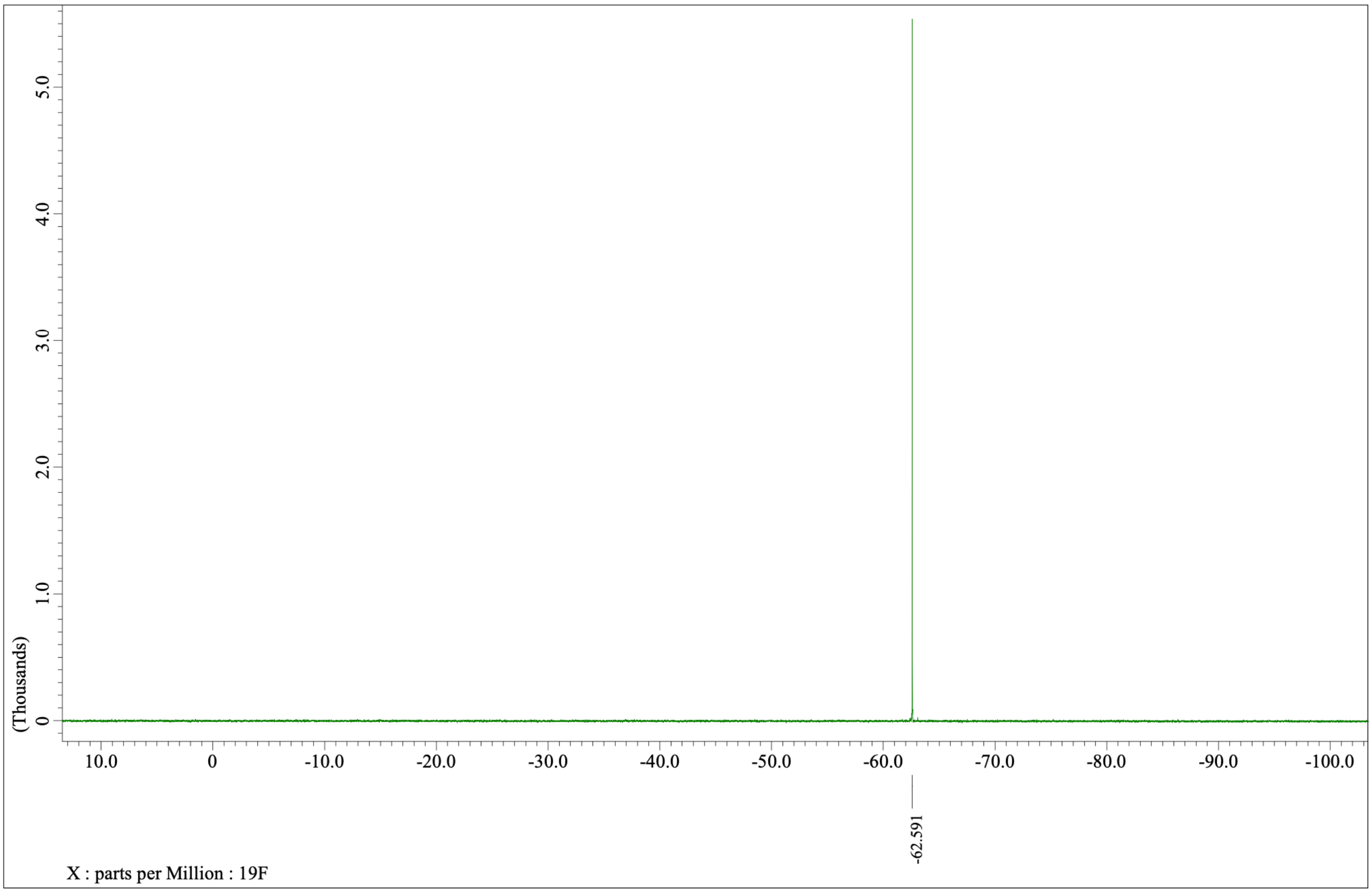

(Thousands)
5.0
4.0
3.0
2.0
1.0
0
10.0
0
-10.0
-20.0
-30.0
-40.0
-50.0
-60.0
-70.0
-80.0
-90.0
-100.0
-62.591
X : parts per Million : 19F

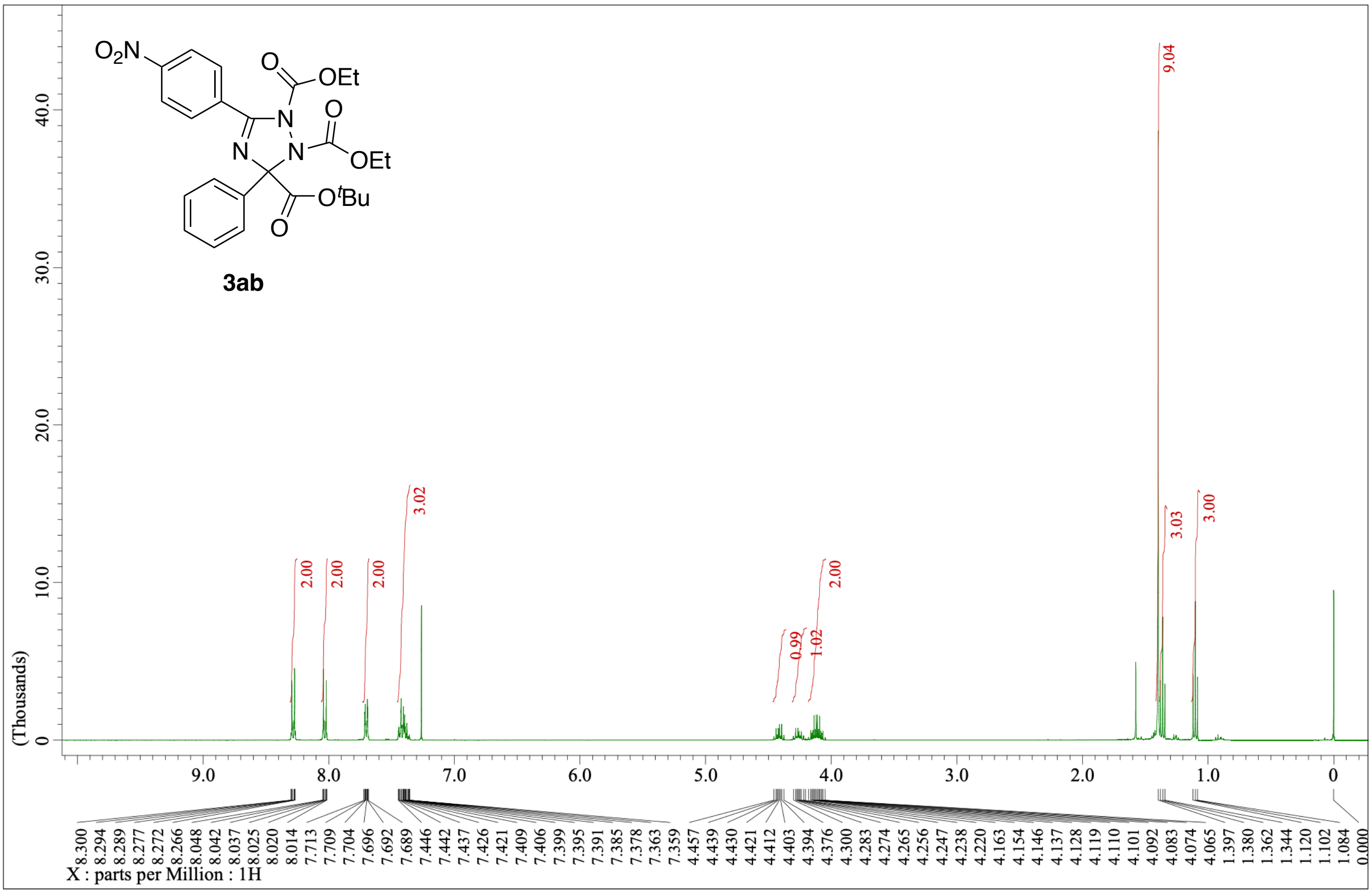
O2N
OEt
OEt
O
N
N
N
OtBu
3ab
(Thousands)
0
10.0
20.0
30.0
40.0
2.00
2.00
2.00
3.02
0.99
1.02
2.00
9.04
3.03
3.00
9.0
8.0
7.0
6.0
5.0
4.0
3.0
2.0
1.0
0
8.300
8.294
8.289
8.277
8.272
8.266
8.048
8.042
8.037
8.025
8.020
8.014
7.713
7.709
7.704
7.696
7.692
7.689
7.446
7.442
7.437
7.426
7.421
7.409
7.406
7.399
7.395
7.391
7.385
7.378
7.363
7.359
4.457
4.439
4.430
4.421
4.412
4.403
4.394
4.376
4.300
4.283
4.274
4.265
4.256
4.247
4.238
4.220
4.163
4.154
4.146
4.137
4.128
4.119
4.110
4.101
4.092
4.083
4.074
4.065
1.397
1.380
1.362
1.344
1.120
1.102
1.084
0.000
X : parts per Million : 1H

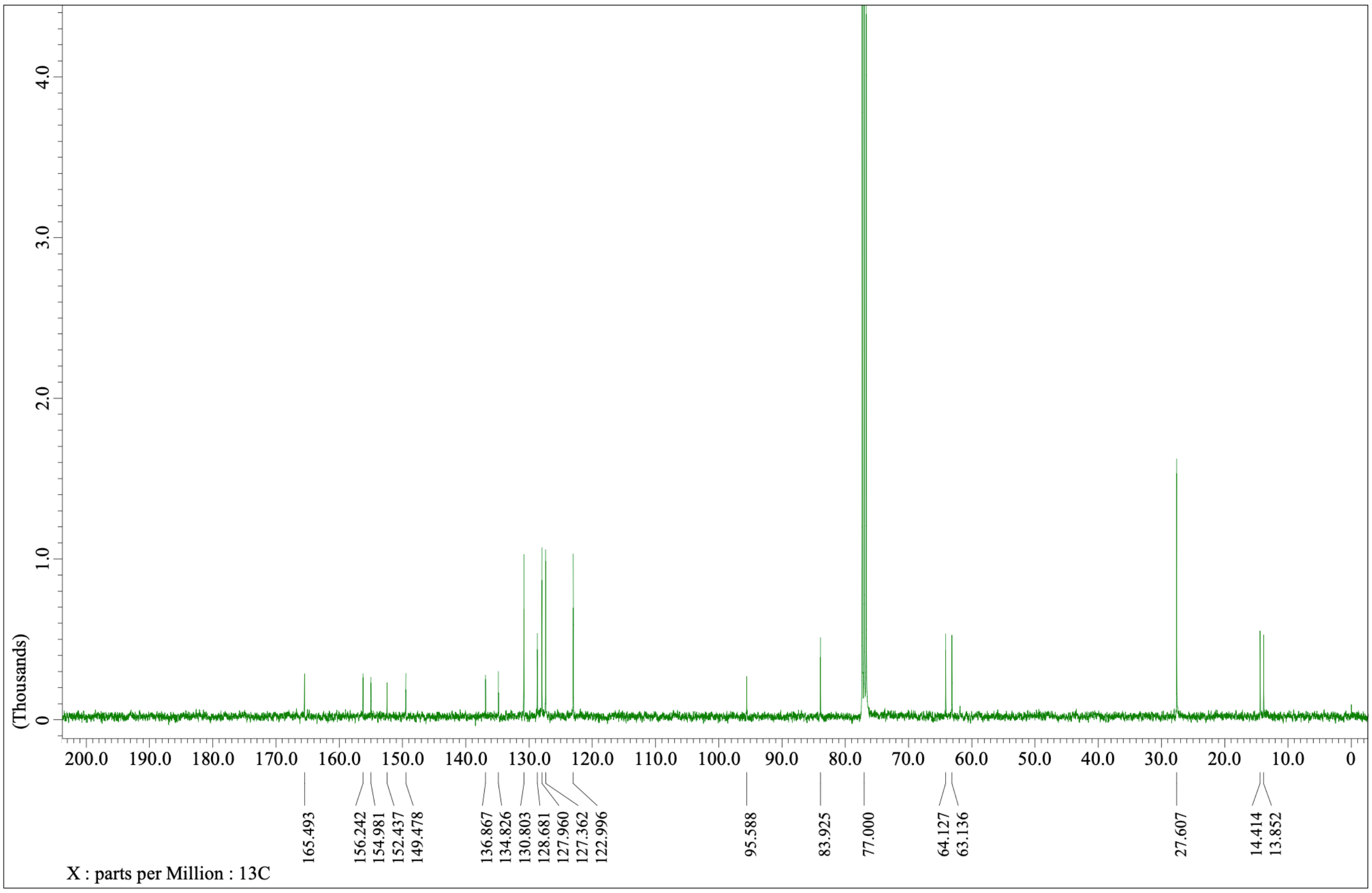
(Thousands)
0
1.0
2.0
3.0
4.0
200.0
190.0
180.0
170.0
160.0
150.0
140.0
130.0
120.0
110.0
100.0
90.0
80.0
70.0
60.0
50.0
40.0
30.0
20.0
10.0
0
165.493
156.242
154.981
152.437
149.478
136.867
134.826
130.803
128.681
127.960
127.362
122.996
95.588
83.925
77.000
64.127
63.136
27.607
14.414
13.852
X : parts per Million : 13C

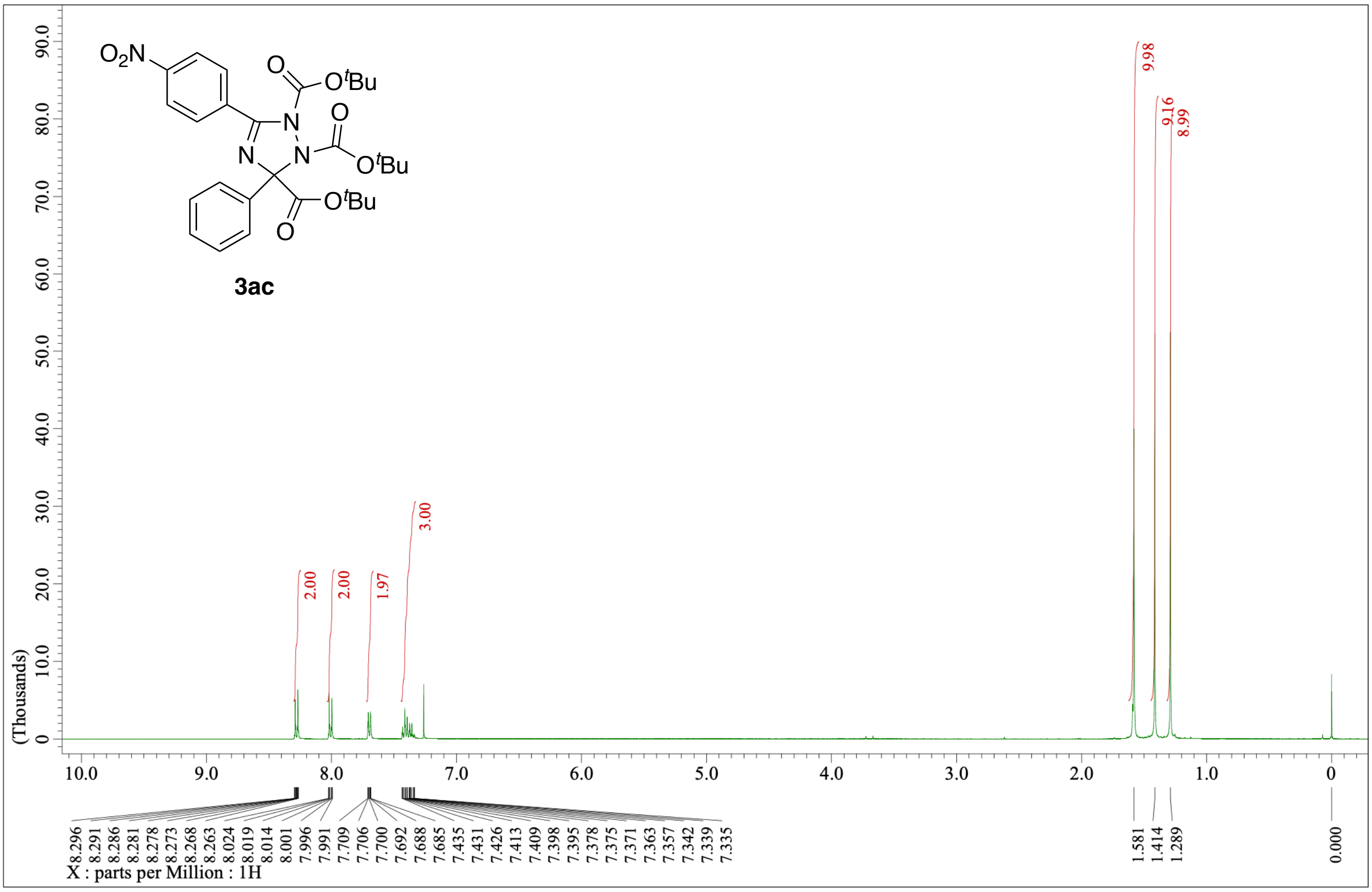

3ac
X : parts per Million : 1H
(Thousands)
8.296 8.291 8.286 8.281 8.278 8.273 8.268 8.263 8.024 8.019 8.014 8.001 7.996 7.991 7.709 7.706 7.700 7.692 7.688 7.685 7.435 7.431 7.426 7.413 7.409 7.398 7.395 7.378 7.375 7.371 7.363 7.357 7.342 7.339 7.335
1.581 1.414 1.289
0.000
2.00 2.00 1.97 3.00 9.98 9.16 8.99

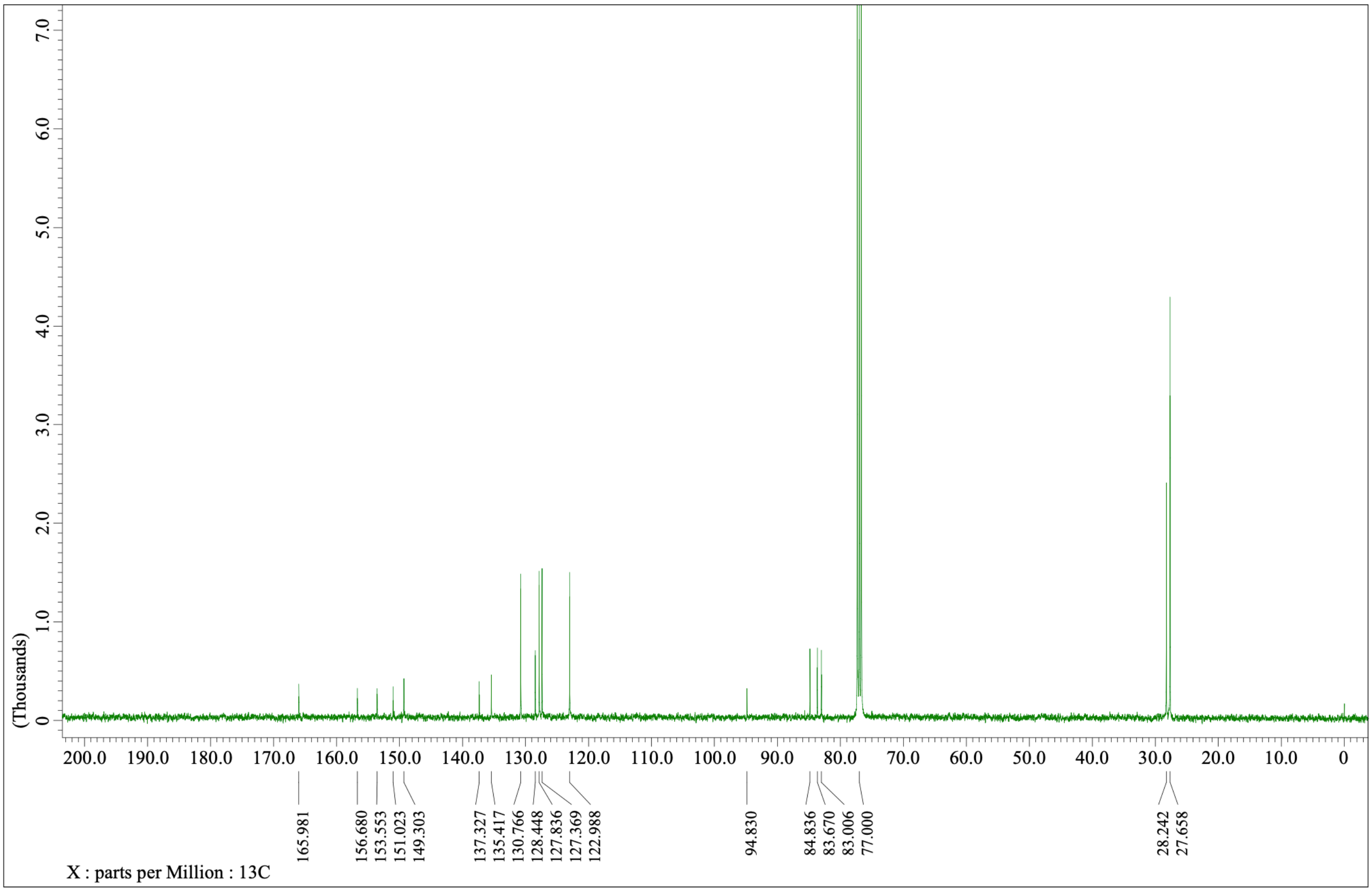

X : parts per Million : 13C
(Thousands)
165.981 156.680 153.553 151.023 149.303 137.327 135.417 130.766 128.448 127.836 127.369 122.988 94.830 84.836 83.670 83.006 77.000 28.242 27.658

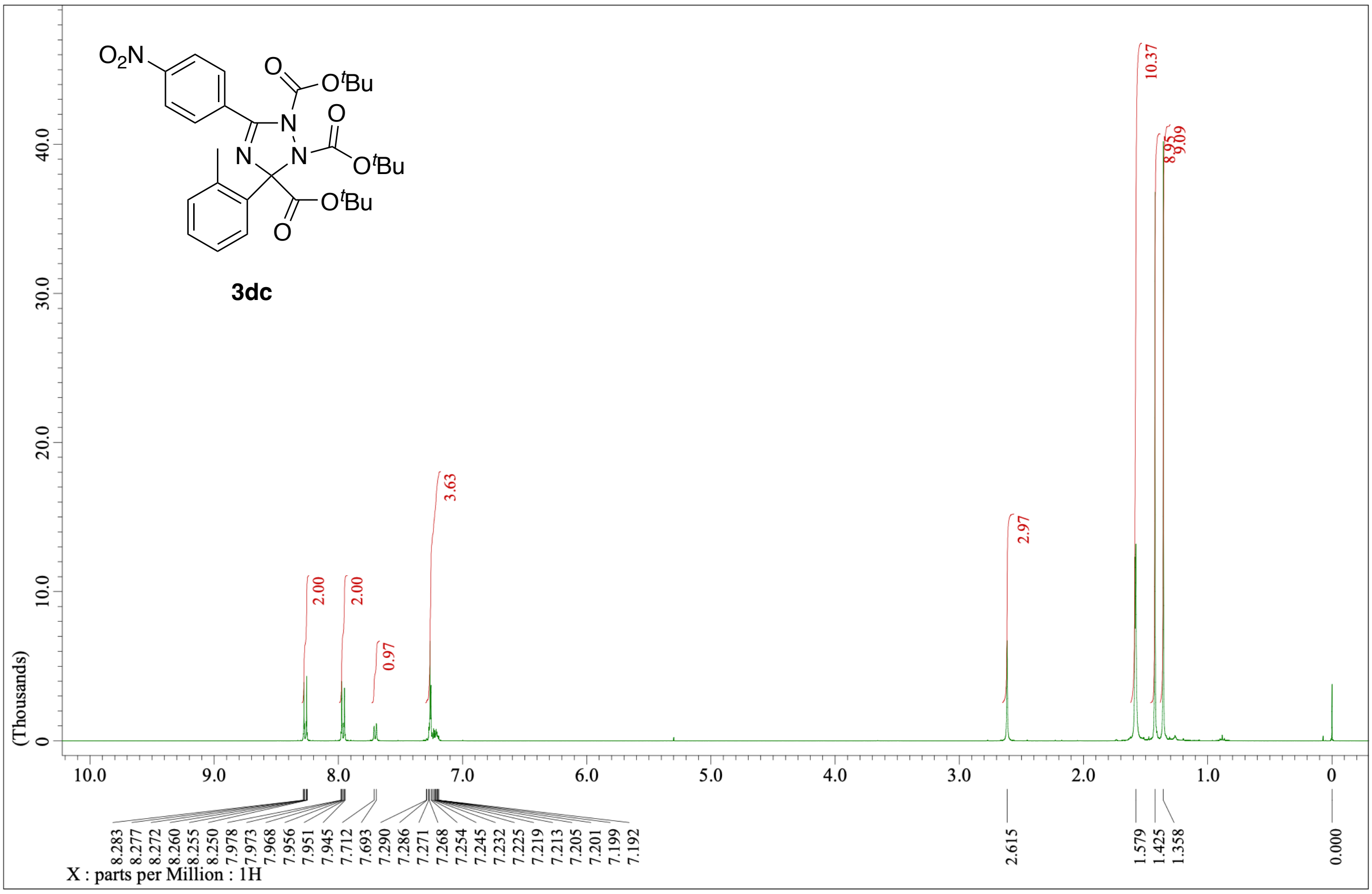

3dc
(Thousands)
X : parts per Million : 1H
10.37
8.95
9.09
2.97
3.63
2.00
2.00
0.97
8.283
8.277
8.272
8.260
8.255
8.250
7.978
7.973
7.968
7.956
7.951
7.945
7.712
7.693
7.290
7.286
7.271
7.268
7.254
7.245
7.232
7.225
7.219
7.213
7.205
7.201
7.199
7.192
2.615
1.579
1.425
1.358
0.000

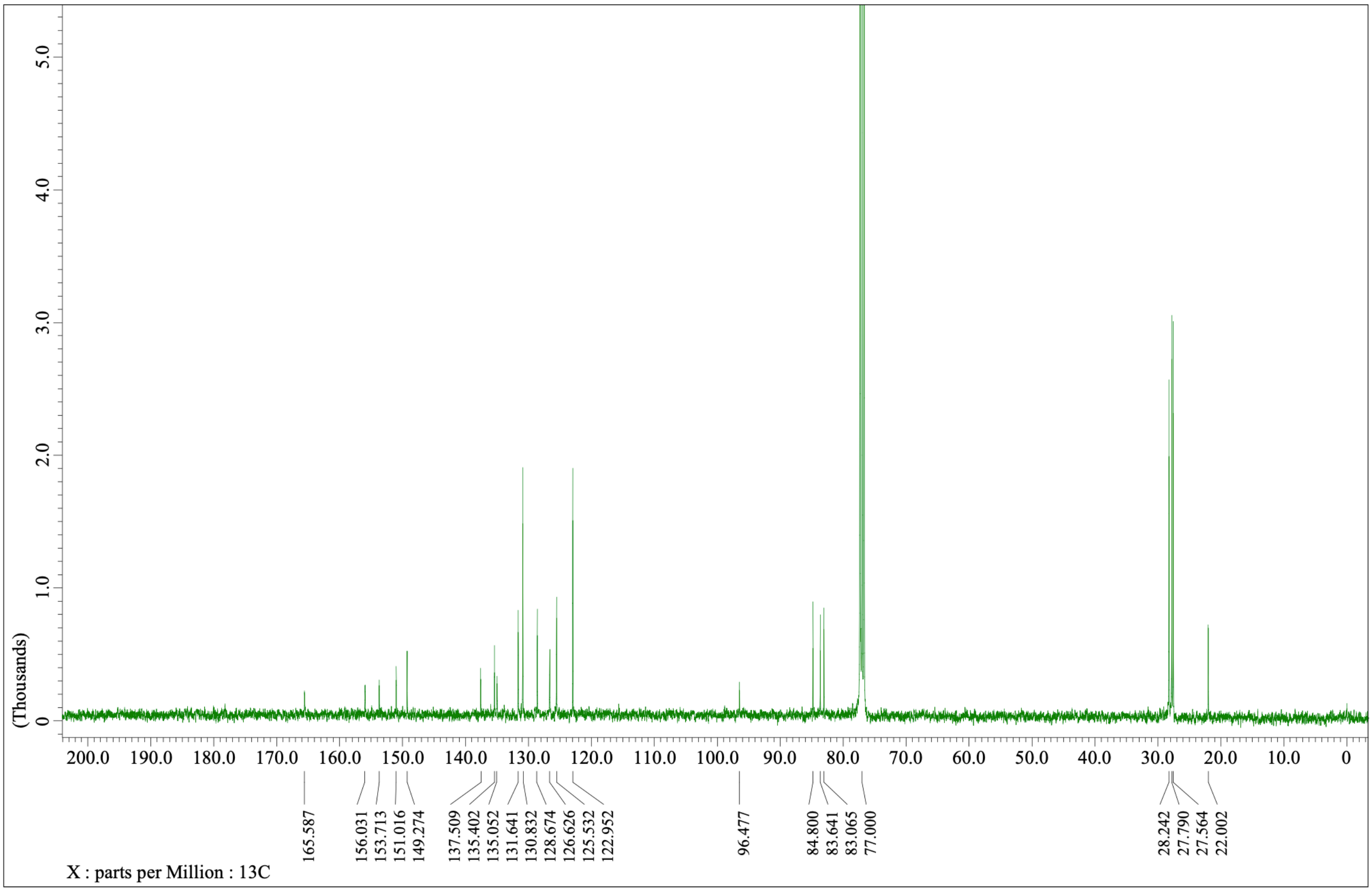

(Thousands)
X : parts per Million : 13C
165.587
156.031
153.713
151.016
149.274
137.509
135.402
135.052
131.641
130.832
128.674
126.626
125.532
122.952
96.477
84.800
83.641
83.065
77.000
28.242
27.790
27.564
22.002

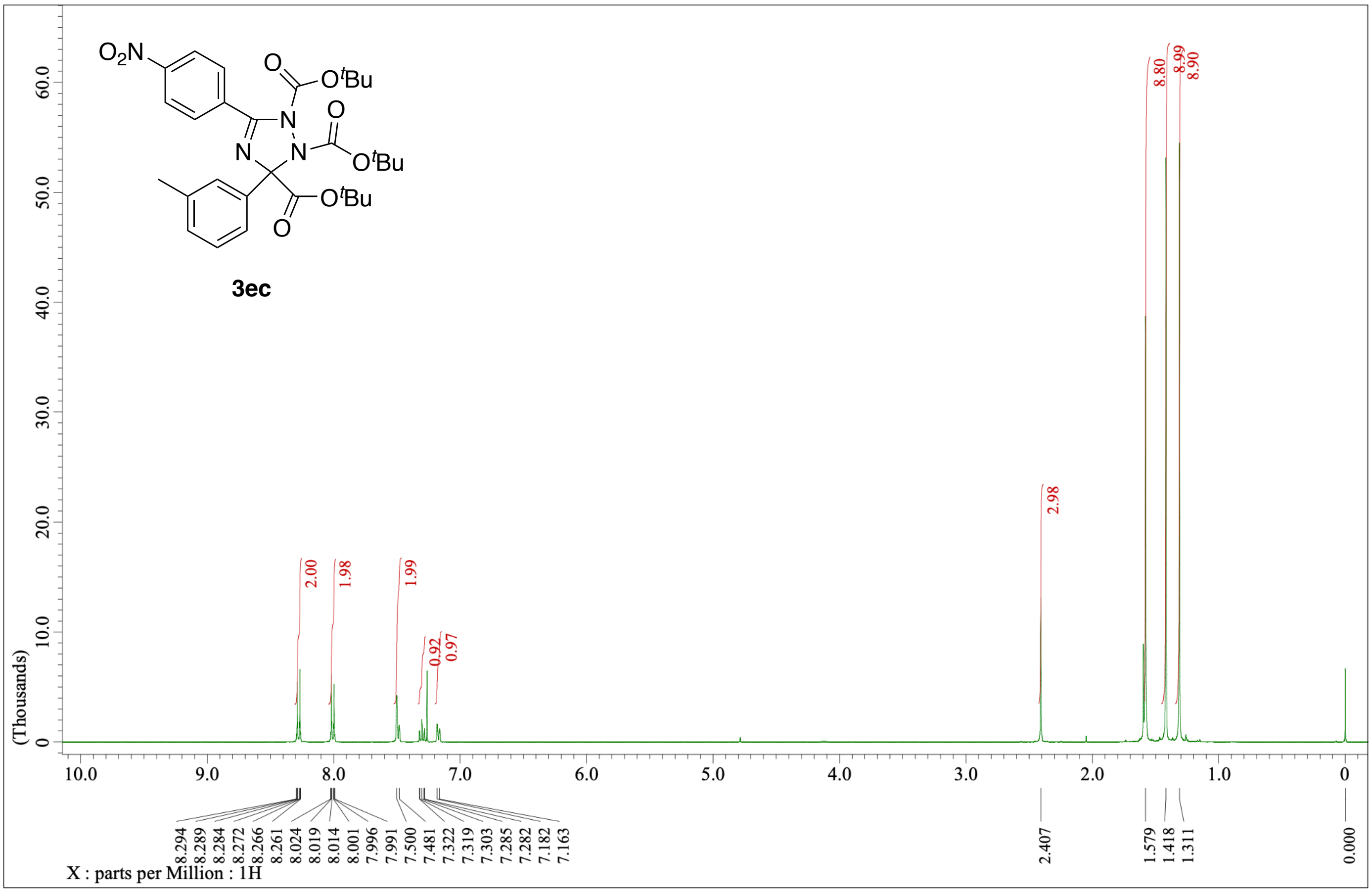

O2N
OtBu
OtBu
OtBu
3ec
2.00
1.98
1.99
0.92
0.97
2.98
8.80
8.99
8.90
(Thousands)
8.294 8.289 8.284 8.272 8.266 8.261 8.024 8.019 8.014 8.001 7.996 7.991 7.500 7.481 7.322 7.319 7.303 7.285 7.282 7.182 7.163
2.407
1.579 1.418 1.311
0.000
X : parts per Million : 1H

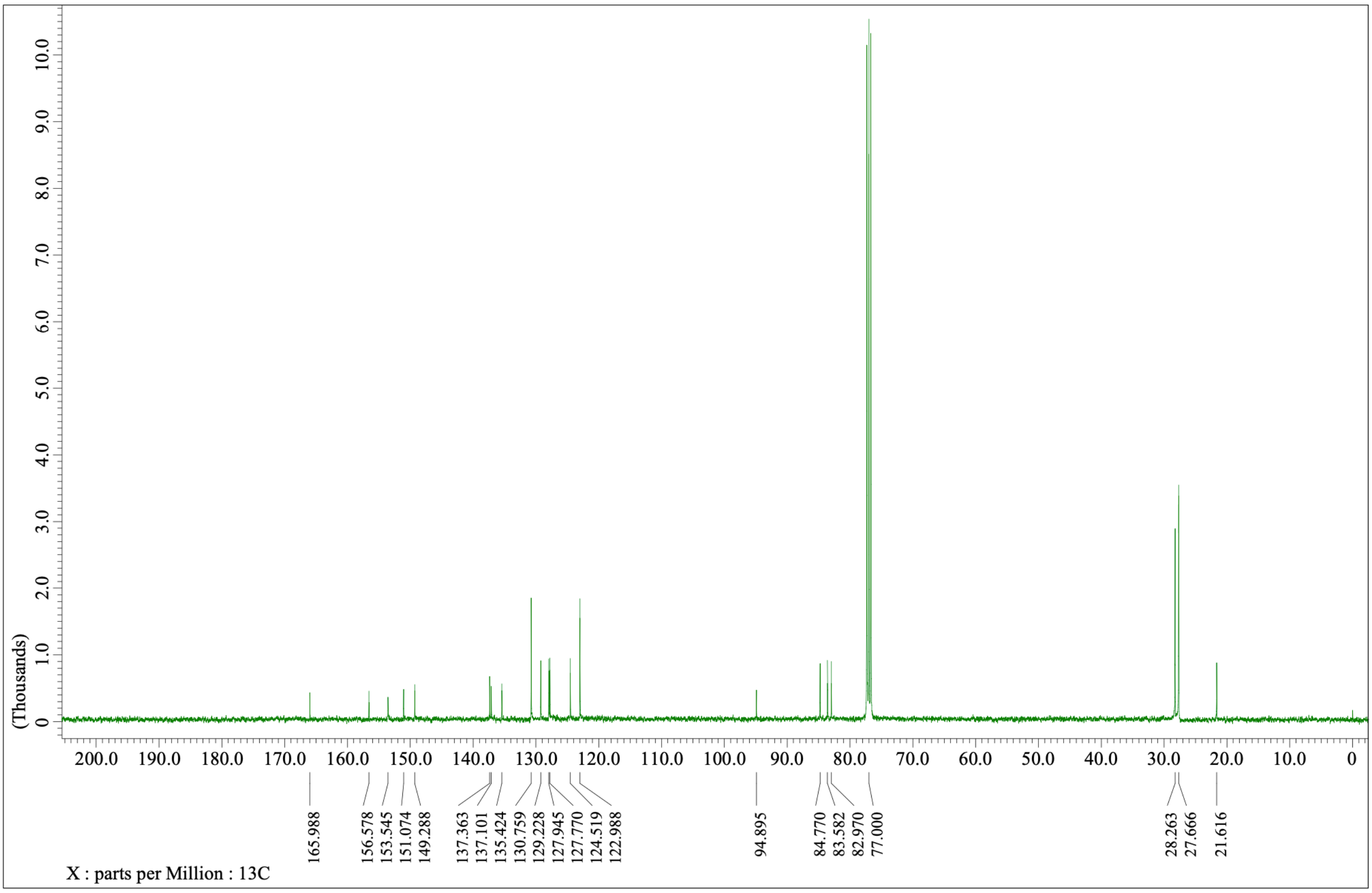

(Thousands)
165.988
156.578 153.545 151.074 149.288
137.363 137.101 135.424 130.759 129.228 127.945 127.770 124.519 122.988
94.895
84.770 83.582 82.970 77.000
28.263 27.666 21.616
X : parts per Million : 13C

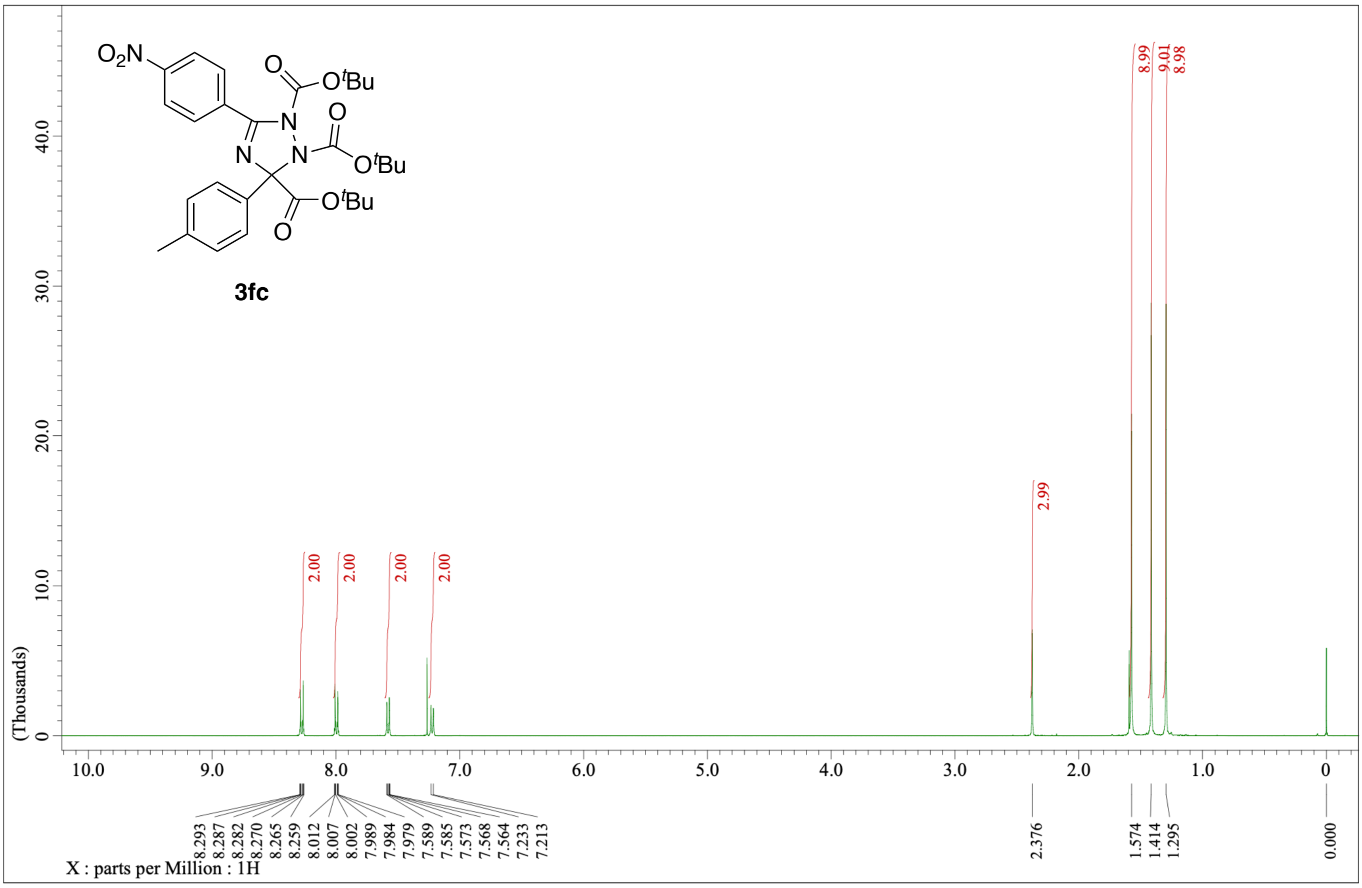

O_2N
O^{t}Bu
O^{t}Bu
O^{t}Bu
3fc
2.00
2.00
2.00
2.00
2.99
8.99
9.01
8.98
(Thousands)
8.293
8.287
8.282
8.270
8.265
8.259
8.012
8.007
8.002
7.989
7.984
7.979
7.589
7.585
7.573
7.568
7.564
7.233
7.213
2.376
1.574
1.414
1.295
0.000
X : parts per Million : 1H

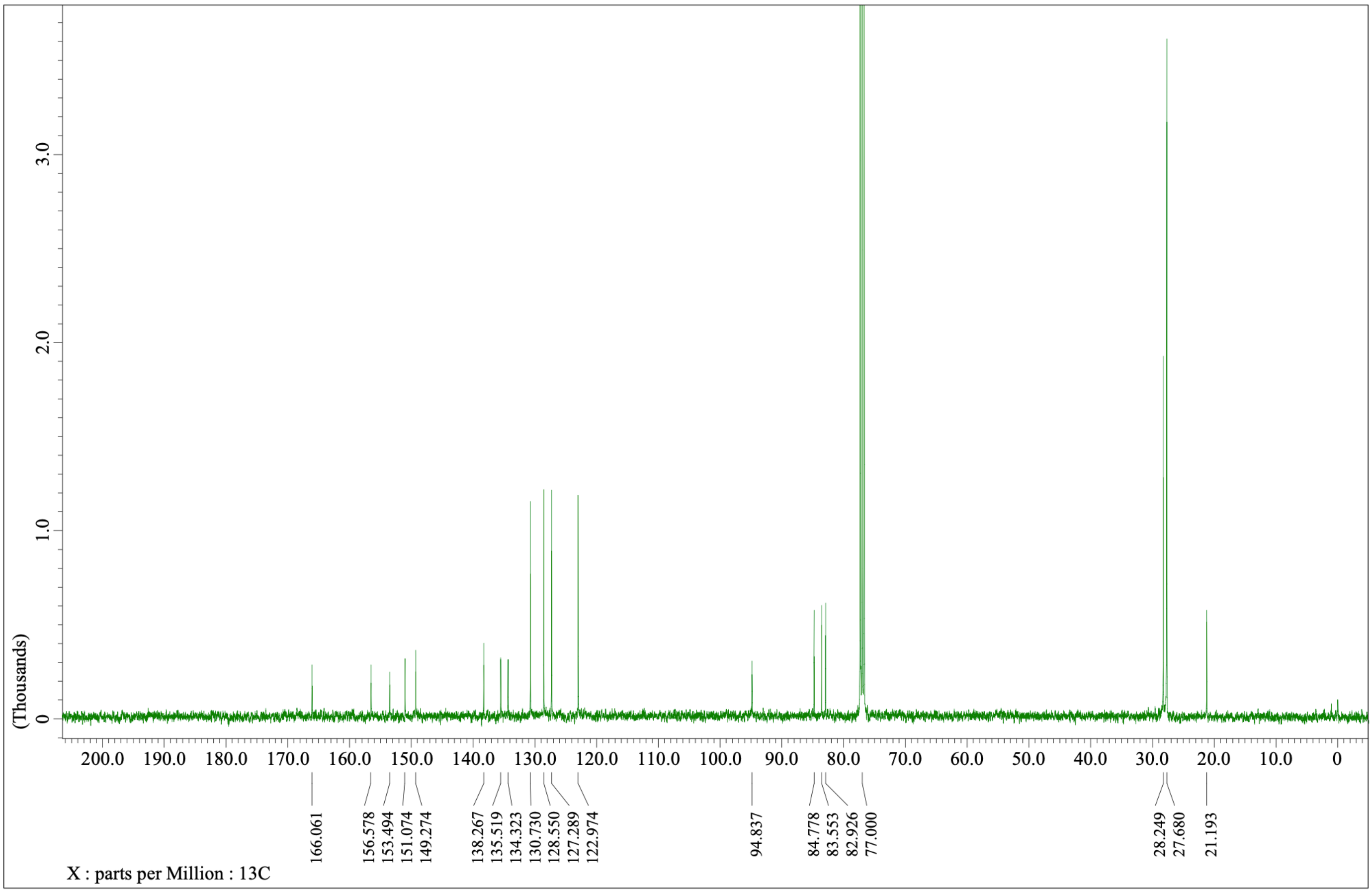

(Thousands)
166.061
156.578
153.494
151.074
149.274
138.267
135.519
134.323
130.730
128.550
127.289
122.974
94.837
84.778
83.553
82.926
77.000
28.249
27.680
21.193
X : parts per Million : 13C

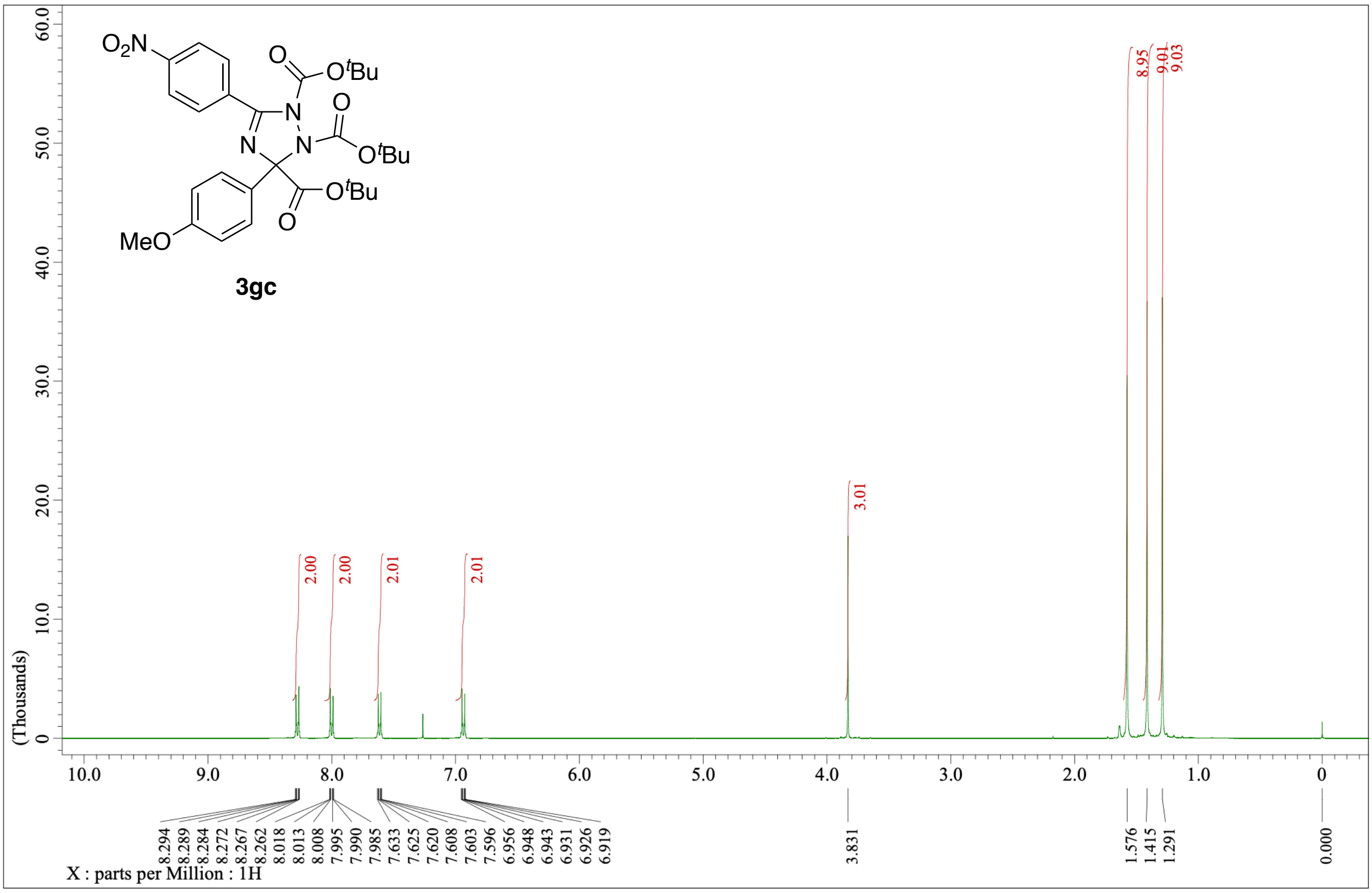

O_2N
O
O^tBu
N
N
N
O
O^tBu
O^tBu
O
MeO
3gc
2.00
2.00
2.01
2.01
3.01
8.95
9.01
9.03
(Thousands)
8.294 8.289 8.284 8.272 8.267 8.262 8.018 8.013 8.008 7.995 7.990 7.985 7.633 7.625 7.620 7.608 7.603 7.596 6.956 6.948 6.943 6.931 6.926 6.919
3.831
1.576 1.415 1.291
0.000
X : parts per Million : 1H

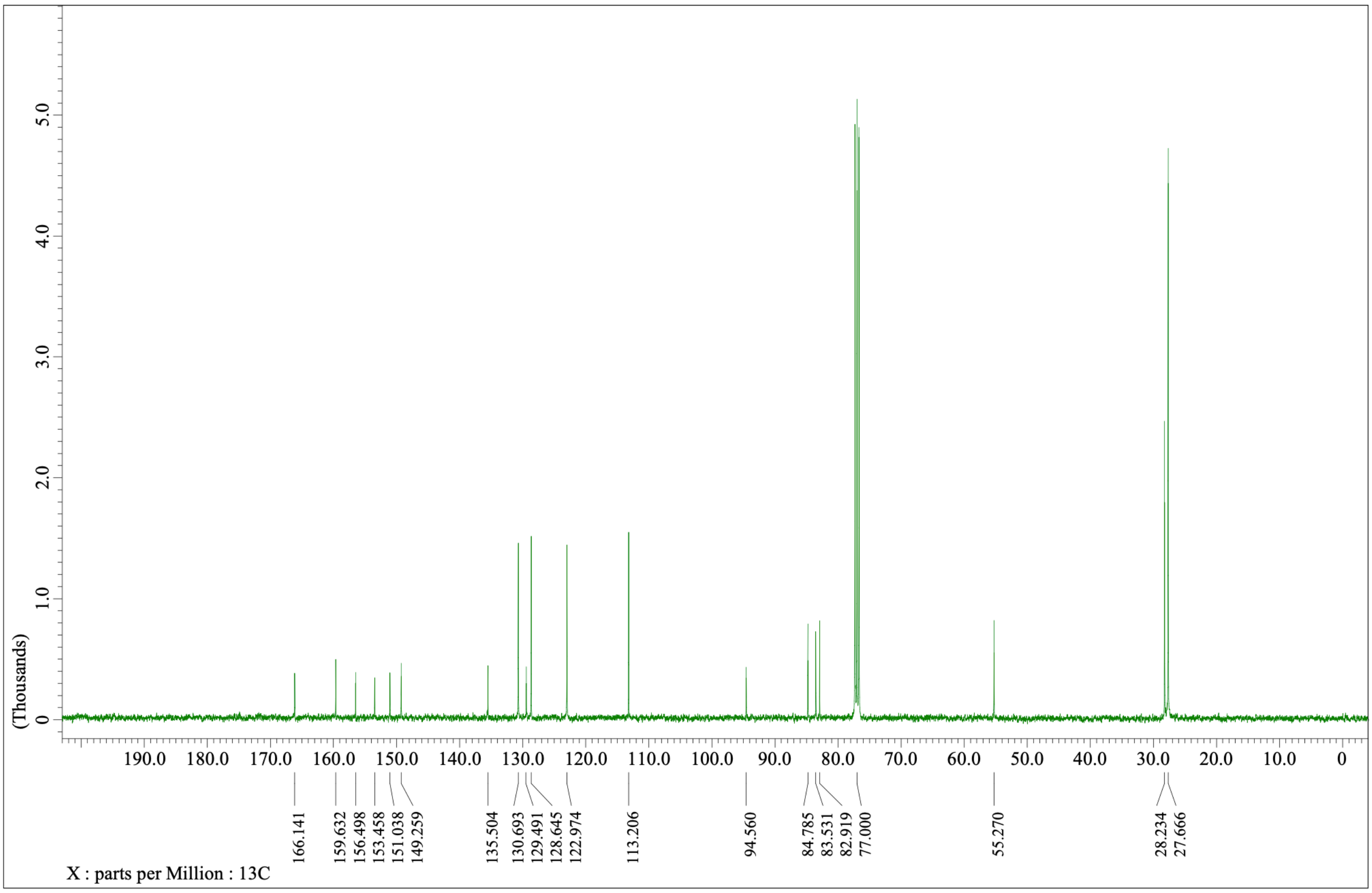

(Thousands)
166.141
159.632 156.498 153.458 151.038 149.259
135.504
130.693 129.491 128.645 122.974
113.206
94.560
84.785 83.531 82.919 77.000
55.270
28.234 27.666
X : parts per Million : 13C

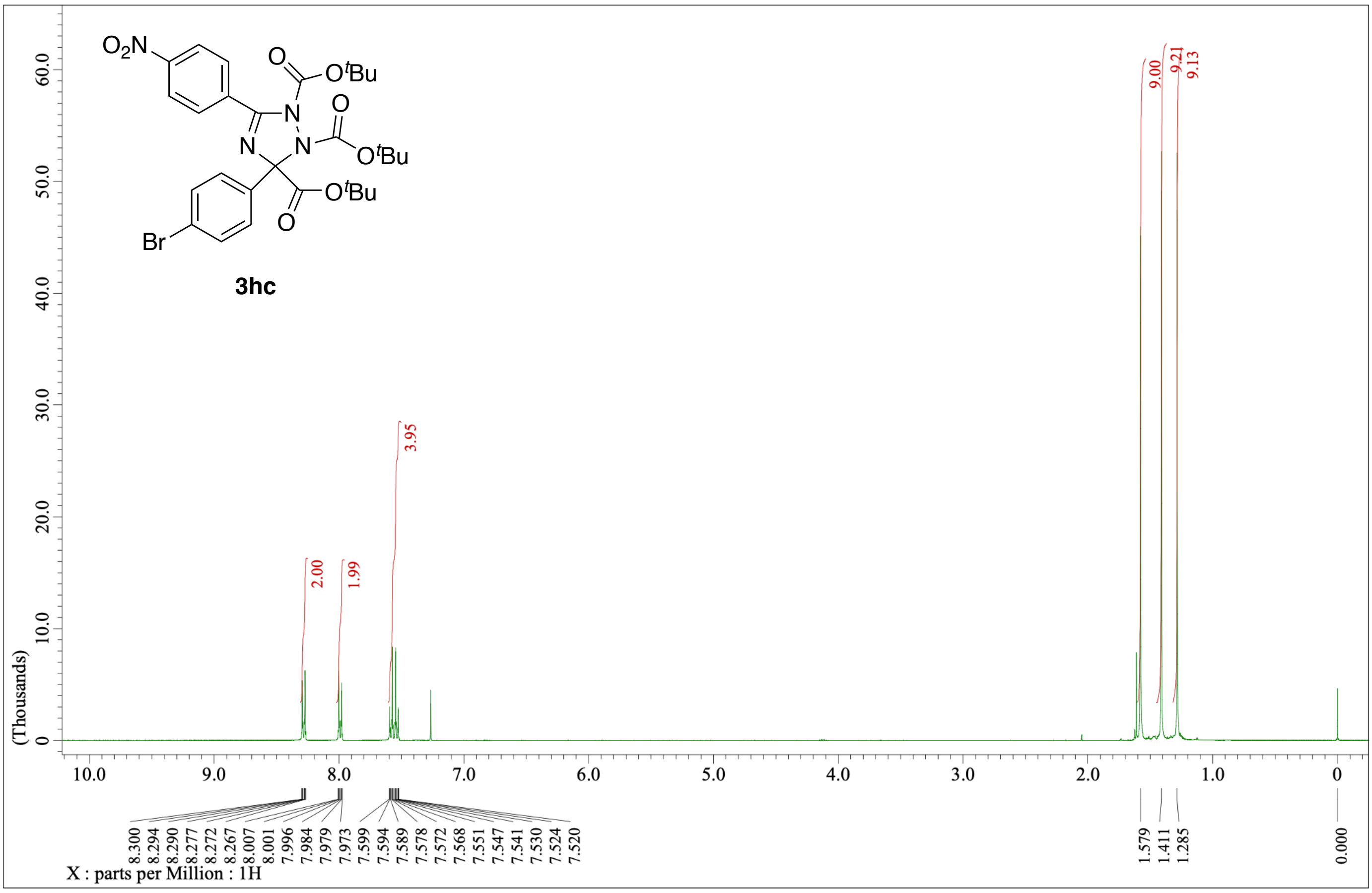
3hc
O2N
OtBu
Br
(Thousands)
X : parts per Million : 1H
2.00
1.99
3.95
9.00
9.21
9.13
8.300 8.294 8.290 8.277 8.272 8.267 8.007 8.001 7.996 7.984 7.979 7.973 7.599 7.594 7.589 7.578 7.572 7.568 7.551 7.547 7.541 7.530 7.524 7.520
1.579 1.411 1.285
0.000

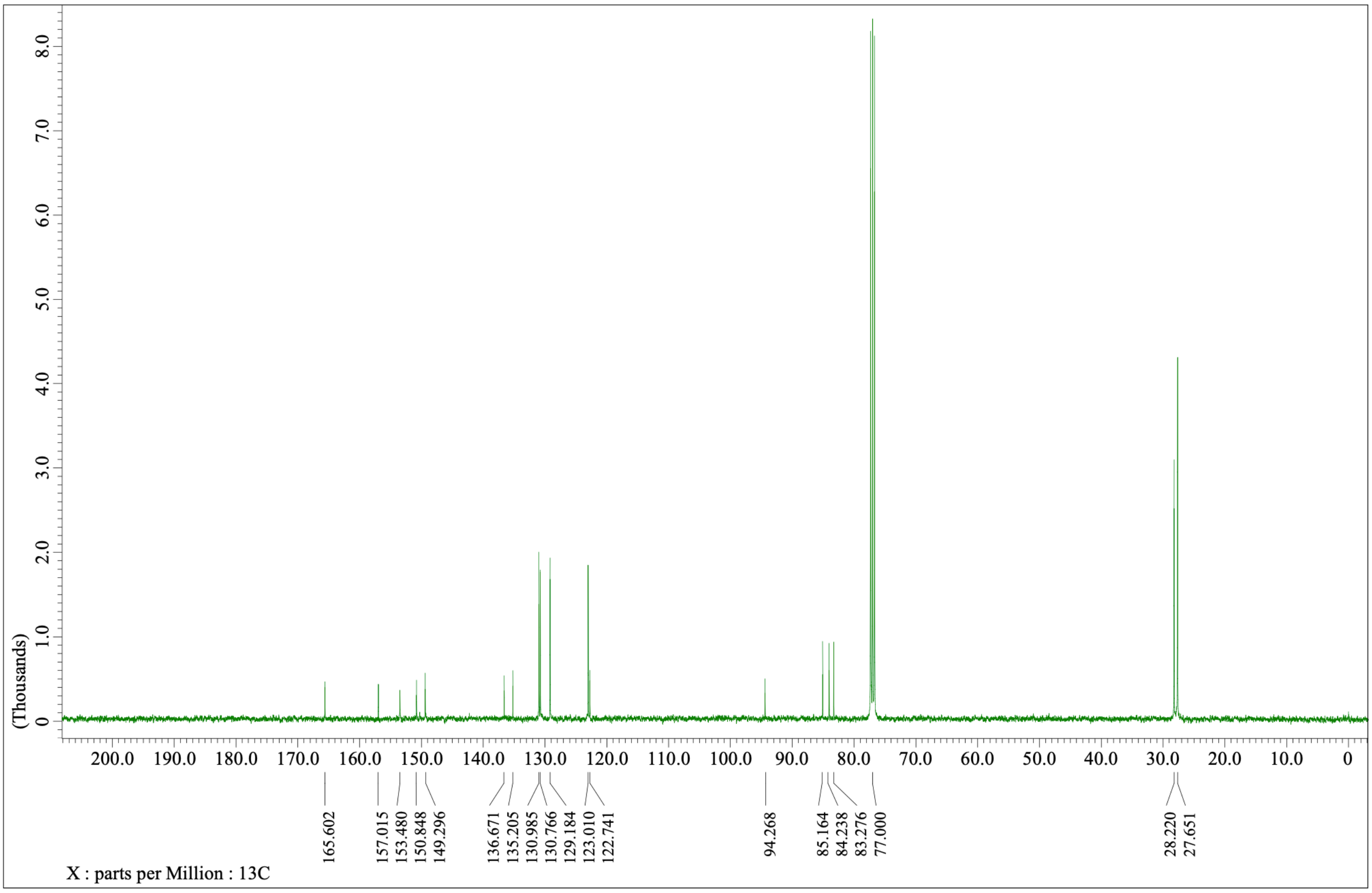
(Thousands)
X : parts per Million : 13C
165.602
157.015 153.480 150.848 149.296
136.671 135.205 130.985 130.766 129.184 123.010 122.741
94.268
85.164 84.238 83.276 77.000
28.220 27.651

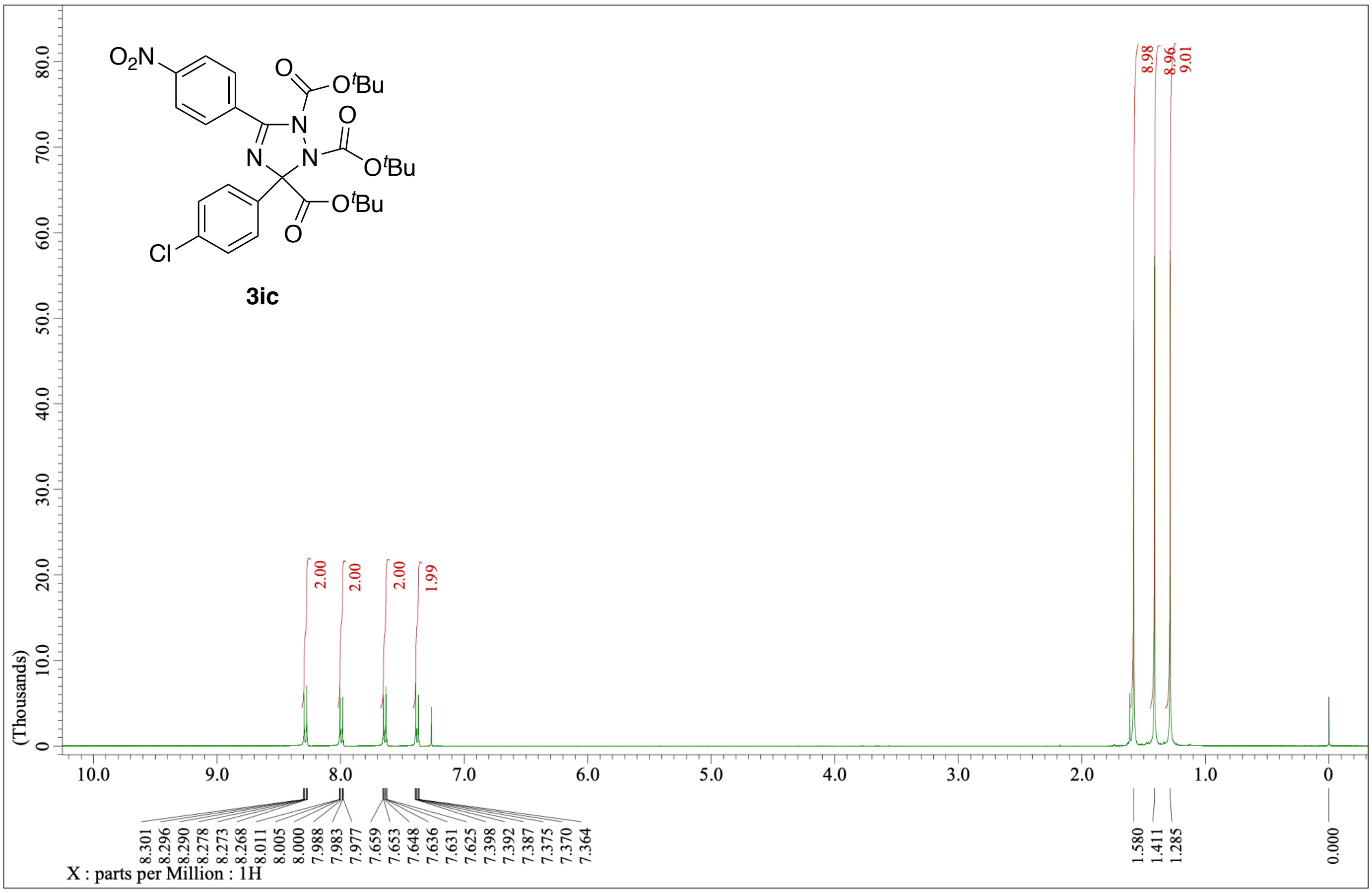

O_2N
O
O^{t}Bu
N
N
N
O^{t}Bu
O^{t}Bu
O
Cl
3ic
(Thousands)
80.0 70.0 60.0 50.0 40.0 30.0 20.0 10.0 0
2.00
2.00
2.00
1.99
8.98
8.96
9.01
10.0 9.0 8.0 7.0 6.0 5.0 4.0 3.0 2.0 1.0 0
8.301 8.296 8.290 8.278 8.273 8.268 8.011 8.005 8.000 7.988 7.983 7.977 7.659 7.653 7.648 7.636 7.631 7.625 7.398 7.392 7.387 7.375 7.370 7.364
1.580 1.411 1.285
0.000
X : parts per Million : 1H

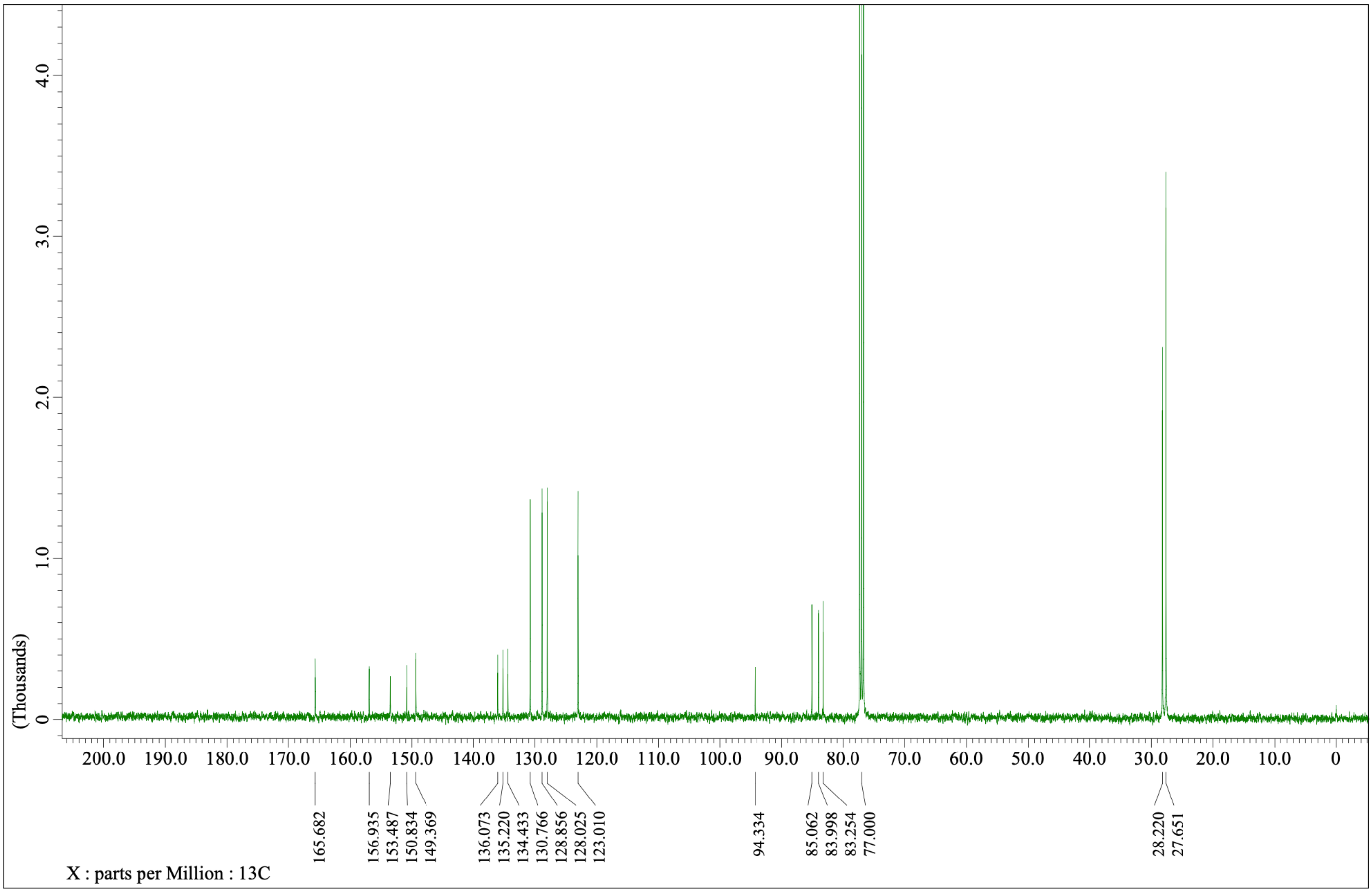

(Thousands)
4.0 3.0 2.0 1.0 0
200.0 190.0 180.0 170.0 160.0 150.0 140.0 130.0 120.0 110.0 100.0 90.0 80.0 70.0 60.0 50.0 40.0 30.0 20.0 10.0 0
165.682
156.935 153.487 150.834 149.369
136.073 135.220 134.433 130.766 128.856 128.025 123.010
94.334
85.062 83.998 83.254 77.000
28.220 27.651
X : parts per Million : 13C

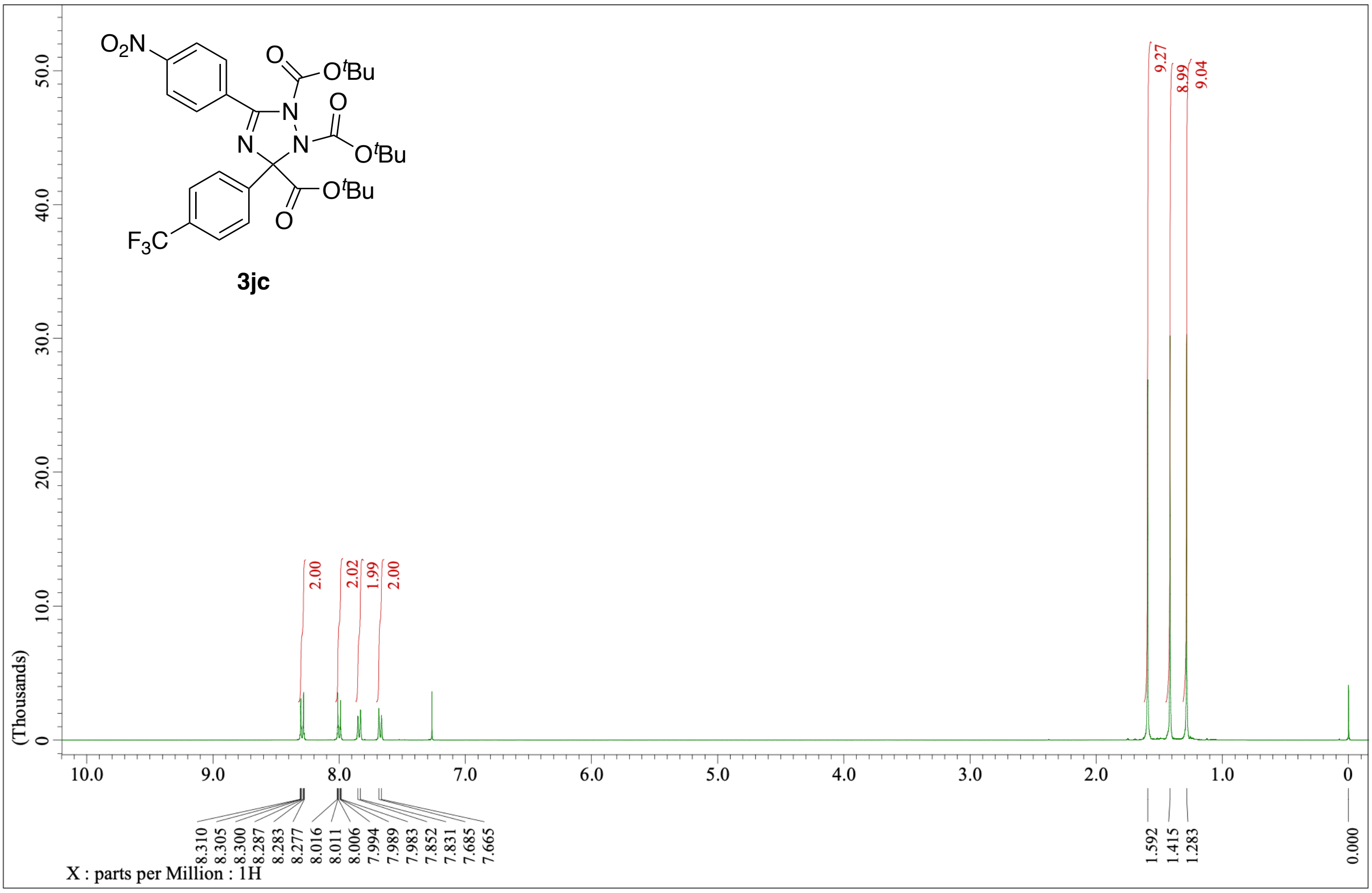
3jc
(Thousands)
X : parts per Million : 1H
2.00 2.02 1.99 2.00 9.27 8.99 9.04
8.310 8.305 8.300 8.287 8.283 8.277 8.016 8.011 8.006 7.994 7.989 7.983 7.852 7.831 7.685 7.665
1.592 1.415 1.283 0.000

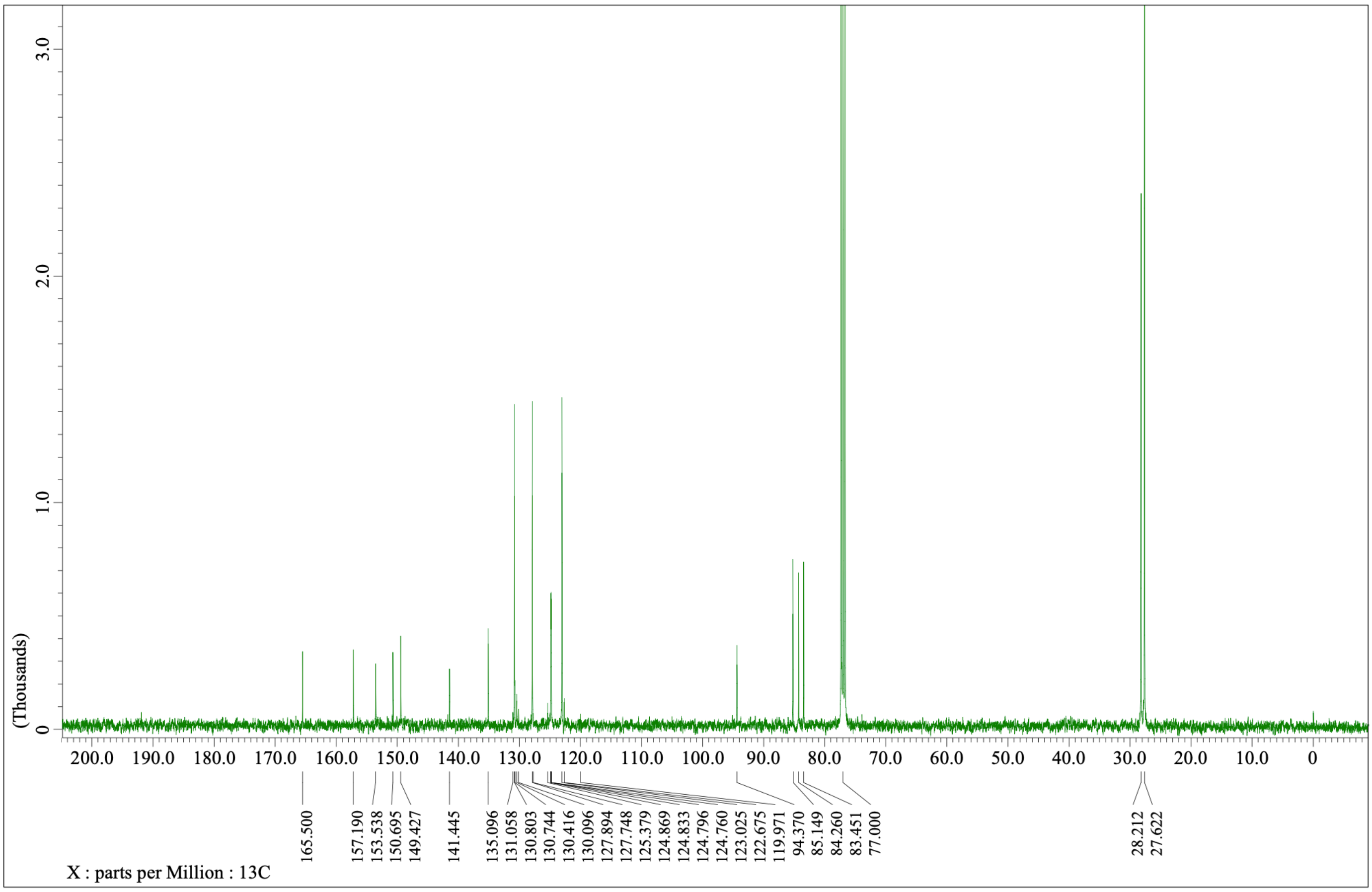
(Thousands)
X : parts per Million : 13C
165.500 157.190 153.538 150.695 149.427 141.445 135.096 131.058 130.803 130.744 130.416 130.096 127.894 127.748 125.379 124.869 124.833 124.796 124.760 123.025 122.675 119.971 94.370 85.149 84.260 83.451 77.000 28.212 27.622

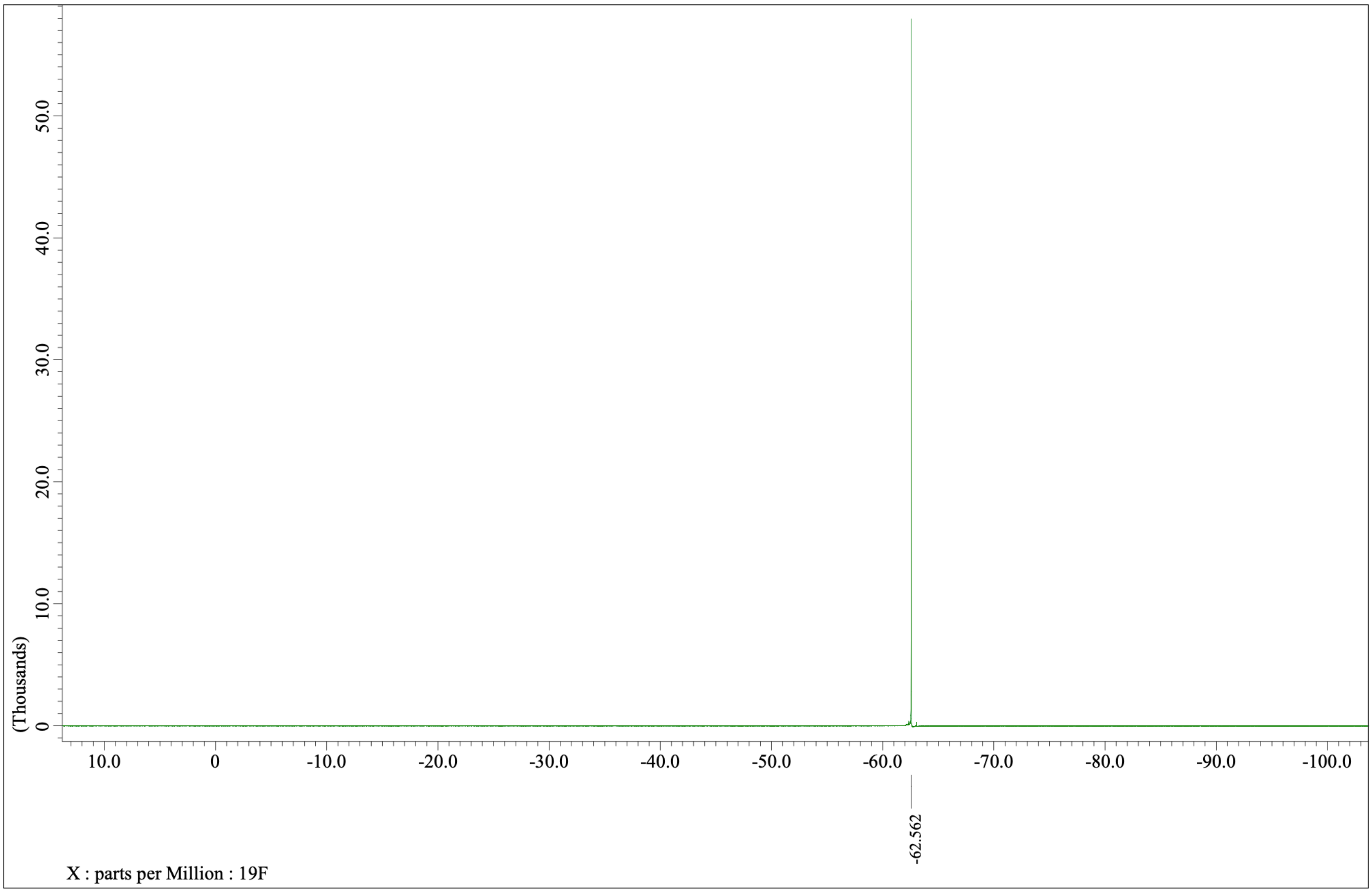

(Thousands)
0
10.0
20.0
30.0
40.0
50.0
10.0
0
-10.0
-20.0
-30.0
-40.0
-50.0
-60.0
-70.0
-80.0
-90.0
-100.0
-62.562
X : parts per Million : 19F

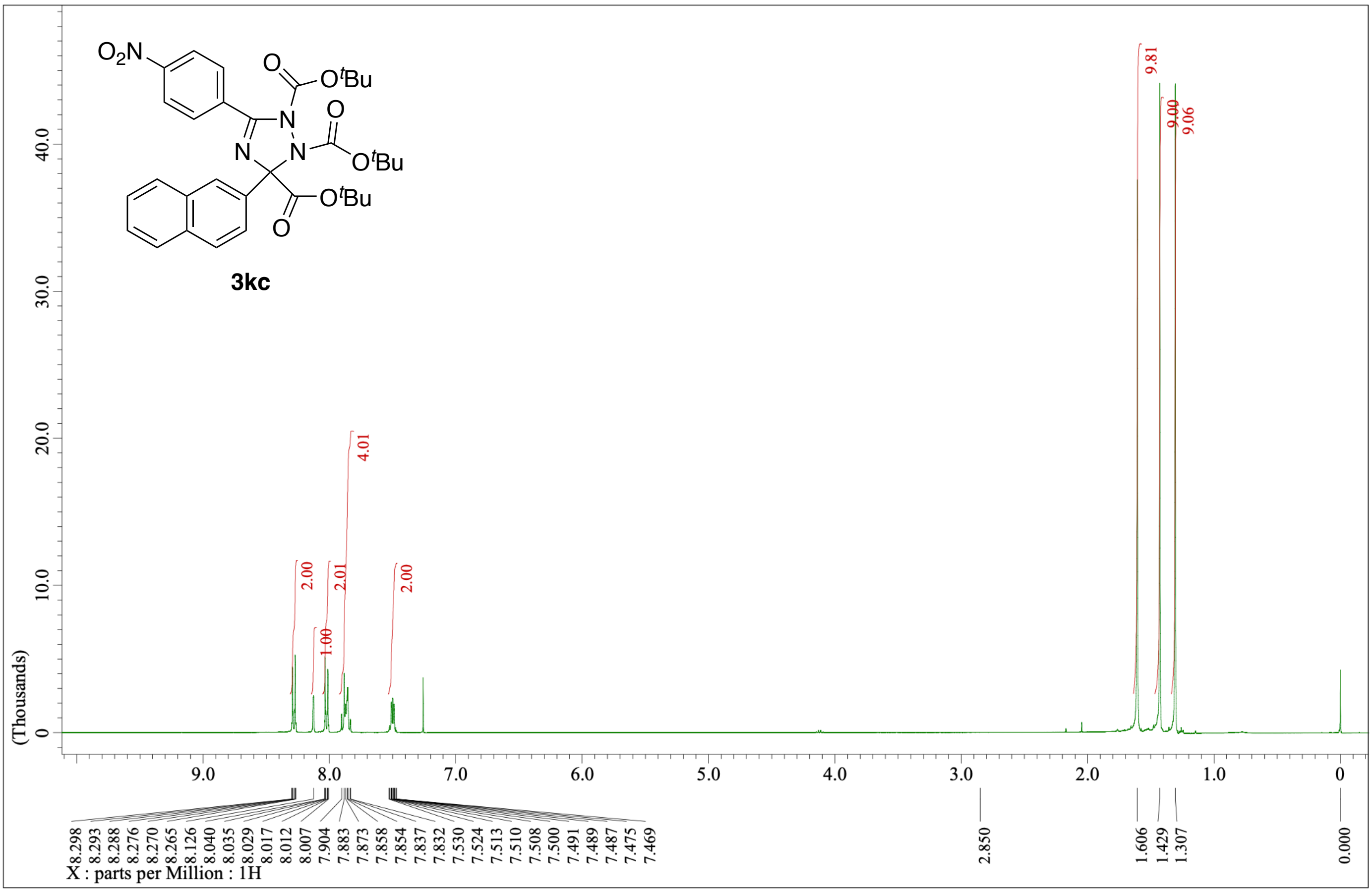

O2N
OtBu
N
3kc
(Thousands)
X : parts per Million : 1H
9.81
9.00
9.06
4.01
2.00
1.00
2.01
2.00
8.298 8.293 8.288 8.276 8.270 8.265 8.126 8.040 8.035 8.029 8.017 8.012 8.007 7.904 7.883 7.873 7.858 7.854 7.837 7.832 7.530 7.524 7.513 7.510 7.508 7.500 7.491 7.489 7.487 7.475 7.469
2.850
1.606 1.429 1.307
0.000

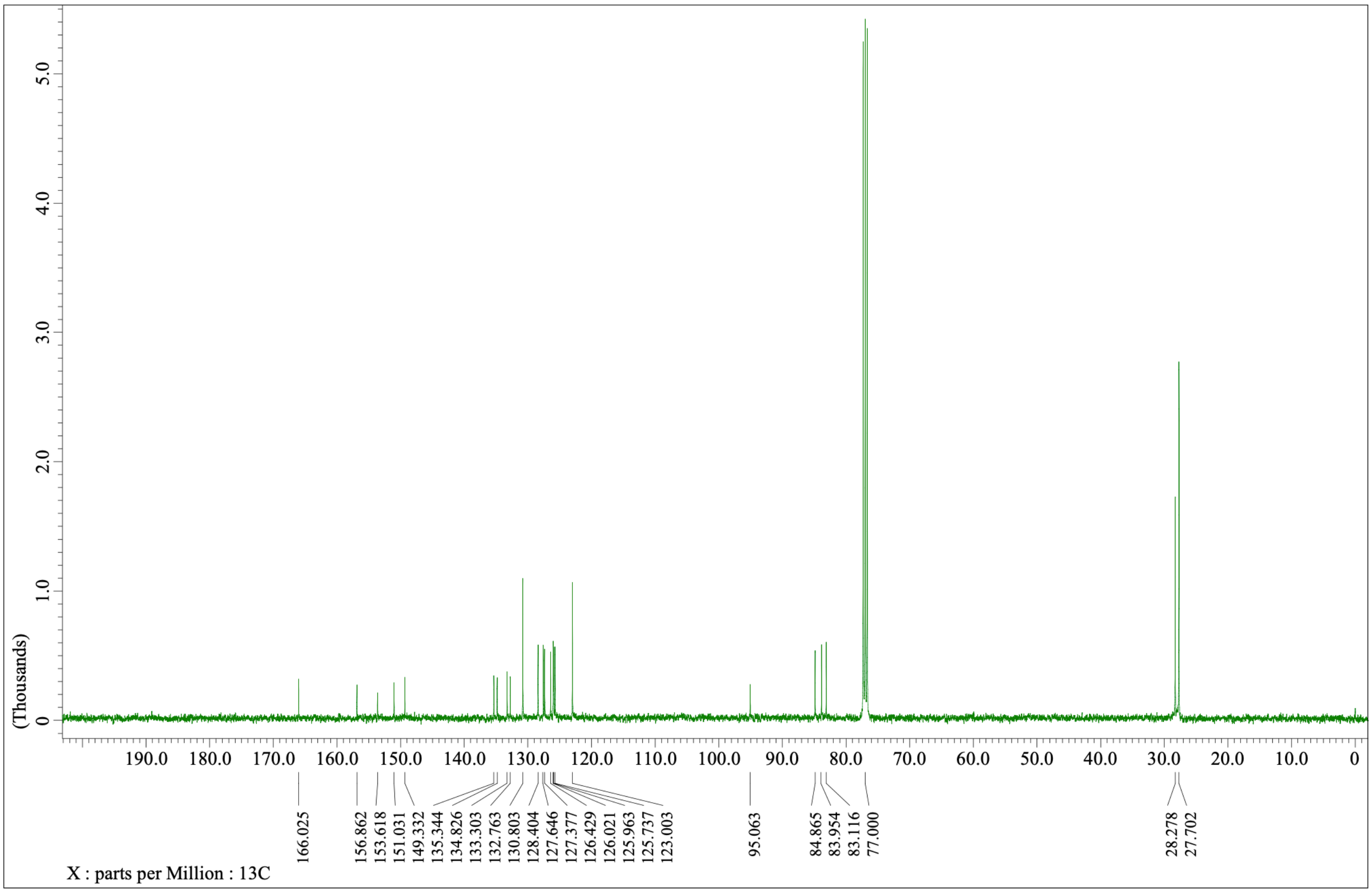

(Thousands)
X : parts per Million : 13C
166.025
156.862 153.618 151.031 149.332 135.344 134.826 133.303 132.763 130.803 128.404 127.646 127.377 126.429 126.021 125.963 125.737 123.003
95.063
84.865 83.954 83.116 77.000
28.278 27.702

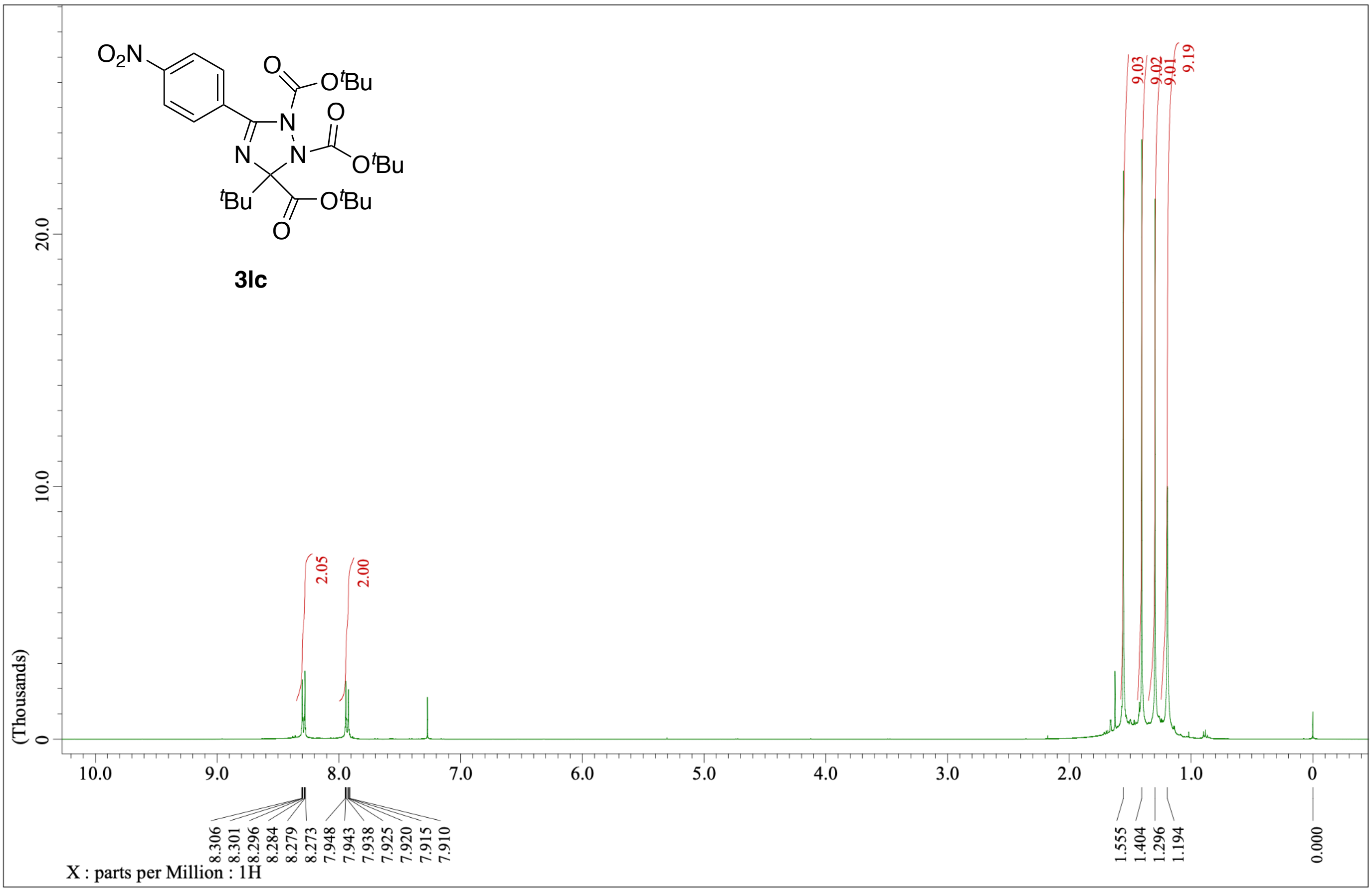

O2N
O
OtBu
O
N
N
N
OtBu
tBu
OtBu
O
3lc
9.03
9.02
9.01
9.19
2.05
2.00
(Thousands)
20.0
10.0
0
10.0
9.0
8.0
7.0
6.0
5.0
4.0
3.0
2.0
1.0
0
8.306
8.301
8.296
8.284
8.279
8.273
7.948
7.943
7.938
7.925
7.920
7.915
7.910
1.555
1.404
1.296
1.194
0.000
X : parts per Million : 1H

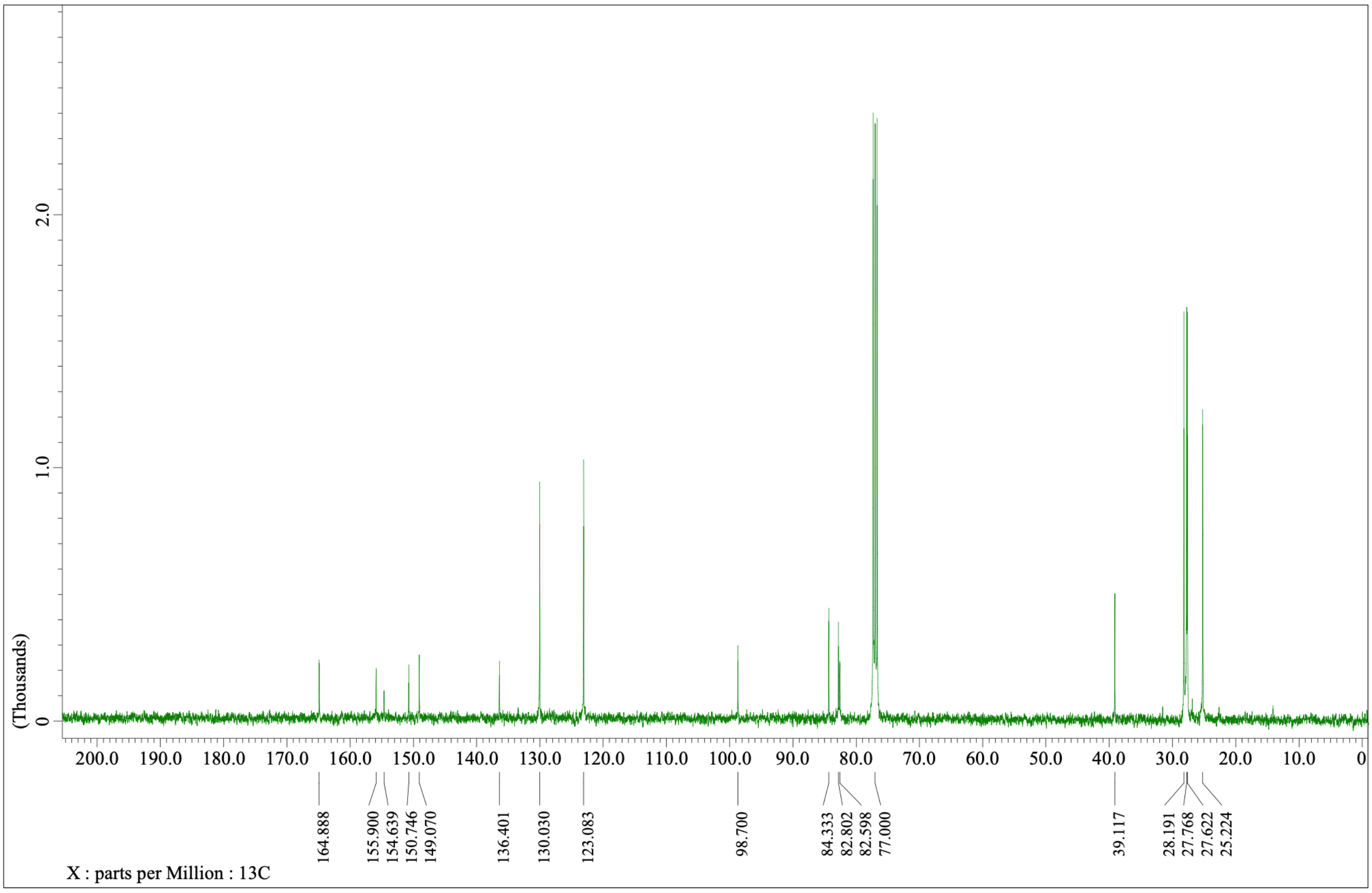

(Thousands)
2.0
1.0
0
200.0
190.0
180.0
170.0
160.0
150.0
140.0
130.0
120.0
110.0
100.0
90.0
80.0
70.0
60.0
50.0
40.0
30.0
20.0
10.0
164.888
155.900
154.639
150.746
149.070
136.401
130.030
123.083
98.700
84.333
82.802
82.598
77.000
39.117
28.191
27.768
27.622
25.224
X : parts per Million : 13C

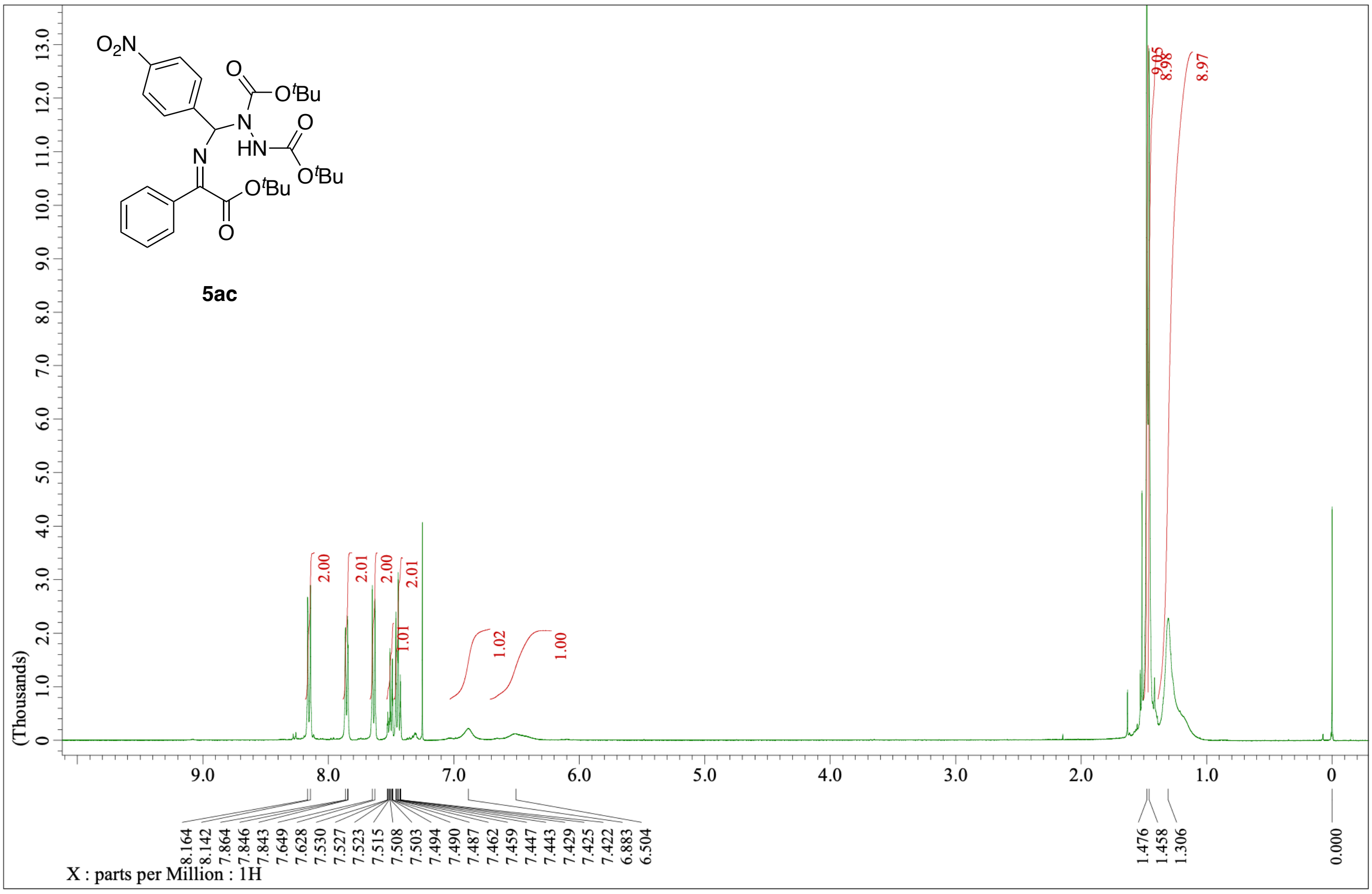

O2N
O
O^tBu
N
O
HN
O^tBu
N
O^tBu
O

5ac

(Thousands)
8.164
8.142
7.864
7.846
7.843
7.649
7.628
7.530
7.527
7.523
7.515
7.508
7.503
7.494
7.490
7.487
7.462
7.459
7.447
7.443
7.429
7.425
7.422
6.883
6.504
1.476
1.458
1.306
0.000
X : parts per Million : 1H

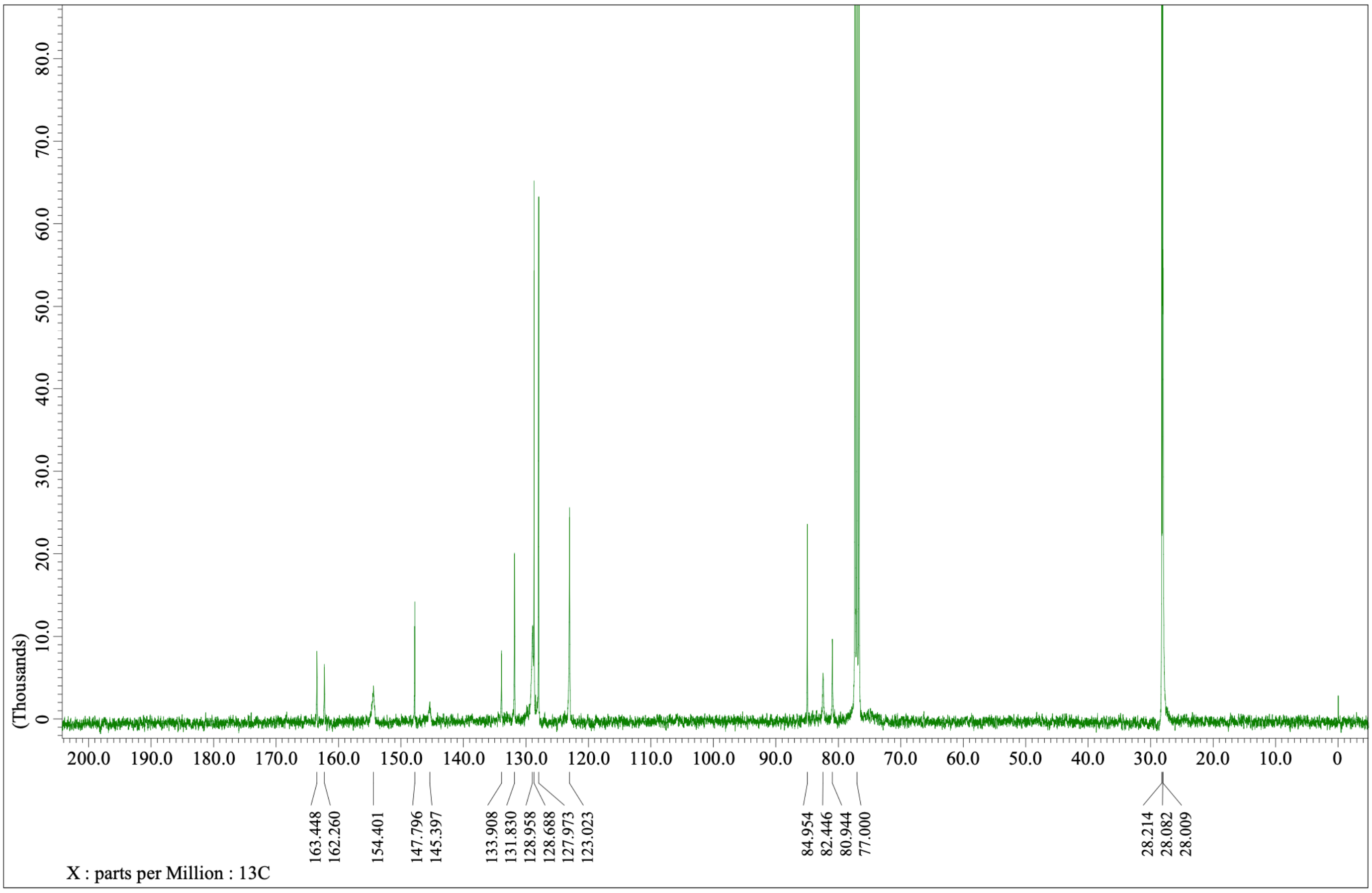

(Thousands)
163.448
162.260
154.401
147.796
145.397
133.908
131.830
128.958
128.688
127.973
123.023
84.954
82.446
80.944
77.000
28.214
28.082
28.009
X : parts per Million : 13C

・2D-NMR (HMBC)

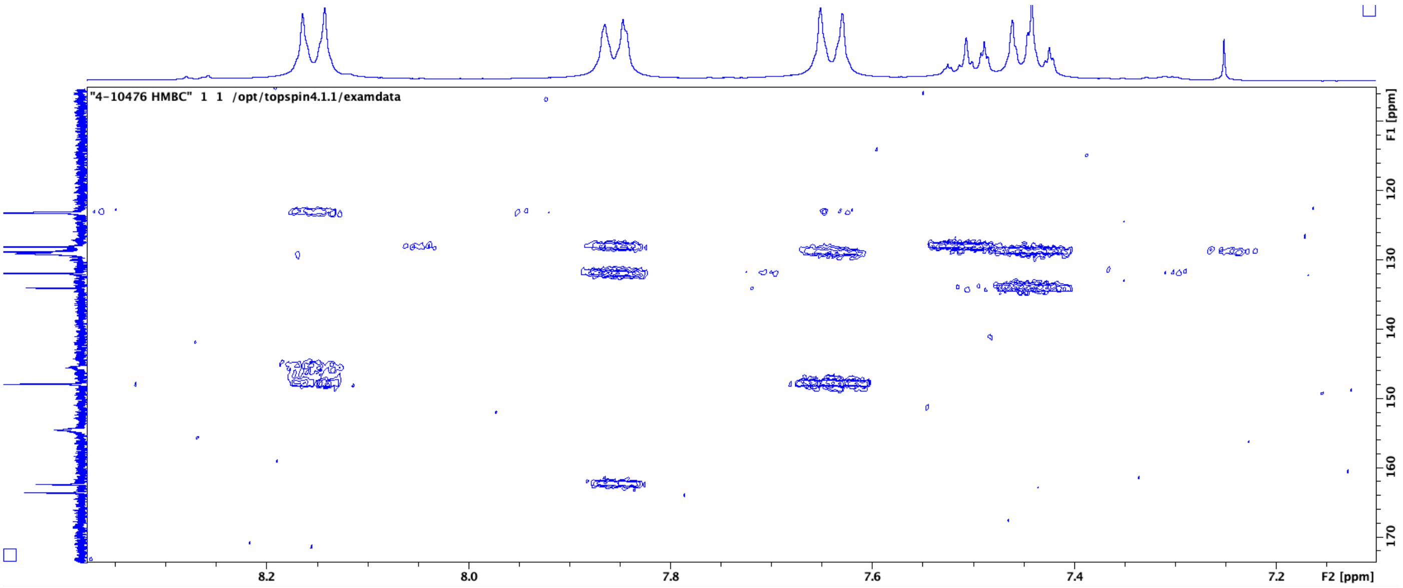

3ac (racemate)

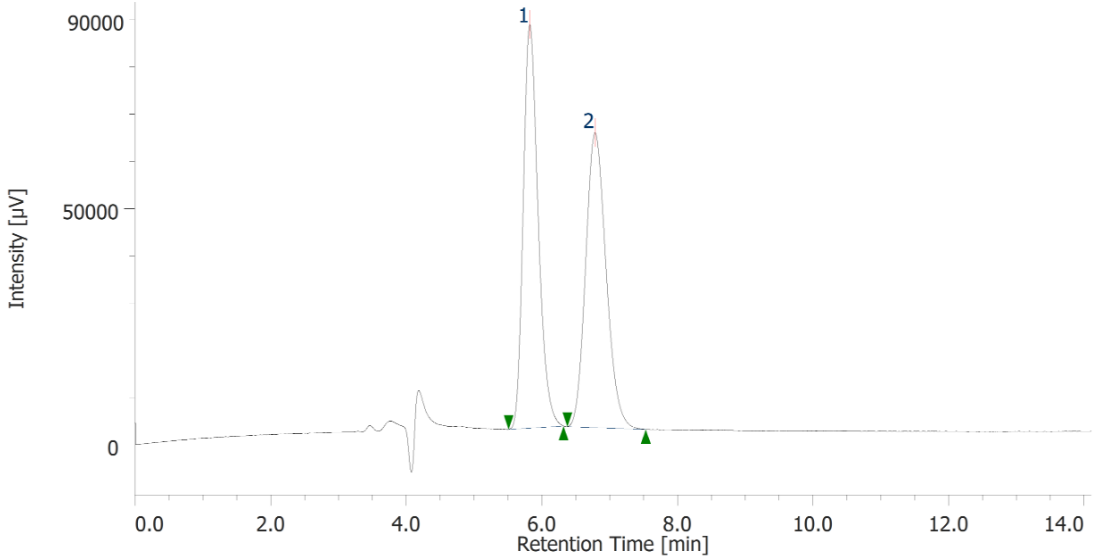

#	Peak Name	CH	tR [min]	Area [µV·sec]	Height [µV]	Area%	Height%
1	Unknown	10	5.822	1307127	85461	50.0	57.8
2	Unknown	10	6.783	1306558	62426	50.0	42.2

3ac (Scheme 6, starting from 1a)

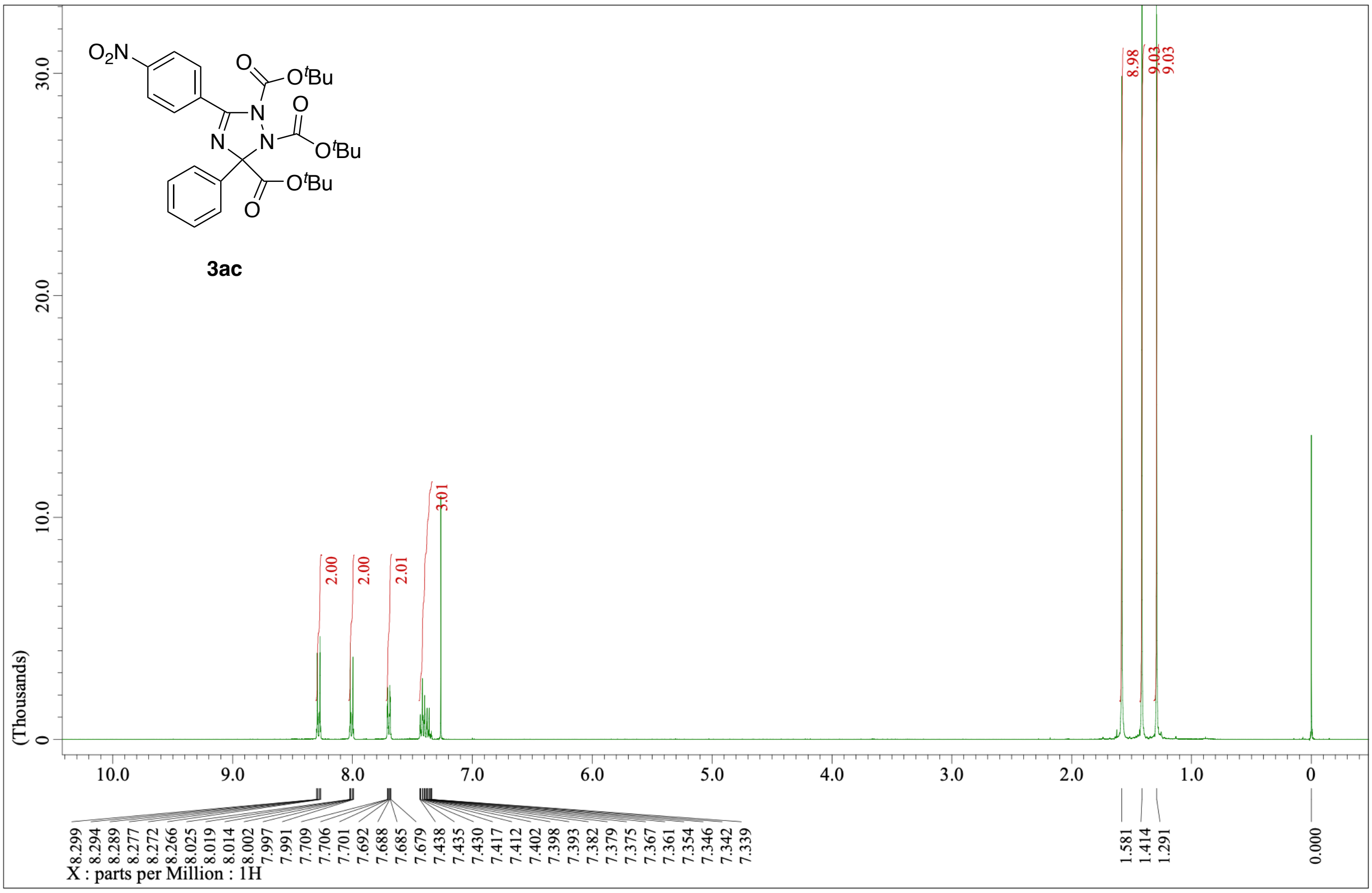

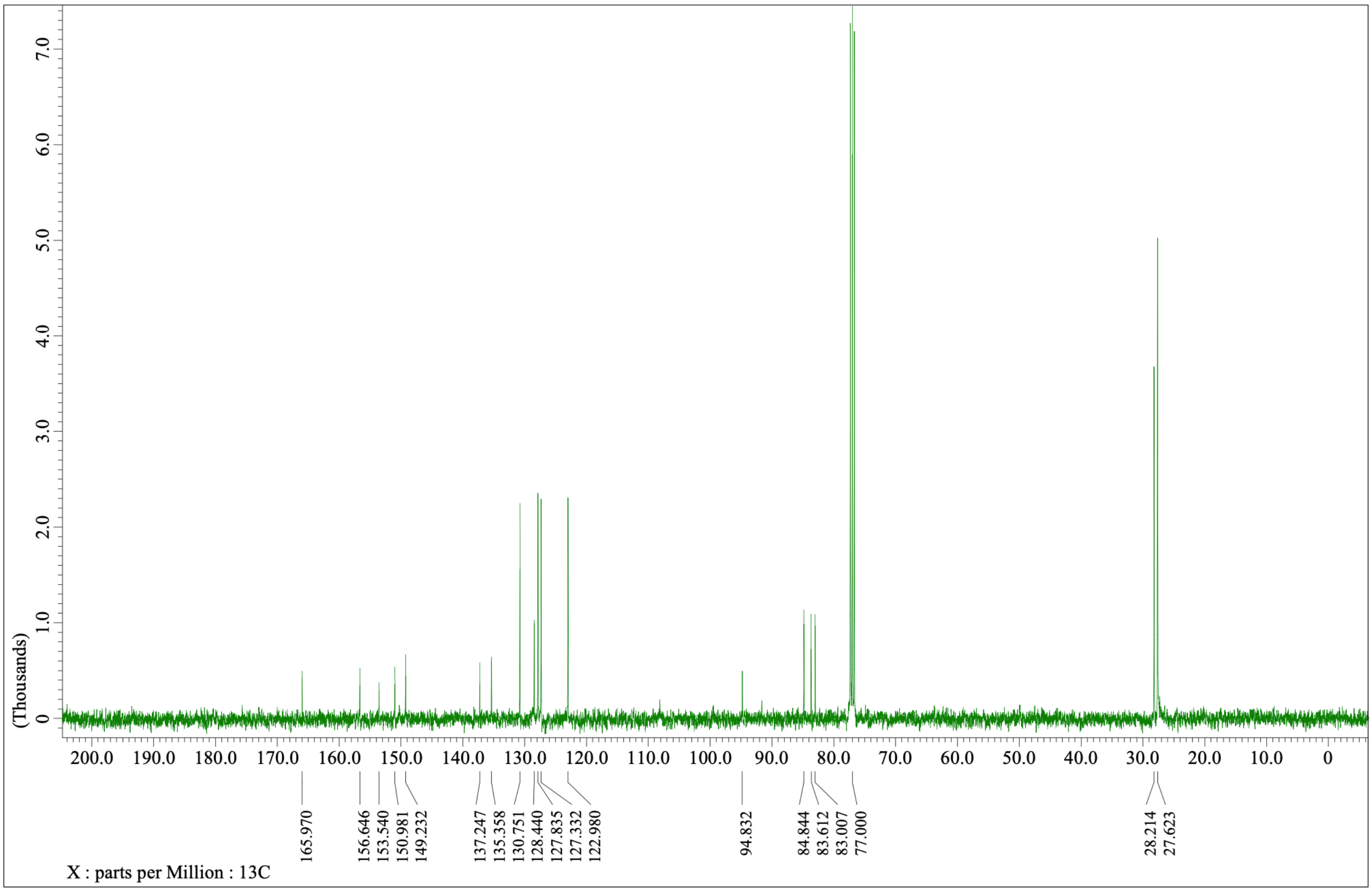

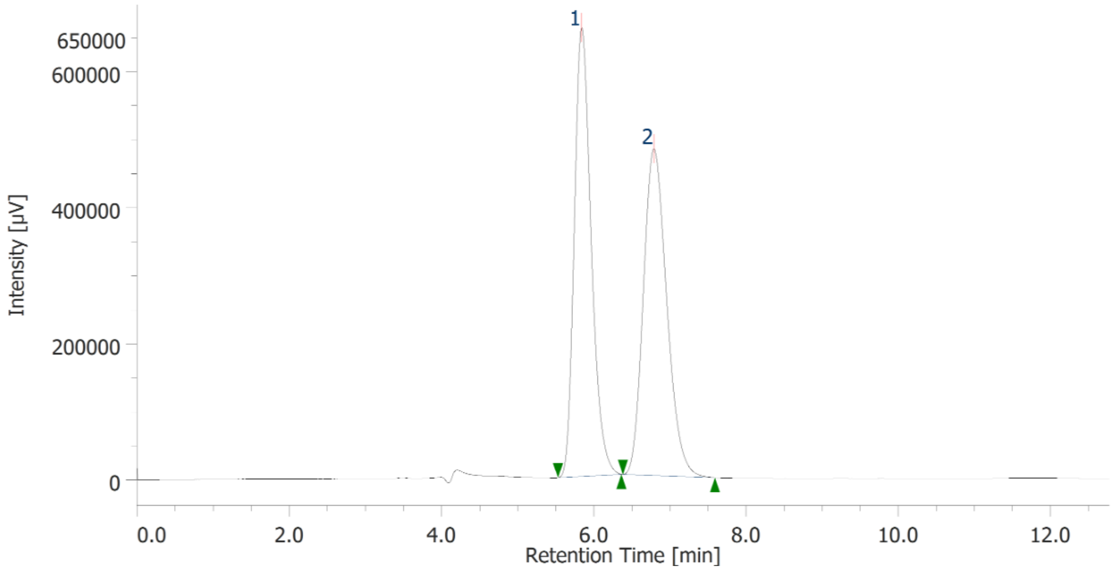

#	Peak Name	CH	tR [min]	Area [µV·sec]	Height [µV]	Area%	Height%
1	Unknown	11	5.838	10192332	659591	49.9	57.9
2	Unknown	11	6.788	10241875	480056	50.1	42.1

3ac (Scheme 7, starting from 1a')

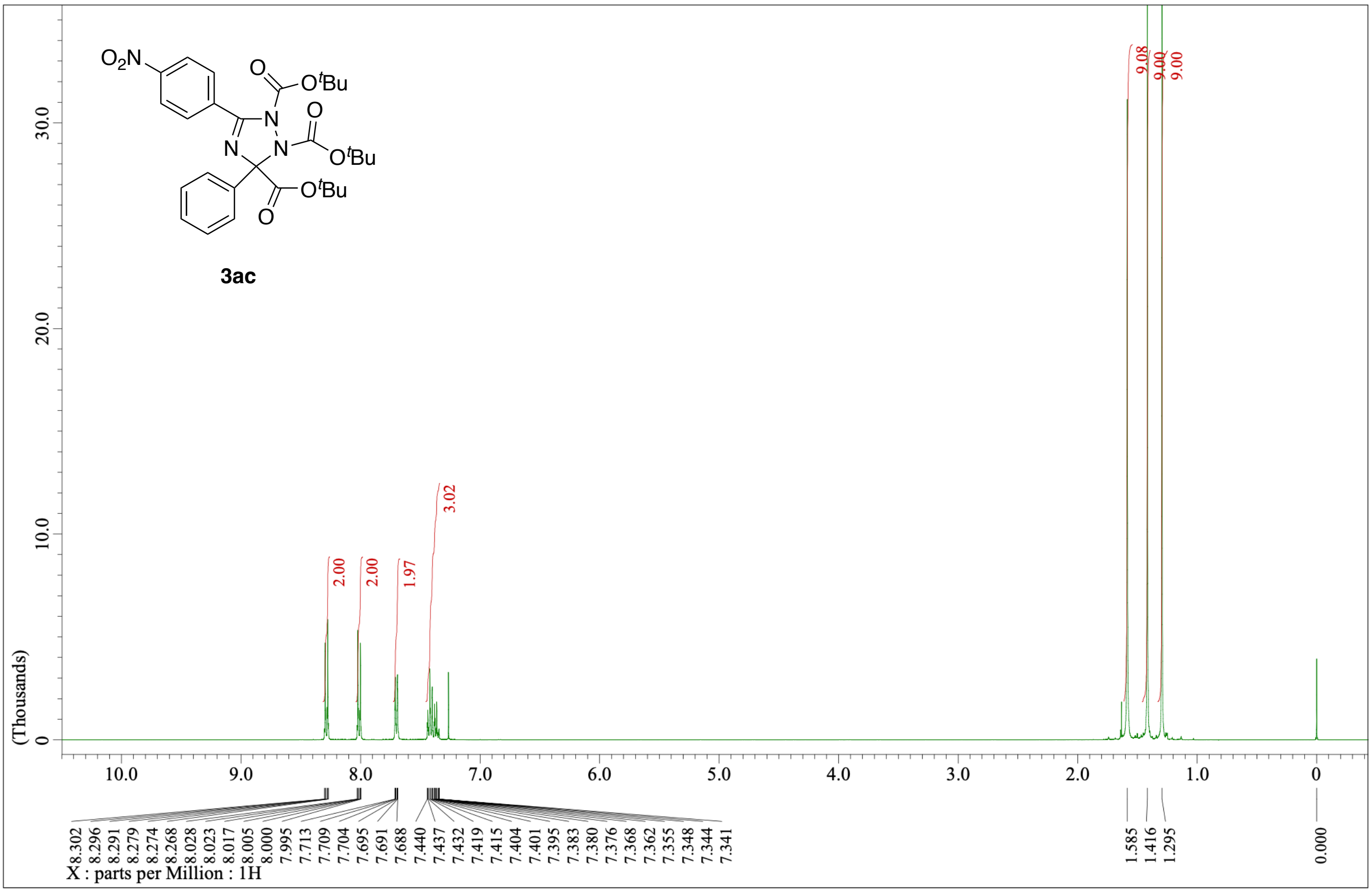

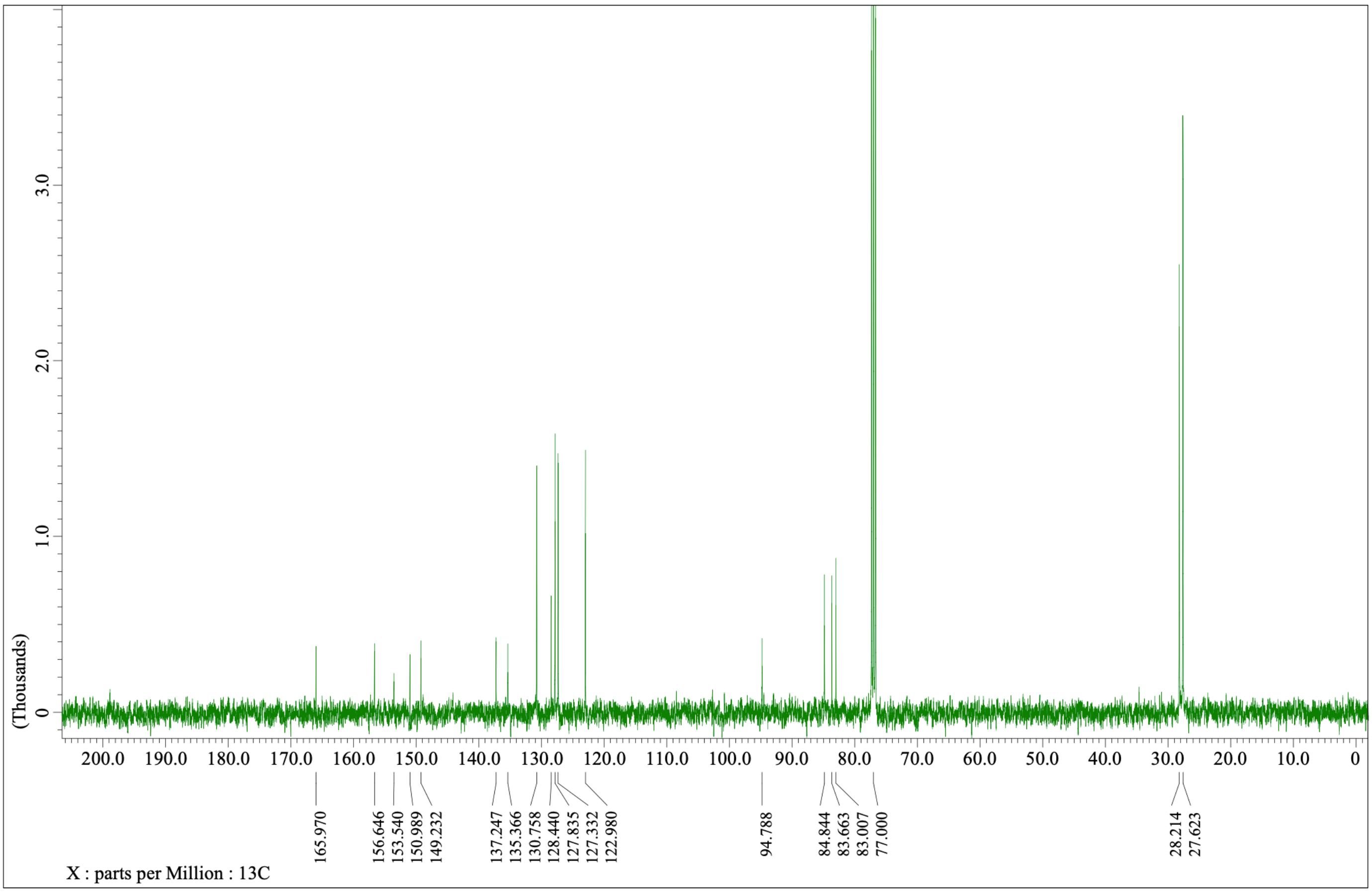

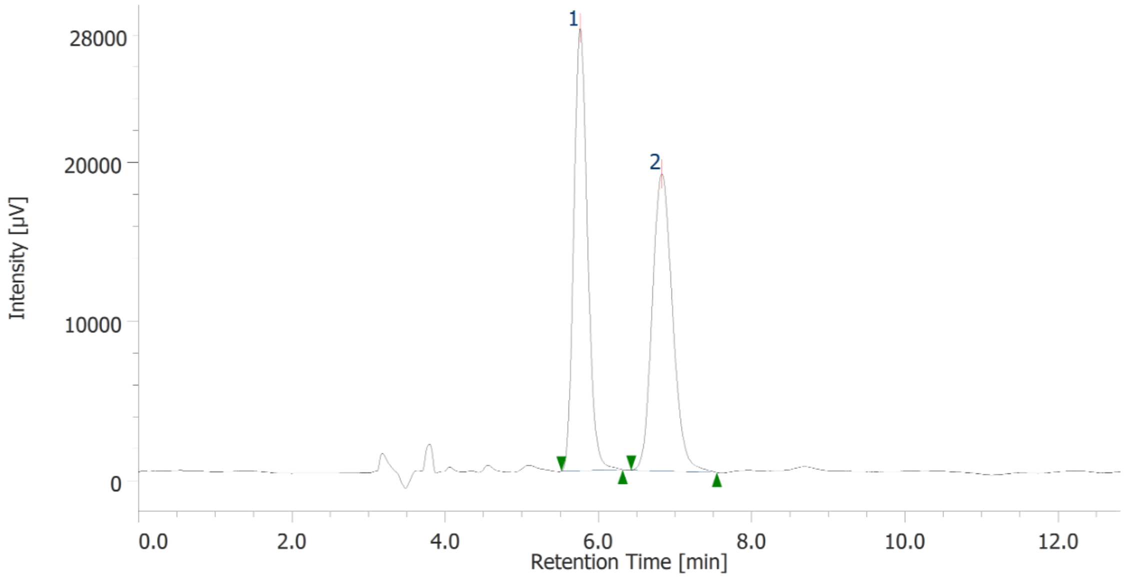

#	Peak Name	CH	tR [min]	Area [μV·sec]	Height [μV]	Area%	Height%
1	Unknown	3	5.758	345340	27834	50.1	59.9
2	Unknown	3	6.825	343465	18670	49.9	40.1
